# Description of 23 new species of the *Exocelina ekari*-group from New Guinea, with a key to all representatives of the species group (Coleoptera, Dytiscidae, Copelatinae)

**DOI:** 10.3897/zookeys.468.8506

**Published:** 2014-12-23

**Authors:** Helena Shaverdo, Katayo Sagata, Rawati Panjaitan, Herlina Menufandu, Michael Balke

**Affiliations:** 1 Naturhistorisches Museum, Burgring 7, 1010 Vienna, Austria; 2 Papua New Guinea Institute for Biological research (PNG-IBR), Goroka, Papua New Guinea; 3 Department of Biology, Faculty of Sciences and Mathematics, State University of Papua (UNIPA), Jalan Gunung Salju Amban, Manokwari 98314, West Papua, Indonesia; 4 Cenderawasih University, Faculty of Mathematics and Natural Science, Kampus Baru Waena Jayapura, Jayapura, Papua Province, Indonesia; 5 SNSB-Zoologische Staatssammlung München, Münchhausenstraße 21, D-81247 Munich, Germany and GeoBioCenter, Ludwig-Maximilians-University, Munich, Germany

**Keywords:** *Exocelina ekari*-group, Copelatinae, Dytiscidae, new species, New Guinea

## Abstract

Twenty three new species of *Exocelina* Broun, 1886 from New Guinea are described herein: *Exocelina bewaniensis* **sp. n.**, *Exocelina bismarckensis* **sp. n.**, *Exocelina craterensis* **sp. n.**, *Exocelina gorokaensis* **sp. n.**, *Exocelina herowana* **sp. n.**, *Exocelina jimiensis* **sp. n.**, *Exocelina kisli* **sp. n.**, *Exocelina ksionseki* **sp. n.**, *Exocelina lembena* **sp. n.**, *Exocelina mantembu* **sp. n.**, *Exocelina michaelensis* **sp. n.**, *Exocelina pinocchio* **sp. n.**, *Exocelina pseudoastrophallus* **sp. n.**, *Exocelina pseudobifida* **sp. n.**, *Exocelina pseudoedeltraudae* **sp. n.**, *Exocelina pseudoeme* **sp. n.**, *Exocelina sandaunensis* **sp. n.**, *Exocelina simbaiarea* **sp. n.**, *Exocelina skalei* **sp. n.**, *Exocelina tabubilensis* **sp. n.**, *Exocelina tariensis* **sp. n.**, *Exocelina vovai* **sp. n.**, and *Exocelina wannangensis* **sp. n.** All of them have been found to belong to the *Exocelina ekari*-group. An identification key to all known species of the group is provided, and important diagnostic characters (habitus, color, male antennae, protarsomeres 4–5, median lobes, and parameres) are illustrated. Data on the distribution of the new species and some already described species are given.

## Introduction

The *Exocelina ekari*-group, the largest species group of New Guinea *Exocelina*, was introduced by Balke et al. (2007) and Shaverdo et al. (2012) for 26 species. Listing several diagnostic characters of the group, we proposed a discontinuous outline of the median lobe of the aedeagus as the main diagnostic character of the group (for details, see Shaverdo et al. (2012), p. 4). We have recently detected 23 additional new species of this group, which are described here. Also, *Exocelina vladimiri* (Shaverdo, Sagata & Balke, 2005) has been found to belong to this group based on the strong morphological similarity to *Exocelina skalei* sp. n. These two species are assumed to present a separate morphological and genetic complex, which appears to be one of the basal lineages within the *Exocelina ekari*-group (Toussaint et al. 2014).

Our examination of additional material from the Western Highlands Province of Papua New Guinea showed that the type series of *Exocelina edeltraudae* Shaverdo, Hendrich & Balke, 2012 consists of two different species. Therefore, the new species *Exocelina pseudoedeltraudae* sp. n. is described and *Exocelina edeltraudae* redescribed.

The species identification key proposed in Shaverdo et al. (2012) is here modified to include the new representatives of the *ekari*-group. The distribution of the new species is mapped, and additional faunistic data are provided for some already described species.

At present, including this work, 88 species of the genus *Exocelina* Broun, 1886 are described from New Guinea (Balke 1998, 1999, Shaverdo and Balke 2014, Shaverdo et al. 2005, 2012, 2013) with 141 described species of *Exocelina* known worldwide (Nilsson 2013, Shaverdo and Balke 2014, Shaverdo et al. 2013). With 50 described species, the *Exocelina ekari*-group is the most speciose group of the genus.

Wiki-engine powered species pages were automatically created by ZooKeys with the publication of this article on species-id.net portal (see their links under the species names). These species pages provide, for example, high resolution art work and can be improved interactively should new data become available. The pages also have links to DNA sequence data depositories for the species which are submitted to Genbank by Toussaint et al. (2014). By providing these resources, we hope to help creating a more user-friendly, sustainable taxonomy as suggested by Riedel et al. (2013).

## Material and methods

The present work is based on the material from the following collections:

CASk collection of Andre Skale, Hof/Saale, Germany

MZB Museum Zoologicum Bogoriense, Cibinong, Indonesia (Dr H. Sutrisno)

NARI Papua New Guinea National Insect Collection, Port Moresby, PNG (Mr Mark Ero)

NHMW Naturhistorisches Museum Wien, Vienna, Austria (Dr M.A. Jäch)

ZSM Zoologische Staatsammlung München, Munich, Germany (Dr M. Balke)

All specimen data are quoted as they appear on the labels attached to the specimens. Label text is cited using quotation marks. Comments in square brackets are ours. We extracted DNA and obtained DNA sequence data for some of the species/specimens, marked with individual DNA extraction numbers (e.g., “256 DNA M. Balke”). All types of the herein described specimens are provided with red labels. Female specimens, identification of which is difficult or sometimes impossible, were included in the type series only when they were collected with males of respective species and did not show external morphological differences from them. If two or more morphologically similar species were collected together (i.e., males found together), their females were not included in the type series of the respective species but were instead mentioned under additional material. Species descriptions are based on the whole type series.

Some of the species treated herein are very similar to each other and, based on low overall genetic divergence, most likely also are of very recent origin (Toussaint et al. 2014). We have used constant morphological differences based on examined series as an indicator of interrupted gene flow and as an operational criterion to delineate biological species. However, we suggest that extensive population genetic work using genomic data might reveal many additional lineages that represent putative species in this highly structured geographic and geological setting.

Measurements were taken with a Wild M10 stereomicroscope. The following abbreviations were used: TL (total body length), TL-H (total body length without head), MW (maximum body width), and hw (handwritten). Number of the ventral setae of the male protarsomere 5 is given only for one specimen of each species, which was mounted on a glass slide (see below) for drawing. This character was found to be not very useful for species identification since it is possible to make a general statement on the setation pattern (short/long, dense/sparse) but not to count them with certainty at the magnification of normal dissecting microscopes. The potential phylogenetic information content of this character will be studied in a further work.

Drawings were made with the aid of a camera lucida attached to a Leica DM 2500 microscope. For detailed study and drawing, antennae, protarsi, and genitalia were removed and mounted on glass slides with DMHF (dimethyl hydantoin formaldehyde) as temporary preparations. The drawings were scanned and edited, using the software Adobe Illustrator CS5.1. Arrangement of the figures follows the species order in the key.

The terminology to denote the orientation of the genitalia (ventral for median lobe and dorsal and external for paramere) follows Miller and Nilsson (2003). The terminology on the structure of the prosternum follows Larson et al. (2000). Administrative divisions of Indonesia and Papua New Guinea follow information from Wikipedia (2014a, b, c).

### Checklist and distribution of species of the *Exocelina ekari*-group

Abbreviations: IN – Indonesia, PNG – Papua New Guinea. Only new species are numbered.

## Species descriptions

### 1. Exocelina bewaniensis

Taxon classificationAnimaliaColeopteraDytiscidae

Shaverdo, Menufandu & Balke sp. n.

http://zoobank.org/151F516D-6765-4625-8866-CDB50A5B4863

Figs 21 –23 , 49

Exocelina undescribed sp. MB1295: Toussaint et al. 2014: Supplementary figs 1–4, Tab. 2.

#### Type locality.

Papua New Guinea: Sandaun Province, Bewani Mts., approximately 03°05.13'S; 141°10.23'E.

#### Type material.

*Holotype*: male “Papua New Guinea: Sandaun, Bewani Stn., stream @ base of Bewani Mts., 200-300 m, 12.iv.2006, nr. 03.05.130S 141.10.227E, Balke & Sagata (PNG 37)” (ZSM). *Paratypes*: **Papua New Guinea:** 6 males, 5 females with the same label as the holotype, one male additionally with a green label “DNA M.Balke 1295” (NHMW, ZSM). 1 male, 2 females “Papua New Guinea: Sandaun, Bewani Stn., forest puddles @ base of Bewani Mts., 300 m, 12.iv.2006, nr. 03.05.130S 141.10.227E, Balke & Sagata (PNG 38)” (ZSM). **Indonesia: Papua Province: Mamberamo Raya Regency:** 1 male “IRIAN JAYA: Jayapura Prov. Mamberamo, Rouffaer Mts. Noiadi, 150 – 200m 17.3.1999, leg. Riedel” [approximately 02°46'S, 137°46'E] (NHMW). **Sarmi Regency:** 13 males, 7 females “Indonesia: Papua, Sarmi Waaf, N Foja Mts, waterfall in forest, 120m, 23.ix.2014, -2.3317793 138.7500472, Menufandu (Pap031)” (MZB, NHMW, ZSM). 6 males, 4 females “Indonesia: Papua, Sarmi area, 70m 25.ix.2014, -1.9713908 138.8491402, Menufandu (Pap032)” (MZB, ZSM). **Nabire/Paniai Regencies:** 7 males, 3 females “Indonesia: Papua, Road Nabire-Enarotali KM 111, 100m, 23.x.2011, 03 31.192S 135 55.426E, Uncen (PAP15)” (MZB, NHMW, ZSM). 6 males “Indonesia: Papua, Road Nabire-Enarotali KM 80, 250m, 22.x.2011, 03 33.860S 135 46.473E, Uncen (PAP12)” (MZB, NHMW, ZSM).

#### Diagnosis.

Beetle small, piceous, with paler clypeus and pronotal sides, shiny; pronotum without lateral bead; male antennomeres simple; male protarsomere 4 with large, thick, strongly curved anterolateral hook-like seta; median lobe with weak submedian constriction in ventral view; paramere with distinct notch on dorsal side and subdistal part short, rounded, with upper setae almost inconspicuous or rather distinct and lower relatively long, dense, thick, and flattened. The species is similar to *Exocelina soppi* Shaverdo, Hendrich & Balke, 2012, from which differs with larger male protarsomere 4, narrower apex of the median lobe, and paramere setae thicker and somewhat flattened.

#### Description.

*Size and shape*: Beetle small (TL-H 3.1–3.6 mm, TL 3.45–4.0 mm, MW 1.65–1.95 mm), with oblong-oval habitus, broadest at elytral middle. *Coloration*: Head brown to piceous, with paler clypeus and sometimes vertex; pronotum with dark brown to piceous disc and reddish brown to dark brown sides; elytra dark brown to piceous, sometimes with narrow reddish brown sutural lines; head appendages yellowish to reddish, legs distally darker, especially metathoracic legs (Fig. 49). Teneral specimens with coloration paler.

*Surface sculpture*: Head with dense punctation (spaces between punctures 1–3 times size of punctures), evidently finer and sparser anteriorly; diameter of punctures smaller than diameter of cells of microreticulation. Pronotum with much sparser and finer punctation than on head. Elytra with extremely sparse and fine punctation, almost invisible. Pronotum and elytra with weakly impressed microreticulation, dorsal surface shiny. Head with microreticulation stronger. Metaventrite and metacoxa distinctly microreticulate, metacoxal figs with longitudinal strioles and transverse wrinkles. Abdominal ventrites with distinct microreticulation, strioles, and fine sparse punctation, coarser and denser on two last abdominal ventrites.

*Structures*: Pronotum without lateral bead or with weak traces of lateral bead. Base of prosternum and neck of prosternal process with distinct ridge, less rounded anteriorly, with small anterolateral extensions. Blade of prosternal process lanceolate, relatively broad, convex, with distinct lateral bead and few setae; neck and blade of prosternal process evenly jointed. Abdominal ventrite 6 broadly rounded.

*Male*: Antenna simple (Fig. 21A). Protarsomere 4 with large, thick, strongly curved anterolateral hook-like seta. Protarsomere 5 ventrally with anterior row of 11–13 and posterior row of 5–6 short setae (Fig. 21B). Abdominal ventrite 6 with 7–14 lateral striae on each side. Median lobe with weak submedian constriction in ventral view and elongate apex in lateral view (Fig. 21C, D). Upper margin of apex distinctly curved or almost straight in lateral view. Paramere with distinct notch on dorsal side and subdistal part short, rounded, with upper setae almost inconspicuous or rather distinct and lower relatively long, dense, thick, and flattened; setae of proximal part more or less numerous, thin (Fig. 21E).

*Holotype*: TL-H 3.4 mm, TL 3.7 mm, MW 1.8 mm.

*Female*: Without evident differences in external morphology from males, except for abdominal ventrite 6 without striae.

#### Variability

(Figs 21–23). *Exocelina bewaniensis* sp. n. is described using the material from three different regions (Fig. 53). The specimens from these regions demonstrate variability in size (from Bewani: TL-H 3.35–3.45 mm, TL 3.7–3.75 mm, MW 1.75–1.8 mm; Nabire-Enarotali: TL-H 3.1–3.4 mm, TL 3.45–3.85 mm, MW 1.65–1.85 mm; Noiadi: TL-H 3.6 mm, TL 4 mm, MW 1.95 mm), dorsal punctation (in specimens from Nabire-Enarotali, it is slightly coarser), shape of the median lobe (in the specimen from Noiadi, the median lobe with weaker submedian constriction in ventral view and upper margin of apex more straight in lateral view (Figs 21D, 22D, 23D), and setation of the paramere (in specimens from Bewani and Papua, subdistal and proximal setae more numerous, with upper subdistal setae very distinct (Figs 21E, 22E, 23E).

At first, we intended to describe the species with three subspecies as these morphological differences are evident and stable within each region, though insignificant. Finally, we have decided against this, bearing in mind that more material is needed from these regions (especially, from Noiadi) and the regions in-between for a conclusion whether they belong to the different subspecies or maybe even species.

#### Distribution.

Papua New Guinea: Sandaun Province; Indonesia: Papua Province: Sarmi, Mamberamo Raya and Nabire/Paniai Regencies (Fig. 53).

#### Etymology.

The name refers to Bewani Mts. where this species was discovered for the first time. The name is an adjective in the nominative singular.

### 2. Exocelina bismarckensis

Taxon classificationAnimaliaColeopteraDytiscidae

Shaverdo & Balke sp. n.

http://zoobank.org/37369706-7525-4975-A458-6055222EFE6E

Figs 15 , 43

Exocelina undescribed spp. MB1306, MB1369: Toussaint et al. 2014: Supplementary figs 1–4, Tab. 2.

#### Type locality.

Papua New Guinea: Eastern Highlands Province, Akameku - Brahmin, Bismarck Range, 05°56.80'S; 145°22.24'E.

#### Type material.

*Holotype*: male “Papua New Guinea: Eastern Highlands, Akameku - Brahmin, Bismarck Range, 2200m, 23.xi.2006, 05.56.801S 145.22.238E, Balke & Kinibel (PNG 106)” (ZSM). *Paratypes*: **Eastern Highlands:** 15 males, 12 females with the same label as the holotype (NHMW, ZSM). 9 males, 11 females “Papua New Guinea: Eastern Highlands, Akameku - Brahmin, Bismarck Range, 2400m, 23.xi.2006, 05.55.615S 145.22.699E, Balke & Kinibel (PNG 107)” (NHMW, ZSM). 8 males, 3 females “Papua New Guinea: Eastern Highlands, Goroka, Mt. Gahavisuka, 2200m, 8.iv.2006, 06.00.896S 145.24.753E, Balke & Sagata (PNG 35)” (NHMW, ZSM). 9 males “Papua New Guinea: Eastern Highlands, Goroka, Daulo Pass, 2500m, 19.v.2006, 06.02.432S 145.13.333E, John & Balke (PNG 67)”, one male additionally with a green label “DNA M.Balke 1306” (NHMW, ZSM). 1 male “Papua New Guinea: Eastern Highlands, 37 km S Goroka, Hogave vill., Mt. Michael, 2179-2800m, 9.-15.vii.2009, 06.22.479S 145.15.256E, Sagata (PNG 230)” (ZSM). **Simbu:** 2 males, 1 female “Papua New Guinea Simbu prov L. Cizek lgt.”, “Kundiawa, Mu vill. 145°02'E 4°42'S [6°05'S; 145°02'E] III.2001, 1900m” (ZSM).

#### Additional material.

**Eastern Highlands:** 2 males “Papua New Guinea: Eastern Highlands, Aiyura, 1670m, 5.iv.2006, 06.21.131S 145.54.398E, Balke & Sagata (PNG 32)” (ZSM). 1 male “Papua New Guinea: Eastern Highlands, Aiyura, creek, 1670 m, 20.v.2006, 06.21.131S 145.54.398E, John & Balke (PNG 70)”, “DNA M.Balke 1310” [green] (ZSM). 2 males “Papua New Guinea: Eastern Highlands, Onerunka, small creek, red soil /rock, 1700m, 21.v.2006, 06.20.936S 145.46.874E, John & Balke (PNG 71)”, one male additionally with a green label “DNA M.Balke 1304” (ZSM). 6 males “Papua New Guinea: Eastern Highlands, Kimiagomo vill, north Okapa stn, 1900, 30.iv.2006, 06.25.407S 145.34.480E, Sagata (PNG 80)” (NHMW, ZSM). 2 males “Papua New Guinea: Eastern Highlands, Wapi Creek, Kimiagomo, Okapa,, 1900m, 9.viii.2005, 6 25.407S 145 34.480E, K.Sagata (WB122)” (ZSM). 1 male “Papua New Guinea: EHP, Okapa, Kimiagomo, Wapi Creek, 6.25.407 / 145.34.480, 1900m, 9.viii.2005, Sagata, DNA MB1252” (ZSM). 5 males, 2 females “Papua New Guinea: Eastern Highlands, Yuyulio, Kimiagomo-Okapa, 2100m, 13.iv.2003, 06 25.255S 145 34.233E, K. Sagata (WB7)” (NHMW, ZSM). 1 male, 1 female “Papua New Guinea: Eastern Highlands, Tegupate creek Kimiagomo, Okapa, 1900m, 9.viii.2005, 6 25.407S 145 34.480E, K.Sagata (WB124)” (ZSM). 2 males, 1 female “Papua New Guinea: Eastern Highlands, Marawaka, Ande, 1700m, 8.xi.2005, 07.01.697S 145.49.807E, Balke & Kinibel (PNG 86)” (ZSM). **Gulf:** 5 males, 1 female “Papua New Guinea: Gulf, Marawaka, Andakombe towards Morobe, 2160m, 12.xi.2006, 07.11.717S 145.51.177E, Balke & Kinibel (PNG 94)”, one male and one female additionally with labels “DNA M.Balke 1369” and “DNA M.Balke 1371” respectively (NHMW, ZSM). These specimens are not included in the type series because most of them are teneral and some of them are slightly different from the types in body shape, surface sculpture, and shape of the median lobe. At present, it is impossible to postulate whether they belong to *Exocelina bismarckensis* sp. n. or one or two additional species; for that more material is requite from the region (Fig. 53).

#### Diagnosis.

Beetle medium-sized, dark brown to piceous, with paler clypeus, vertex, and pronotal sides, submatt to matt; pronotum with distinct lateral bead; male antennomeres 3–5 evidently enlarged, with margins more or less rounded, almost equal in size, antennomeres 6 and 7 somewhat enlarged; male protarsomere 4 with medium-sized, slender, evidently curved anterolateral hook-like seta; median lobe with weak submedian constriction, distal part broadened, and apex almost rounded in ventral view and thin, curved, and pointed in lateral view; paramere with shallow notch on dorsal side and subdistal part elongate, with numerous, dense, more or less long, thin setae. The species is similar to *Exocelina gorokaensis* sp. n., from which differs with duller dorsal surface due to denser punctation and stronger microreticulation, as well as apex of the median lobe rounded in ventral view and narrower in lateral view and paramere with shallow, not distinct notch on dorsal side. From *Exocelina vovae* sp. n., the species differs with more elongate habitus and apex of the median lobe almost rounded, not distinctly concave in ventral view.

#### Description.

*Size and shape*: Beetle medium-sized (TL-H 3.5–4.2 mm, TL 3.9–4.6 mm, MW 1.8–2.2 mm), with oblong habitus, broadest at elytral middle, some specimens with subparallel elytral sides. *Coloration*: Dorsal surface more or less uniform dark brown to piceous, paler on clypeus, vertex, pronotal sides, and along elytral suture; head appendages and legs yellowish red to dark reddish, legs darker distally (Fig. 43). Teneral specimens paler.

*Surface sculpture*: Head with very dense, coarse punctation (spaces between punctures 1–2 times size of punctures). Pronotum with punctation finer than on head. Elytra with punctation sparser than on pronotum. Pronotum and elytra with weaker or stronger impressed microreticulation, dorsal surface submatt to matt. Head with microreticulation stronger. Metaventrite and metacoxa distinctly microreticulate, metacoxal figs with longitudinal strioles and transverse wrinkles. Abdominal ventrites with distinct microreticulation, strioles, and fine sparse punctation, coarser and denser on two last abdominal ventrites.

*Structures*: Pronotum with distinct lateral bead. Base of prosternum and neck of prosternal process and neck of prosternal process with distinct ridge, smooth and not rounded anteriorly, with small anterolateral extensions. Blade of prosternal process lanceolate, relatively narrow, convex, with distinct lateral bead and few setae; neck and blade of prosternal process evenly jointed. Abdominal ventrite 6 slightly truncate or concave apically.

*Male*: Antennomeres 3–5 evidently enlarged, almost equal in size, with margins more or less rounded, antennomeres 6 and 7 somewhat enlarged (Fig. 15A), antennomeres 3–7 rugose ventrally. Protarsomere 4 with medium-sized, slender, evidently curved anterolateral hook. Protarsomere 5 ventrally with anterior row of 21 elongate setae and posterior row of 8 shorter setae (Fig. 15B). Abdominal ventrite 6 with 10–13 lateral striae on each side, slightly truncate or concave apically. Median lobe with weak submedian constriction, distal part broadened, and apex more or less rounded in ventral view and thin, curved, and pointed in lateral view, with upper margin sinuate or almost straight (Fig, 15C, D). Paramere with shallow notch on dorsal side and subdistal part elongate, with numerous, dense, more or less long, thin setae (Fig. 15E).

*Holotype*: TL-H 3.9 mm, TL 4.25 mm, MW 2.05 mm.

*Female*: Antennae simple, abdominal ventrite 6 broadly rounded apically, without striae.

#### Variability.

The species shows intra- and interpopulational variability in coloration, body shape, microreticulation, and shape of median lobe and abdominal ventrite 6.

#### Distribution.

Papua New Guinea: Eastern Highlands and Simbu Provinces. The species is known mainly from Bismarck Range (Fig. 53).

#### Etymology.

The species is named after Bismarck Range. The name is an adjective in the nominative singular.

### 3. Exocelina craterensis

Taxon classificationAnimaliaColeopteraDytiscidae

Shaverdo & Balke sp. n.

http://zoobank.org/9ABA3B0B-78FD-4871-96BA-8E993C3480C9

Figs 4 , 32

#### Type locality.

Papua New Guinea: Simbu/Eastern Highlands Provinces, Crater Mt., Wara Sera Station, 06°43.4'S; 145°05.6'E.

#### Type material.

*Holotype*: male “Papua New Guinea, Simbu/EHPr. Crater Mountain, Wara Sera Station, 820 m, 14IX2002, Balke & Sagata (PNG 8)” (ZSM). *Paratypes*: **Simbu/Eastern Highlands:** 2 males with the same label as the holotype (NHMW, ZSM). 2 males “Papua New Guinea: Simbu / EHP, Crater Mountain, Sera – Herowana, Wara Pima, 900m, 15IX2002, Balke & Sagata, (PNG 011)”, one of them additionally with a label “DNA M.Balke 6182” (ZSM). **Gulf:** 2 males, 2 females “Papua New Guinea: Gulf Province, Marawaka, Mala, 1400m, 11.xi.2006, 07.05.664S 145.44.467E, Balke & Kinibel, (PNG 90)”, “DNA M.Balke 6183” (NHMW, ZSM).

#### Diagnosis.

Beetle small, piceous, with dark brown head and sides of pronotum; pronotum with lateral bead; male antennomeres simple; male protarsomere 4 with large, thick, strongly curved anterolateral hook-like seta; median lobe with submedian constriction in ventral view and strongly elongate apex in lateral view; paramere with notch on dorsal side and subdistal part elongate, with brush of long, dense, thin setae. The species is similar to *Exocelina oceai* Shaverdo, Hendrich & Balke, 2012, from which differs with darker dorsal coloration and structure of the male genitalia.

#### Description.

*Size and shape*: Beetle small (TL-H 3.05–3.3 mm, TL 3.4–3.65 mm, MW 1.6–1.8 mm), with oblong-oval habitus, broadest at elytral middle. *Coloration*: Head dark brown, with reddish clypeus; pronotum piceous, with paler sides, reddish at anterior angles; elytra piceous, sometimes with reddish brown sutural lines; head appendages yellowish brown, legs darker distally (Fig. 32).

*Surface sculpture*: Head with relatively sparse punctation (spaces between punctures 1–4 times size of punctures), evidently finer and sparser anteriorly; diameter of punctures smaller than diameter of cells of microreticulation. Pronotum with extremely sparse and fine punctation, almost invisible. Elytra without punctation, only with several extremely fine punctures and with punctural rows. Pronotum and elytra with weakly impressed microreticulation, dorsal surface shiny. Head with microreticulation stronger. Metaventrite and metacoxa distinctly microreticulate, metacoxal figs with longitudinal strioles and transverse wrinkles. Abdominal ventrites with distinct microreticulation, strioles, and extremely fine, sparse punctation, almost invisible, only slightly coarser and denser on two last abdominal ventrites.

*Structures*: Pronotum with lateral bead. Base of prosternum and neck of prosternal process with distinct ridge, less smooth and slightly rounded anteriorly, with anterolateral extensions. Ridge laterally with distinct punctation. Blade of prosternal process lanceolate, relatively broad, convex, with distinct lateral bead and few setae; neck and blade of prosternal process evenly jointed. Abdominal ventrite 6 slightly concave apically.

*Male*: Antenna simple (Fig. 4A). Protarsomere 4 with large, thick, strongly curved anterolateral hook-like seta. Protarsomere 5 ventrally with anterior row of 12 and posterior row of 5 short setae (Fig. 4B). Abdominal ventrite 6 with 6–8 lateral striae on each side. Median lobe with submedian constriction in ventral view and strongly elongate apex in lateral view (Fig. 4C, D). Paramere with notch on dorsal side and subdistal part elongate, with brush of long, dense, thin setae (Fig. 4E).

*Holotype*: TL-H 3.05 mm, TL 3.4 mm, MW 1.6 mm.

*Female*: Without evident differences in external morphology from male, except for abdominal ventrite 6 without striae.

#### Distribution.

Papua New Guinea: Simbu/Eastern Highlands and Gulf Provinces (Fig. 53).

#### Etymology.

The species is named after Crater Mountain where it was collected. The name is an adjective in the nominative singular.

### Exocelina edeltraudae

Taxon classificationAnimaliaColeopteraDytiscidae

Shaverdo, Hendrich & Balke, 2012

Figs 8 , 37

#### Type locality.

Papua New Guinea: Western Highlands Province, Kurumul, 6 km SW Kudjip, 05°53.43'S; 144°36.60'E.

#### Type material.

*Holotype*: male “Papua New Guinea: Western Highlands, Kurumul, 6Km SW Kudjip, small stream, 1584m, 13.vi.1994, 05.53.426S 144.36.600E, John (PNG 78)” (ZSM). *Paratypes*: 11 males with the same label as the holotype, one of them additionally with a green label “DNA M.Balke 1341” (NHMW, ZSM). 2 males “Papua New Guinea: Western Highlands, Mt. Hagen town area, 1600m, 7.xii.1994 05.49.745S 144.22.357E Balke & Kinibel (PNG 131)” (ZSM).

#### Additional material.

2 males “Papua New Guinea: Western Highlands, Kurumul, 6Km SW Kudjip, small stream, 1584m, 13.vi.1994, 05.53.426S 144.36.600E, John (PNG 78)” (ZSM). 6 males “Papua New Guinea: Western Highlands, Gonzsidai-Sarup, 1700m, 4.iii.2007, 05.19.060S 144.28.671E, Kinibel (PNG 144)” (NHMW, ZSM). 26 females “Papua New Guinea: Western Highlands, Gonzsidai-Sarup, 1700m, 4.iii.2007, 05.19.060S 144.28.671E, Kinibel (PNG 144)” (ZSM), these females are most likely a mixture of two species: *Exocelina edeltraudae* and a species from the *Exocelina broschii*-group. 142 females with the same label as the holotype (ZSM), these females are most likely a mixture of three species: *Exocelina edeltraudae* and two species from the *Exocelina broschii*- and *Exocelina rivulus*-groups. 30 females “Papua New Guinea: Western Highlands, Mt. Hagen town area, 1600m, 7.xii.1994 05.49.745S 144.22.357E Balke & Kinibel (PNG 131)” (ZSM), these females are most likely a mixture of two species: *Exocelina edeltraudae* and a species from the *Exocelina broschii*-group.

#### Diagnosis.

Beetle medium-sized, piceous, slightly submatt; pronotum with distinct lateral bead; male ventrite 6 slightly to distinctly concave apically; male antennomeres 3–5 distinctly enlarged, almost equal in size and shape, antennomeres 6–8 enlarged; male protarsomere 4 with medium-sized, slender, evidently curved anterolateral hook-like seta; median lobe with very strong submedian constriction and proximal part very broad in ventral view, apex of median lobe pointed and strongly curved downwards in lateral; paramere with distinct notch on dorsal side and subdistal part elongate, with numerous, dense, long, thin setae. The species is very similar to *Exocelina pseudoedeltraudae* sp. n., from which differs with slightly shinier dorsal surface, due to weaker punctation and microreticulation, with smaller and less rounded male antennomeres 3–5 (for male antennomeres 3 and 4, ratio width/length: < 0.92) and apex of median lobe broader in lateral view.

#### Redescription.

*Size and shape*: Beetle medium-sized (TL-H 3.5–3.85 mm, TL 3.9–4.3 mm, MW 1.9–2.05 mm), with oblong-oval habitus, broadest at elytral middle. *Coloration*: Dorsally piceous, with dark brown anterior margin of head and narrowly pronotal sides; head appendages and legs reddish to reddish brown, legs distally darker (Fig. 37). Teneral specimens paler.

*Surface sculpture*: Head with dense, coarse punctation (spaces between punctures 1–3 times size of punctures). Pronotum with punctation finer, sparser, and more evenly distributed than on head. Elytra with punctation much finer, sparser than on pronotum. Pronotum and elytra with less strongly impressed microreticulation, dorsal surface slightly submatt. Head with microreticulation stronger. Metaventrite and metacoxa distinctly microreticulate, metacoxal figs with longitudinal strioles and transverse wrinkles. Abdominal ventrites with distinct microreticulation, long strioles, and fine sparse punctation, coarser and denser on two last abdominal ventrites.

*Structures*: Pronotum with distinct lateral bead. Base of prosternum and neck of prosternal process with distinct ridge, rounded and smooth anteriorly, with small anterolateral extensions. Blade of prosternal process lanceolate, relatively narrow, convex, with distinct lateral bead and few setae; neck and blade of prosternal process evenly jointed. Abdominal ventrite 6 broadly rounded, or slightly truncate, or concave apically.

*Male*: Antennomeres 3–5 distinctly enlarged, almost equal in size, antennomeres 6–8 enlarged (Fig. 8A), antennomeres 3–7 rugose ventrally. Protarsomere 4 with medium-sized, slender, evidently curved anterolateral hook-like seta. Protarsomere 5 ventrally with anterior row of 16 and posterior row of 5 short setae (Fig. 8B). Abdominal ventrite 6 with 8–13 lateral striae on each side, slightly to distinctly concave apically. Median lobe with very strong submedian constriction and proximal part very broad in ventral view, apex of median lobe pointed and strongly curved downwards in lateral view (Fig. 8D, E). Paramere with distinct notch on dorsal side and subdistal part elongate, with numerous, dense, long, thin setae (Fig. 8F).

*Holotype*: TL-H 3.85 mm, TL 4.3 mm, MW 2.05 mm.

*Female*: Antennae simple, abdominal ventrite 6 broadly rounded or slightly truncate apically, without striae.

#### Distribution.

Papua New Guinea. The species is known only from Western Highlands Province (Fig. 53).

### 4. Exocelina gorokaensis

Taxon classificationAnimaliaColeopteraDytiscidae

Shaverdo & Balke sp. n.

http://zoobank.org/C87417FC-F7D6-4395-9897-02628EB0DF59

Figs 14 , 42

Exocelina undescribed sp. MB1307: Toussaint et al. 2014: Supplementary figs 1–4, Tab. 2.

#### Type locality.

Papua New Guinea: Eastern Highlands Province, 37 km S Goroka, Hogave village, Mt. Michael, 06°22.48'S; 145°15.26'E.

#### Type material.

*Holotype*: male “Papua New Guinea: Eastern Highlands, 37 km S Goroka, Hogave vill., Mt. Michael, 2179-2800m, 9.-15vii.2009, 06.22.479S 145.15.256E, Sagata (PNG 230)” (ZSM). *Paratypes*: **Eastern Highlands:** 23 males, 39 females with the same label as the holotype, 1 male additionally with a green label “DNA M.Balke 4038” (NHMW, ZSM). 5 males, 4 females “Papua New Guinea: Eastern Highlands, Goroka, Mt. Gahavisuka, 2200m, 8.iv.2006, 06.00.896S 145.24.753E, Balke & Sagata (PNG 35)” (NHMW, ZSM). 1 male, 1 female “Papua New Guinea: Eastern Highlands, Hogu, Mt. Barola, 1900m, 9.v.2006, 06.17.556S 145.45.036E, Balke & Sagata (PNG 56)” (ZSM). 10 males, 5 females “Papua New Guinea: Eastern Highlands, Goroka, below Mt. Otto, 2000m, 11.v.2006, 06.01.687S 145.26.493E, Balke (PNG 57)”, one male additionally with a green label “DNA M.Balke 1305” (NHMW, ZSM). 21 males, 14 females “Papua New Guinea: Eastern Highlands, Goroka, Daulo Pass, 2500m, 19.v.2006, 06.02.432S 145.13.333E, John & Balke (PNG 67)”, one male additionally with a green label “DNA M.Balke 1307” (NHMW, ZSM). 2 males, 1 female “Papua New Guinea: Eastern Highlands, Akameku - Brahmin, Bismarck Range, 2200m, 23.xi.2006, 05.56.801S 145.22.238E, Balke & Kinibel (PNG 106)” (ZSM). 1 female “Papua New Guinea: Eastern Highlands, Akameku - Brahmin, Bismarck Range, 2400m, 23.xi.2006, 05.55.615S 145.22.699E, Balke & Kinibel (PNG 107)”, “DNA M.Balke 1518” [green] (ZSM). **Simbu:** 8 males, 5 females “Papua New Guinea Simbu prov L. Cizek lgt.”, “Kundiawa, Mu vill. 145°02'E 4°42'S [6°05'S; 145°02'E] III.2001, 1900m” (ZSM). **Western Highlands:** 9 males, 4 females “Papua New Guinea: Western Highlands, Mondmill,5 Km SE Minj, small pools near creek, 1741m, 12.vi.2006, 05.56.801S 144.39.898E, John (PNG 77)”, one male additionally with a green label “DNA M.Balke 1343” (NHMW, ZSM).

#### Diagnosis.

Beetle medium-sized, dark brown to piceous, with paler clypeus, vertex, and pronotal sides, submatt; pronotum with distinct lateral bead; male antennomeres 3–5 evidently enlarged, slightly rounded, almost equal in size, antennomeres 6 and 7 somewhat enlarged; male protarsomere 4 with medium-sized, slender, evidently curved anterolateral hook-like seta; median lobe with very weak submedian constriction and apex very slightly concave in ventral view and with apex slightly pointed and broadened in lateral view; paramere with distinct notch on dorsal side and subdistal part elongate, with numerous, dense, more or less long, thin setae. The species is similar to *Exocelina jimiensis* sp. n., from which differs with evident punctation of the dorsal surface and shape of the median lobe. Also it is similar to *Exocelina vovai* sp. n. and *Exocelina bismarckensis* sp. n., from which differs with weaker punctation of the dorsal surface, paramere with distinct notch on the dorsal side, and shape of the median lobe.

#### Description.

*Size and shape*: Beetle medium-sized (TL-H 3.7–4.3 mm, TL 4.1–4.8 mm, MW 1.95–2.4 mm), with oblong-oval habitus, broadest at elytral middle. *Coloration*: Dorsal surface more or less uniform dark brown to piceous, paler on clypeus, vertex, pronotal sides, and along elytral suture; head appendages and legs yellowish red to dark reddish, legs darker distally (Fig. 42). Teneral specimens paler.

*Surface sculpture*: Head with very dense, coarse punctation (spaces between punctures 1–2 times size of punctures). Pronotum with punctation finer than on head. Elytra with punctation sparser than on pronotum. Pronotum and elytra with relatively strongly impressed microreticulation, dorsal surface submatt. Head with microreticulation stronger. Metaventrite and metacoxa distinctly microreticulate, metacoxal figs with longitudinal strioles and transverse wrinkles. Abdominal ventrites with distinct microreticulation, strioles, and fine sparse punctation, coarser and denser on two last abdominal ventrites.

*Structures*: Pronotum with distinct lateral bead. Base of prosternum and neck of prosternal process and neck of prosternal process with distinct ridge, smooth and not rounded anteriorly, with small anterolateral extensions. Blade of prosternal process lanceolate, relatively narrow, convex, with distinct lateral bead and few setae; neck and blade of prosternal process evenly jointed. Abdominal ventrite 6 broadly rounded or slightly truncate apically.

*Male*: Antennomeres 3–5 evidently enlarged, slightly rounded, almost equal in size, antennomeres 6 and 7 somewhat enlarged (Fig. 14A), antennomeres 3–7 rugose ventrally. Protarsomere 4 with medium-sized, slender, evidently curved anterolateral hook. Protarsomere 5 ventrally with anterior row of 17 and posterior row of 6 short setae (Fig. 14B). Abdominal ventrite 6 with 11–14 lateral striae on each side, broadly rounded or slightly truncate apically. Median lobe with very weak submedian constriction and apex very slightly concave in ventral view and with apex slightly pointed and broadened in lateral view (Fig. 14C, D). Paramere with distinct notch on dorsal side and subdistal part elongate, with numerous, dense, more or less long, thin setae (Fig. 14E).

*Holotype*: TL-H 4.0 mm, TL 4.5 mm, MW 2.15 mm.

*Female*: Antennae simple, abdominal ventrite 6 broadly rounded apically, without striae.

#### Distribution.

Papua New Guinea: Eastern Highlands, Simbu, and Western Highlands Provinces. The species is known mainly from the area around Goroka and Kundiawa (Fig. 53).

#### Etymology.

The species is named after Goroka, where it was mostly collected. The name is an adjective in the nominative singular.

### 5. Exocelina herowana

Taxon classificationAnimaliaColeopteraDytiscidae

Shaverdo & Balke sp. n.

http://zoobank.org/840FD36C-53AD-4688-BB49-4E3ADE52C677

Figs 5 , 33

#### Type locality.

Papua New Guinea: Simbu/Eastern Highlands Provinces, Crater Mt., Sera – Herowana, upper Oh River, approximately 06°43.4'S; 145°05.6'E.

#### Type material.

*Holotype*: male “Papua New Guinea: Crater Mountain, Sera – Herowana, upper Oh river, 1200m, 15IX2002, Balke & Sagata, (PNG 012)”, “DNA M.Balke 6181” (ZSM).

#### Diagnosis.

Beetle small, dark brown, with slightly paler head and pronotum, shiny; pronotum with lateral bead; male antennomeres 3–9 stout, with 4–5 slightly larger than other antennomeres; male protarsomere 4 with large slender, evidently anterolateral hook-like seta; median lobe with very strong median constriction and proximal part very broad in ventral view, apex of median lobe broad, slightly concave in middle and twisted at both sides in ventral view and shortly pointed in lateral view; paramere with distinct notch on dorsal side and subdistal part elongate, with a large brush of long, dense, thin setae; proximal setae almost invisible. The species is similar to *Exocelina edeltraudae* and *Exocelina pseudoedeltraudae* sp. n., from which differs with smaller size and stout, not evidently modified, male antennomeres.

#### Description.

*Size and shape*: Beetle small (TL-H 3.6 mm, TL 4.0 mm, MW 2.0 mm), with oblong-oval habitus, broadest at elytral middle. *Coloration*: Head and pronotum reddish-brown, pronotal disc brown; elytra dark brown; head appendages yellowish-brown, legs reddish-brown, darker distally (Fig. 33).

*Surface sculpture*: Head with dense, coarse punctation (spaces between punctures 1–3 times size of punctures). Pronotum with punctation much finer, sparser, and more evenly distributed than on head. Elytra with punctation much finer, sparser than on pronotum, almost invisible. Pronotum and elytra with less strongly impressed microreticulation, dorsal surface shiny. Head with microreticulation stronger. Metaventrite and metacoxa distinctly microreticulate, metacoxal figs with longitudinal strioles and transverse wrinkles. Abdominal ventrites with distinct microreticulation, long strioles, and fine sparse punctation, coarser and denser on two last abdominal ventrites.

*Structures*: Pronotum with lateral bead. Base of prosternum and neck of prosternal process with distinct ridge, smooth and slightly rounded anteriorly, without anterolateral extensions. Blade of prosternal process lanceolate, relatively narrow, convex, with distinct lateral bead and few setae; neck and blade of prosternal process evenly jointed. Abdominal ventrite 6 broadly rounded.

*Male*: Antennomeres 3–9 stout, with 4–5 slightly larger than other antennomeres (Fig. 5A). Protarsomere 4 with large, slender, evidently curved anterolateral hook-like seta. Protarsomere 5 ventrally with anterior row of 12 setae and posterior row of 4 short setae (Fig. 5B). Abdominal ventrite 6 with 8–10 lateral striae on each side. Median lobe with very strong median constriction and proximal part very broad in ventral view, apex of median lobe broad, slightly concave in middle and twisted at both sides in ventral view and shortly pointed in lateral view (Fig. 5C, D). Paramere with distinct notch on dorsal side and subdistal part elongate, with a large brush of long, dense, thin setae; proximal setae almost invisible (Fig. 5E).

*Female*: Unknown.

#### Distribution.

Papua New Guinea: Simbu/Eastern Highlands Provinces. This species is known only from the type locality (Fig. 53).

#### Etymology.

The species is named after the type locality. The name is a noun in the nominative singular standing in apposition.

### 6. Exocelina jimiensis

Taxon classificationAnimaliaColeopteraDytiscidae

Shaverdo & Balke sp. n.

http://zoobank.org/D2D78386-9C7F-4839-8C96-5A46FB47268F

Figs 10 , 36

Exocelina undescribed sp. MB3311: Toussaint et al. 2014: Supplementary figs 1–4, Tab. 2.

#### Type locality.

Papua New Guinea: Western Highlands Province, Kundum, 05°16.10'S; 144°27.87'E.

#### Type material.

*Holotype*: male “Papua New Guinea: Western Highlands, Kundum, 1400m, 3.iii.2007, 05.16.096S 144.27.869E, Kinibel (PNG 142)” (ZSM). *Paratypes*: 64 males with the same labels as the holotype (NHMW, ZSM). 10 males “Papua New Guinea: Western Highlands, Simbai, Kairong River, 1850m, 2.iii.2007, 05.14.840S 144.28.457E, Kinibel (PNG 139)” (NHMW, ZSM). 20 males “Papua New Guinea: Western Highlands, Simbai - Jimi, 1500m, 2.iii.2007, 05.16.074S 144.27.886E, Kinibel (PNG 140)”, one male additionally with a green label “DNA M.Balke 3311” (NHMW, ZSM). 2 males “Papua New Guinea: Western Highlands, Jimi, 1500m, 2.iii.2007, 05.16.335S 144.27.930E, Kinibel (PNG 141)” (ZSM). 8 males, 4 females “Papua New Guinea: Western Highlands, Jimi Valley, above Sendiap Station, 2000m, 6.iii.2007, 05.19.314S 144.31.266E, Kinibel (PNG 148)” (NHMW, ZSM). 7 males, 1 female “Papua New Guinea: Western Highlands, Simbai area, 2200m, 6.iii.2007, 05.18.752S 144.31.849E, Kinibel (PNG 149)” (NHMW, ZSM). 3 males, 3 females “Papua New Guinea: Western Highlands, Simbai area, 2500m, 8.iii.2007, 05.14.202S 144.33.651E, Kinibel (PNG 150)” (NHMW, ZSM).

#### Additional material.

33 females with the same labels as the holotype (ZSM). 38 females “Papua New Guinea: Western Highlands, Simbai, Kairong River, 1850m, 2.iii.2007, 05.14.840S 144.28.457E, Kinibel (PNG 139)” (ZSM). 10 females “Papua New Guinea: Western Highlands, Simbai - Jimi, 1500m, 2.iii.2007, 05.16.074S 144.27.886E, Kinibel (PNG 140)” (ZSM). 7 females “Papua New Guinea: Western Highlands, Jimi, 1500m, 2.iii.2007, 05.16.335S 144.27.930E, Kinibel (PNG 141)” (ZSM). These females might belong to two species: *Exocelina jimiensis* sp. n. and a species from the *Exocelina broschii*-group.

#### Diagnosis.

Beetle medium-sized, dark brown to piceous, with paler clypeus, vertex, and pronotal sides, slightly submatt; pronotum with distinct lateral bead; male antennomeres 3–5 evidently enlarged, slightly rounded, almost equal in size, external margin of antennomere 5 almost straight, antennomere 6 somewhat enlarged; male protarsomere 4 with large, slender, evidently curved anterolateral hook-like seta; median lobe with submedian constriction and apex bluntly pointed, broadened in lateral view; paramere with distinct notch on dorsal side and subdistal part elongate, with numerous, dense, more or less long, thin setae. The species is similar to *Exocelina sandaunensis* sp. n. and *Exocelina simbaiarea* sp. n., from which differs with stronger punctation on pronotum, male antennomeres 3–5 smaller and more equal in size, median lobe with stronger submedian constriction in ventral view and more broadened apex in lateral view, subdistal part of paramere with setae more numerous, shorter, and thinner.

#### Description.

*Size and shape*: Beetle medium-sized (TL-H 3.8–4.0 mm, TL 4.15–4.4 mm, MW 2.0–2.15 mm), with oblong-oval habitus, broadest at elytral middle. *Coloration*: Dorsal surface more or less uniform dark brown to piceous, paler on clypeus, vertex, pronotal sides, and along elytral suture; head appendages and legs yellowish red to dark reddish, legs darker distally (Fig. 36). Teneral specimens paler.

*Surface sculpture*: Head with dense, coarse punctation (spaces between punctures 1–3 times size of punctures). Pronotum with punctation finer than on head. Elytra with punctation finer, sparser than on pronotum. Pronotum and elytra with relatively weakly impressed microreticulation, dorsal surface slightly submatt. Head with microreticulation stronger. Metaventrite and metacoxa distinctly microreticulate, metacoxal figs with longitudinal strioles and transverse wrinkles. Abdominal ventrites with distinct microreticulation, strioles, and fine sparse punctation, coarser and denser on two last abdominal ventrites.

*Structures*: Pronotum with distinct lateral bead. Base of prosternum and neck of prosternal process and neck of prosternal process with distinct ridge, smooth and not rounded anteriorly, with small anterolateral extensions. Blade of prosternal process lanceolate, relatively narrow, convex, with distinct lateral bead and few setae; neck and blade of prosternal process evenly jointed. Abdominal ventrite 6 broadly rounded or slightly truncate apically.

*Male*: Antennomeres 3–5 evidently enlarged, slightly rounded, almost equal in size, external margin of antennomere 5 almost straight, antennomere 6 somewhat enlarged (Fig. 10A), antennomeres 3–6 rugose ventrally. Protarsomere 4 with large, slender, evidently curved anterolateral hook. Protarsomere 5 ventrally with anterior row of 13 and posterior row of 4 short setae (Fig. 10B). Abdominal ventrite 6 with 6–12 lateral striae on each side, slightly truncate apically. Median lobe with submedian constriction and apex bluntly pointed, broadened in lateral view (Fig. 10C, D). Paramere with distinct notch on dorsal side and subdistal part elongate, with numerous, dense, more or less long, thin setae (Fig. 10E).

*Holotype*: TL-H 3.9 mm, TL 4.25 mm, MW 2.15 mm.

*Female*: Antennae simple, abdominal ventrite 6 broadly rounded apically, without striae.

#### Distribution.

Papua New Guinea: Western Highlands Province. The species is known only from the area of Jimi River (Fig. 53).

#### Etymology.

The species is named after Jimi River, in the area in which it was collected. The name is an adjective in the nominative singular.

### 7. Exocelina kisli

Taxon classificationAnimaliaColeopteraDytiscidae

Shaverdo & Balke sp. n.

http://zoobank.org/5A7BAF2D-2881-4DA7-9D3D-BE2FBA32709E

Figs 17 , 45

Exocelina undescribed sp. MB1373: Toussaint et al. 2014: Supplementary figs 1–4, Tab. 2.

#### Type locality.

Papua New Guinea: Morobe Province, Menyamya, Mt. Inji, ca. 07°14.81'S; 146°01.33'E.

#### Type material.

*Holotype*: male “Papua New Guinea: Morobe, Menyamya, Mt Inji, 1900m, 14.XI.2006, nr 07.14.813S 146.01.330E, Balke & Kinibel, (PNG 97)” (ZSM). *Paratypes*: **Morobe:** 2 males, 1 female with the same label as the holotype (NHMW, ZSM). **Gulf:** 1 male, 1 female “Papua New Guinea: Gulf, Menyamya, Mt Inji, 1700m, 14.xi.2006, nr 07.14.813S 146.01.330E, Balke & Kinibel, (PNG 96)” (ZSM). 2 males, 1 female “Papua New Guinea: Gulf, 1500m, 13.xi.2006, 07.11.721S 145.54.746E, Balke & Kinibel, (PNG 95)” (NHMW, ZSM), one male and the female additionally with green labels “DNA M.Balke 1373” and “DNA M.Balke 4243”, respectively.

#### Diagnosis.

Beetle medium-sized, piceous, with dark brown head and pronotum; pronotum with lateral bead; male antennomere 3 evidently larger than other antennomeres; male protarsomere 4 with very small (smaller than more laterally situated large seta), thin, slightly curved anterolateral hook-like seta; median lobe with evident submedian constriction, apex of median lobe almost rounded in lateral view; paramere without notch on dorsal side, with relatively long and dense subdistal setae and spine-like setae on internal surface. The species is similar to *Exocelina knoepfchen* and *Exocelina ksionseki* sp. n. It differs from *Exocelina knoepfchen* with dorsal surface matt due to stronger punctation and microreticulation, male antennomeres 3–5 larger, and median lobe slender; from *Exocelina ksionseki* sp. n. with larger size, dorsal surface matt due to stronger microreticulation, male antennomeres 3 smaller and more triangular, male protarsomere 4 with anterolateral hook-like seta smaller than more laterally situated large seta, apex of median lobe more rounded in lateral view, paramere only slightly longer than medial lobe, with less numerous subdistal setae and spine-like setae, and abdominal ventrite 6 less striated.

#### Description.

*Size and shape*: Beetle medium-sized (TL-H 4.3–4.5 mm, TL 4.7–4.9 mm, MW 2.35–2.5 mm), with elongate habitus, broadest at elytral middle. *Coloration*: Head dark brown, sometimes with reddish clypeus and vertex; pronotum dark brown, sometimes with piceous disc and/or with reddish sides; elytra uniformly piceous or with reddish brown sutural lines; head appendages yellowish or reddish, legs usually darker distally (Fig. 45). Teneral specimens paler: yellowish red head and pronotum and brown elytra.

*Surface sculpture*: Head with very dense, coarse punctation (spaces between punctures 1–2 times size of punctures), finer and sparser anteriorly; diameter of punctures only slightly smaller than diameter of cells of microreticulation, of some punctures equal to it. Pronotum and elytra with slightly finer and more evenly distributed punctation than on head. Head, pronotum, and elytra with strongly impressed microreticulation. Dorsal surface matt due to strong punctation and microreticulation. Metaventrite and metacoxa distinctly microreticulate, metacoxal figs with longitudinal strioles and transverse wrinkles. Abdominal ventrites with distinct microreticulation, strioles, and fine sparse punctation, coarser and denser on two last abdominal ventrites.

*Structures*: Pronotum with lateral bead. Base of prosternum and neck of prosternal process with distinct ridge, anteriorly with weak transverse lines and less rounded, without anterolateral extensions. Blade of prosternal process lanceolate, relatively narrow, convex, with distinct lateral bead and few setae; neck and blade of prosternal process evenly jointed. Abdominal ventrite 6 broadly rounded or slightly truncate apically.

*Male*: Antennomere 2 very small, stout, antennomere 3 strongly enlarged, evidently larger than other antennomeres, more triangular, antennomeres 4 and 5 distinctly enlarged, antennomeres 6 and 7 slightly enlarged (Fig. 17A). Protarsomere 4 with very small (smaller than more laterally situated large seta), thin, slightly curved anterolateral hook-like seta. Protarsomere 5 ventrally with anterior row (double apically) of 20 short setae and posterior row of 6 short setae (Fig. 17B). Abdominal ventrite 6 with 7–9 lateral striae on each side, slightly truncate apically. Median lobe with evident submedian constriction in ventral view and slightly rounded apex in lateral view (Figs 17C, D). Paramere slightly longer than median lobe, without notch on dorsal side, with relatively long and dense subdistal setae, short and sparse proximal setae, and spine-like setae on internal surface (Fig. 17E).

*Holotype*: TL-H 4.5 mm, TL 4.9 mm, MW 2.4 mm.

*Female*: Antennomere 1 as in male or only slightly larger, other antennomeres simple, abdominal ventrite 6 broadly rounded apically, without striae.

#### Distribution.

Papua New Guinea: Morobe and Gulf Provinces (Fig. 53).

#### Etymology.

The species is named for F. Kisl. The species name is a noun in the genitive case.

### 8. Exocelina ksionseki

Taxon classificationAnimaliaColeopteraDytiscidae

Shaverdo & Balke sp. n.

http://zoobank.org/515088A3-CFC0-4743-B2D6-F8D8E38E2787

Figs 18 , 46

#### Type locality.

Papua New Guinea: Madang Province, Adalbert Mts., near Keki, 04°43.06'S; 145°24.44'E.

#### Type material.

*Holotype*: male “Papua New Guinea: Madang, Keki, Adalbert Mts, 400m, 29.xi.2006, 04.43.058S 145.24.437E, Binatang Boys, (PNG 119)” (ZSM). *Paratypes*: **Madang:** 1 male with the same label as the holotype (NHMW). **Western Highlands:** 2 males, 1 female “Papua New Guinea: Western Highlands, Kurumul, 6Km SW Kudjip, small stream, 1580m, 13.vi.2006, 05.53.426S 144.36.600E, John (PNG 78)” (ZSM). 4 males, 2 females “Papua New Guinea: Western Highlands, Mt. Hagen town area, 1600m, 7.xii.2006 05.49.745S 144.22.357E Balke & Kinibel (PNG 131)” (NHMW, ZSM).

#### Diagnosis.

Beetle medium-sized, piceous, with reddish sides of pronotum and sometimes with reddish head, submatt; pronotum with lateral bead; male antennomere 3 evidently larger than other antennomeres; male protarsomere 4 with very small (only slightly larger than more laterally situated large seta), thin, slightly curved anterolateral hook-like seta; median lobe with very weak submedian constriction, apex of median lobe elongate in lateral view; paramere distinctly longer than median lobe, without notch on dorsal side, with relatively long and dense subdistal setae and numerous spine-like setae on internal surface, proximal setae almost absent. The species is similar to *Exocelina knoepfchen* Shaverdo, Hendrich & Balke, 2012 except for evidently smaller size, coarse, dense dorsal punctation, beetle submatt, male antennomeres 3 larger, with more rounded external margin, male protarsomere 4 with anterolateral hook-like seta larger than more laterally situated large seta, narrow apical half (in ventral view) of median lobe, with elongate apex in lateral view, and setation of paramere. It differs from *Exocelina kisli* sp. n., see under *Exocelina kisli* sp. n.

#### Description.

*Size and shape*: Beetle medium-sized (TL-H 3.8–4.2 mm, TL 4.2–4.6 mm, MW 2–2.3 mm), with elongate habitus, broadest at elytral middle. *Coloration*: Head reddish to piceous with reddish clypeus; pronotum piceous, with reddish sides; elytra uniformly piceous or with reddish brown sutural lines; head appendages yellowish or reddish, legs usually darker distally (Fig. 46). Teneral specimens paler.

*Surface sculpture*: Head with very dense, coarse punctation (spaces between punctures 1–2 times size of punctures), finer and sparser anteriorly; diameter of punctures only slightly smaller than diameter of cells of microreticulation, of some punctures equal to it. Pronotum and elytra with slightly finer and more evenly distributed punctation than on head. Pronotum and elytra with more weakly impressed microreticulation than on head. Dorsal surface submatt due to strong punctation. Metaventrite and metacoxa distinctly microreticulate, metacoxal figs with longitudinal strioles and transverse wrinkles. Abdominal ventrites with distinct microreticulation, strioles, and fine sparse punctation, coarser and denser on two last abdominal ventrites.

*Structures*: Pronotum with lateral bead. Base of prosternum and neck of prosternal process with distinct ridge, smooth and rounded anteriorly, without anterolateral extensions. Blade of prosternal process lanceolate, relatively narrow, convex, with distinct lateral bead and few setae; neck and blade of prosternal process evenly jointed. Abdominal ventrite 6 broadly rounded apically.

*Male*: Antennomere 2 very small, stout, antennomere 3 strongly enlarged, evidently larger than other antennomeres, with rounded external margin, antennomeres 4–6 distinctly enlarged, antennomere 7 slightly enlarged (Fig. 18A). Protarsomere 4 with very small (only slightly larger than laterally situated large seta), thin, slightly curved anterolateral hook-like seta. Protarsomere 5 ventrally with anterior row (double apically) of 14 short setae and posterior row of 4 short setae (Fig. 18B). Abdominal ventrite 6 with 14–17 lateral striae on each side. Median lobe narrow in apical half and broad in basal one, with weak submedian constriction in ventral view and elongate apex in lateral view (Fig. 18C, D). Paramere distinctly longer than median lobe, without notch on dorsal side, with relatively long and dense subdistal setae and numerous spine-like setae on internal surface (Fig. 18E).

*Holotype*: TL-H 4.1 mm, TL 4.5 mm, MW 2.15 mm.

*Female*: Antennomere 1 as in male or only slightly larger, other antennomeres simple, abdominal ventrite 6 broadly rounded apically, without striae.

#### Distribution.

Papua New Guinea: Madang and Western Highlands Provinces (Fig. 53).

#### Etymology.

The species is named for K. Ksionsek. The species name is a noun in the genitive case.

### 9. Exocelina lembena

Taxon classificationAnimaliaColeopteraDytiscidae

Shaverdo & Balke sp. n.

http://zoobank.org/C2F6AFB9-769E-4389-8A5F-5A3403D1DA9A

Figs 26 , 51

Exocelina undescribed sp. MB4922: Toussaint et al. 2014: Supplementary figs 1–4, Tab. 2.

#### Type locality.

Papua New Guinea: East Sepik Province, Lembena, 04°57.33'S; 143°57.30'E.

#### Type material.

*Holotype*: male “Papua New Guinea: East Sepik, Lembena, 297m, 8.ix.2009, 04 57.329S 143 57.297E, Ibalim & Pius (PNG247)” (ZSM). *Paratypes*: 10 males, 9 females with the same label as the holotype, two males additionally with labels “DNA M. Balke 4922”, “DNA M. Balke 4923” (NHMW, ZSM). 1 male “Papua New Guinea: East Sepik, Lembena, 335m, 10.ix.2009, 04 56.859S 143 59.375E, Ibalim & Pius (PNG250)”, “DNA M. Balke 4919” (ZSM). 1 male “Papua New Guinea: East Sepik, Lembena, 335m, 10.ix.2009, 04 56.859S 143 57.379E, Ibalim & Pius (PNG251)” (ZSM). 1 male, 1 female “Papua New Guinea: East Sepik, Lembena, 335m, 10.ix.2009, 04 56.921S 143 57.478E, Ibalim & Pius (PNG252)” (ZSM). 1 female “Papua New Guinea: East Sepik, Lembena, 117m, 8.ix.2009, 04 57.513S 143 57.296E, Ibalim & Pius (PNG248)” (ZSM).

#### Diagnosis.

Beetle small, dark brown to piceous, with paler head or only its anterior part and pronotal sides, shiny; pronotum without lateral bead or with weak traces of lateral bead; male antennomeres simple; male protarsomere 4 with large, thick, evidently curved anterolateral hook-like seta; median lobe with strong submedian constriction in ventral view and almost truncate apex in lateral view; paramere with strong notch on dorsal side and subdistal part elongate but broad, with numerous long, dense, thick, flattened setae, some of them curved at apex; setae of proximal part evident, long. The species is very similar to *Exocelina brahminensis* Shaverdo, Hendrich & Balke, 2012, from which differs with shape of the median lobe apex and, especially, with setation of the subdistal part of the paramere: it has only thick, flattened setae. From *Exocelina mantembu* Shaverdo, Hendrich & Balke, 2012, it differs with shape of the median lobe and stronger notch on dorsal side of the paramere.

#### Description.

*Size and shape*: Beetle small (TL-H 2.95–3.45 mm, TL 3.3–4.5 mm, MW 1.6–1.9 mm), with oblong-oval habitus, broadest at elytral middle. *Coloration*: Head dark brown to piceous, paler anteriorly; pronotum dark brown to piceous, with red to reddish brown sides; elytra uniformly dark brown to piceous; head appendages red to reddish brown, legs darker, especially metathoracic legs (Fig. 51).

*Surface sculpture*: Head with dense (spaces between punctures 1–3 times size of punctures) but fine punctation; diameter of punctures evidently smaller than diameter of cells of microreticulation. Pronotum with much sparser and finer punctation than on head. Elytra with extremely sparse and fine punctation, almost invisible. Pronotum and elytra with weakly impressed microreticulation, dorsal surface shiny. Head with microreticulation stronger. Metaventrite and metacoxa distinctly microreticulate, metacoxal figs with longitudinal strioles and transverse wrinkles. Abdominal ventrites with distinct microreticulation, strioles, and fine sparse punctation, coarser and denser on two last abdominal ventrites.

*Structures*: Pronotum without lateral bead or with weak traces of lateral bead. Base of prosternum and neck of prosternal process with distinct ridge, smooth and rounded anteriorly, with small anterolateral extensions. Blade of prosternal process lanceolate, relatively narrow, slightly convex, with distinct lateral bead and few setae; neck and blade of prosternal process evenly jointed. Abdominal ventrite 6 broadly rounded apically.

*Male*: Antenna simple (Fig. 26A). Protarsomere 4 with large, thick, evidently curved anterolateral hook-like seta. Protarsomere 5 ventrally with anterior row of 11 and posterior row of 4 short setae (Fig. 26B). Abdominal ventrite 6 with 6–8 lateral striae on each side. Median lobe with strong submedian constriction in ventral view and almost truncate apex in lateral view (Fig. 26C, D). Paramere with strong notch on dorsal side and subdistal part elongate but broad, with numerous long, dense, thick, somewhat flattened setae, some of them curved at apex; setae of proximal part evident, long (Fig. 26E).

*Holotype*: TL-H 3.45 mm, TL 3.8 mm, MW 1.85 mm.

*Female*: Without evident differences in external morphology from males, except for abdominal ventrite 6 without striae.

#### Distribution.

Papua New Guinea: East Sepik Province. This species is known only from localities near Lembena (Fig. 53).

#### Etymology.

The name refers to the village of Lembena where this species was collected. The name is a noun in the nominative singular standing in apposition.

### 10. Exocelina mantembu

Taxon classificationAnimaliaColeopteraDytiscidae

Shaverdo & Balke sp. n.

http://zoobank.org/8EB2CC12-C97D-4259-9519-687F68626ED2

Figs 25 , 50

Exocelina undescribed sp. MB0060: Toussaint et al. 2014: Supplementary figs 1–4, Tab. 2.

#### Type locality.

Indonesia: Papua Province: Yapen Islands Regency, Mantembu, near Serui, approximately 01°50'S; 136°14'E.

#### Type material.

*Holotype*: male “Irian Java: Japen [sic!] Isl. Mantembu 150–450 m, 18.2.1992 leg. Riedel” (NHMW). *Paratypes*: 8 males, 4 female with the same label as the holotype (MZB, NHMW, ZSM). 6 males, 6 females “Indonesia: Papua, Japen [sic!] Mantembu A. Riedel” (ZSM). 1 male “59 DNA M Balke” [green], “Mantembu” [hw] (ZSM). 1 male “60 DNA M Balke” [green], “Mantembu” [hw] (ZSM).

#### Diagnosis.

Beetle small, dark brown, with paler head and pronotum, shiny; pronotum without lateral bead; male antennomeres simple; male protarsomere 4 with large, thick, strongly curved anterolateral hook-like seta; median lobe slender, with very weak submedian constriction in ventral view and broadly rounded, elongate apex in lateral view; paramere with shallow notch on dorsal side and subdistal part elongate but broad, with large brush of long, dense, thick, somewhat flattened setae, distal ones longer and curved at apex; setae of proximal part short, thin, almost invisible. The species is similar to *Exocelina lembena* sp. n., from which differs with shape of the median lobe apex and shallow notch on dorsal side of the paramere. There are only two species on Yapen Island, which can be easily distinguished with size and dorsal sculpture: *Exocelina mantembu* sp. n., small and shiny, and *Exocelina vladimiri*, large and matt, as well as using shape and structure of the male genitalia.

#### Description.

*Size and shape*: Beetle small (TL-H 3.15–3.45 mm, TL 3.50–3.85 mm, MW 1.65–1.85 mm), with oblong-oval habitus, broadest at elytral middle, some specimens narrower towards elytral apex. *Coloration*: Head reddish brown, darker posterior eyes; pronotum reddish brown, with paler sides; elytra uniformly dark brown; head appendages reddish brown, legs darker, especially metathoracic legs (Fig. 50).

*Surface sculpture*: Head with dense punctation (spaces between punctures 1–3 times size of punctures), evidently finer and sparser anteriorly; diameter of punctures smaller than diameter of cells of microreticulation. Pronotum with much sparser and finer punctation than on head. Elytra with extremely sparse and fine punctation, almost invisible. Pronotum and elytra with weakly impressed microreticulation, dorsal surface shiny. Head with microreticulation stronger. Metaventrite and metacoxa distinctly microreticulate, metacoxal figs with longitudinal strioles and transverse wrinkles. Abdominal ventrites with distinct microreticulation, strioles, and fine sparse punctation, coarser and denser on two last abdominal ventrites.

*Structures*: Pronotum without lateral bead. Base of prosternum and neck of prosternal process with distinct ridge, less rounded anteriorly, without anterolateral extensions. Blade of prosternal process lanceolate, relatively broad, convex, with distinct lateral bead and few setae; neck and blade of prosternal process evenly jointed. Abdominal ventrite 6 broadly rounded or slightly truncate apically.

*Male*: Antenna simple (Fig. 25A). Protarsomere 4 with large, thick, strongly curved anterolateral hook-like seta. Protarsomere 5 ventrally with anterior row of 11 short setae and posterior row of 4 short setae (Fig. 25B). Abdominal ventrite 6 with 8–11 lateral striae on each side. Median lobe slender, with very weak submedian constriction in ventral view and broadly rounded, elongate apex in lateral view (Fig. 25C, D). Paramere with shallow notch on dorsal side and subdistal part elongate but broad, with large brush of long, dense, relatively thick, somewhat flattened setae, distal ones longer and curved at apex; setae of proximal part short, thin, almost invisible (Fig. 25E).

*Holotype*: TL-H 3.45 mm, TL 3.85 mm, MW 1.85 mm.

*Female*: Without evident differences in external morphology from males, except for abdominal ventrite 6 without striae.

#### Distribution.

Indonesia: Papua Province: Yapen Islands Regency. This species is known only from the type locality (Fig. 53).

#### Etymology.

The name refers to the region Mantembu where this species was collected. The name is a noun in the nominative singular standing in apposition.

### 11. Exocelina michaelensis

Taxon classificationAnimaliaColeopteraDytiscidae

Shaverdo & Balke sp. n.

http://zoobank.org/68743C87-57E6-44AF-9C62-5D7746D1BAD4

Figs 2 , 31

#### Type locality.

Papua New Guinea: Eastern Highlands Province, 37 km S Goroka, Hogave village, Mt. Michael, 06°22.48'S; 145°15.26'E.

#### Type material.

*Holotype*: male “Papua New Guinea: Eastern Highlands, 37 km S Goroka, Hogave vill., Mt. Michael, 2179-2800m, 9.-15vii.2009, 06.22.479S 145.15.256E, Sagata (PNG 230)”, “DNA M. Balke 4082” [green] (ZSM).

#### Diagnosis.

Beetle medium-sized, piceous, with dark brown clypeus and sides of pronotum; pronotum with lateral bead; male antennomeres simple; male protarsomere 4 with large, thin, slightly curved anterolateral hook-like seta; median lobe with submedian constriction in ventral view and elongate and broadly pointed apex in lateral view; paramere with distinct notch on dorsal side and subdistal part elongate, with brush of long, dense, thin setae. Size, dorsal sculpture, and structure of the male genitalia of this species strongly resemble those of *Exocelina bismarckensis* sp. n., *Exocelina gorokaensis* sp. n., and *Exocelina vovai* sp. n., but the species can be easily distinguished from them with its simple, not modified, male antenna.

#### Description.

*Size and shape*: Beetle medium-sized (TL-H 3.85 mm, TL 4.3 mm, MW 2.1 mm), with oblong-oval habitus, broadest at elytral middle. *Coloration*: Dorsal surface uniform piceous, with dark brown clypeus and sides of pronotum; head appendages yellowish brown, legs darker distally (Fig. 31).

*Surface sculpture*: Head with relatively sparse punctation (spaces between punctures 1–4 times size of punctures); diameter of punctures smaller than diameter of cells of microreticulation. Pronotum and elytra with distinct punctation but finer and more evenly distributed than on head. Pronotum and elytra with relatively strongly impressed microreticulation, dorsal surface submatt. Head with microreticulation stronger. Metaventrite and metacoxa distinctly microreticulate, metacoxal figs with longitudinal strioles and transverse wrinkles. Abdominal ventrites with distinct microreticulation, strioles, and extremely fine, sparse punctation, almost invisible, only slightly coarser and denser on two last abdominal ventrites.

*Structures*: Pronotum with lateral bead. Base of prosternum and neck of prosternal process with distinct ridge, smooth and rounded anteriorly, without anterolateral extensions. Blade of prosternal process lanceolate, relatively narrow, convex, with distinct lateral bead and few setae; neck and blade of prosternal process evenly jointed. Abdominal ventrite 6 broadly rounded.

*Male*: Antenna simple (Fig. 31). Protarsomere 4 with large, thin, slightly curved anterolateral hook-like seta. Protarsomere 5 ventrally with anterior row of more than 40 and posterior row of 13 thin, moderately long setae (Fig. 2A). Abdominal ventrite 6 with 15–18 lateral striae on each side. Median lobe with submedian constriction in ventral view and elongate and broadly pointed apex in lateral view (Figs 2B, C). Paramere with distinct notch on dorsal side and subdistal part elongate, with brush of long, dense, thin setae (Fig. 2D).

*Female*: Unknown.

#### Distribution.

Papua New Guinea: Eastern Highlands Province. This species is known only from the type locality (Fig. 53).

#### Etymology.

The species is named after Mt. Michael where it was collected. The name is an adjective in the nominative singular.

### 12. Exocelina pinocchio

Taxon classificationAnimaliaColeopteraDytiscidae

Shaverdo & Balke sp. n.

http://zoobank.org/2258B993-3A90-4F48-AFC5-F85FC6002C9E

Figs 24 , 48

Exocelina undescribed sp. MB3321: Toussaint et al. 2014: Supplementary figs 1–4, Tab. 2.

#### Type locality.

Papua New Guinea: Madang Province, Usino, 05°31.13'S; 145°25.32'E.

#### Type material.

*Holotype*: male “Papua New Guinea: Madang, Usino, 260m, 15.iii.2007, 05.31.125S 145.25.316E, Kinibel (PNG 158)” (ZSM). *Paratype*: 8 males with the same label as the holotype, one of them additionally with green label “DNA M.Balke 3321” (NHMW, ZSM).

#### Additional material.

26 females with the same label as the holotype (NHMW, ZMS). These females might belong to of two species: *Exocelina pinocchio* sp. n. and *Exocelina brahminensis*, therefore, they are not included in the type series.

#### Diagnosis.

Beetle small, dark brown to piceous, with paler head and pronotal sides, shiny; pronotum without lateral bead; male antennomeres simple; male protarsomere 4 with large, thick, strongly curved anterolateral hook-like seta; in ventral view, median lobe with strong submedian constriction and ventral sclerite apically divided in three parts and in lateral view, with very strongly protruding apex, forming long thin prolongation; paramere with strong notch on dorsal side and subdistal part elongate but broad, with numerous long, dense setae, thinner and shorter distally and thicker, longer, and curved at apex proximally. This species can be easily distinguished from all small, shiny, with simple antennae, and without pronotal bead species (e.g., *Exocelina brahminensis* or *Exocelina lembena* sp. n.) with shape of its median lobe.

#### Description.

*Size and shape*: Beetle small (TL-H 3.15–3.45 mm, TL 3.50–3.8 mm, MW 1.64–1.87 mm), with oblong-oval habitus, broadest at elytral middle. *Coloration*: Head dark brown, paler anteriorly; pronotum dark brown, with reddish brown sides and in some specimens also paler disc; elytra uniformly dark brown to piceous; head appendages red to reddish brown (in teneral specimens yellow to yellowish-red), legs darker, especially metathoracic legs (Fig. 48).

*Surface sculpture*: Head with dense (spaces between punctures 1–3 times size of punctures) but fine punctation; diameter of punctures evidently smaller than diameter of cells of microreticulation. Pronotum with much sparser and finer punctation than on head. Elytra with extremely sparse and fine punctation, almost invisible. Pronotum and elytra with weakly impressed microreticulation, dorsal surface shiny. Head with microreticulation stronger. Metaventrite and metacoxa distinctly microreticulate, metacoxal figs with longitudinal strioles and transverse wrinkles. Abdominal ventrites with distinct microreticulation, strioles, and fine sparse punctation, coarser and denser on two last abdominal ventrites.

*Structures*: Pronotum without lateral bead. Base of prosternum and neck of prosternal process with distinct ridge, smooth and rounded anteriorly, without anterolateral extensions. Blade of prosternal process lanceolate, relatively broad, slightly convex, with distinct lateral bead and few setae; neck and blade of prosternal process evenly jointed. Abdominal ventrite 6 broadly rounded apically.

*Male*: Antenna simple (Fig. 24A). Protarsomere 4 with large, thick, strongly curved anterolateral hook-like seta. Protarsomere 5 ventrally with anterior row of 12 and posterior row of 4 short setae (Fig. 24B). Abdominal ventrite 6 with 8–10 lateral striae on each side. Median lobe with strong submedian constriction and ventral sclerite apically divided in three parts in ventral view and with very strongly protruding apex, forming long thin prolongation in lateral view (Fig. 24C, D). Paramere with strong notch on dorsal side and subdistal part elongate but broad, with numerous long, dense setae, thinner and shorter distally and thicker, longer, and curved at apex proximally (Fig. 24E).

*Holotype*: TL-H 3.4 mm, TL 3.8 mm, MW 1.8 mm.

*Female*: Without evident differences in external morphology from males, except for abdominal ventrite 6 without striae.

#### Distribution.

Papua New Guinea: Madang Province. This species is known only from the type locality (Fig. 53).

#### Etymology.

The species is named for a fictional character from the book “The Adventures of Pinocchio” by Carlo Collodi because the apex of its median lobe has a prolongation like a “nose”. The name is a noun in the nominative singular standing in apposition.

### 13. Exocelina pseudoastrophallus

Taxon classificationAnimaliaColeopteraDytiscidae

Shaverdo & Balke sp. n.

http://zoobank.org/70A22773-B02C-4E21-B297-1AB45131579B

Figs 3A–D , 30

#### Type locality.

Papua New Guinea: East Sepik Province, Lembena, 04°57.51'S; 143°57.03'E.

#### Type material.

*Holotype*: male “Papua New Guinea: East Sepik, Lembena, 117m, 8.ix.2009, 04 57.513S 143 57.296E, Ibalim & Pius (PNG248)” (ZSM). *Paratypes*: 2 females with the same label as the holotype (NHMW, ZSM). 1 male “Papua New Guinea: East Sepik, Lembena, 297m, 8.ix.2009, 04 57.329S 143 57.297E, Ibalim & Pius (PNG247)”, “DNA M.Balke 6184” (ZSM).

#### Diagnosis.

Beetle middle-sized, piceous, with paler pronotum (especially on margins) and head, dorsally with evident punctation, submatt; pronotum with distinct lateral bead; male antennomeres simple; male protarsomere 4 with large, thick, strongly curved anterolateral hook-like seta; median lobe short and with extremely strongly discontinuous (broken and curved) outline; paramere with shallow notch on dorsal side and subdistal part elongate, with dense, long, thin setae. The species is very similar to *Exocelina astrophallus* (Balke, 1998), except for media lobe without notch on left side and larger and more strongly curved anterolateral hook-like seta.

#### Description.

*Size and shape*: Beetle middle-sized (TL-H 3.65–3.7 mm, TL 3.95–4.1 mm, MW 2.0–2.05 mm), with oblong-oval habitus, broadest at elytral middle, with elytral apex slightly rounded. *Coloration*: as in *Exocelina astrophallus* (Fig. 30).

*Surface sculpture*: as in *Exocelina astrophallus*.

*Structures*: Pronotum with distinct lateral bead. Base of prosternum and neck of prosternal process with distinct ridge, without anterolateral extensions. Blade of prosternal process lanceolate, rather narrow, strongly convex, with distinct bead and few setae; neck and blade of prosternal process evenly jointed.

*Male*: Antenna simple (Fig. 30). Protarsomere 4 with large, thick, strongly curved anterolateral hook-like seta. Protarsomere 5 ventrally with anterior row of 31 setae and posterior row of 8 relatively long setae (Fig. 3A). Abdominal ventrite 6 with 13–19 long lateral striae on each side. Median lobe short and with extremely strongly discontinuous (curved, plicate) outline, without notch on left side (Fig. 3B, D). Paramere with shallow notch on dorsal side and subdistal part elongate, with dense, long, thin setae (Fig. 3C).

*Holotype*: TL-H 3.7 mm, TL 4.1 mm, MW 2.05 mm.

*Female*: Without evident differences in external morphology from male, except for abdominal ventrite 6 without striae.

#### Distribution.

Papua New Guinea: East Sepik Province. This species is known only from localities near Lembena (Fig. 53).

#### Etymology.

This species was mistaken for *Exocelina astrophallus* due to their external similarity. The name is a noun in the nominative singular standing in apposition.

### 14. Exocelina pseudobifida

Taxon classificationAnimaliaColeopteraDytiscidae

Shaverdo & Balke sp. n.

http://zoobank.org/876C2734-EB9F-4924-986E-5361DF37A0FB

Figs 19 , 47

Exocelina undescribed sp. MB0659: Toussaint et al. 2014: supplementary figs 1–4, Tab. 2.

#### Type locality.

Papua New Guinea: Sandaun Province, Mekil, 04°48.74'S; 141°39.08'E.

#### Type material.

*Holotype*: male “Papua New Guinea: Sandaun, MekilK [!], 1718m, 14.x.2003, 4 48.742S 141 39.075E, K. Sagata (WB106)” (ZSM). *Paratypes*: 6 females with the same label as the holotype (NHMW, ZSM). 1 male “Papua New Guinea: Sandaun: Mekil (WB106), 14.x.2003, K. Sagata, DNA M Balke: MB 659”, “DNA M. Balke 659” (ZSM).

#### Diagnosis.

Beetle small, dark brown to piceous, shiny; pronotum without lateral bead; male antennomeres simple; male protarsomere 4 with large, thick, strongly curved anterolateral hook; median lobe with strong submedian constriction and apex bifid: with small dorsal extension; paramere with notch on dorsal side and subdistal part elongate, with dense, long, thin setae. The species is very similar to *Exocelina bifida* Shaverdo, Hendrich & Balke, 2012, except for structure of genitalia: apical lobes slender and more deeply separated, dorsal extension prominent but not deeply cut.

#### Description.

*Size and shape*: Beetle small (TL-H 3.3–3.7 mm, TL 3.75–4.15 mm, MW 1.75–2.0 mm), with oblong-oval habitus, broadest at elytral middle. *Coloration*: as in *Exocelina bifida* (Fig. 47).

*Surface sculpture*: Punctation and microreticulation as in *Exocelina bifida*.

*Structures*: Pronotum without lateral bead. Base of prosternum and neck of prosternal process with distinct ridge, anteriorly less rounded, smooth, with small anterolateral extensions. Blade of prosternal process lanceolate, relatively broad, slightly convex, with distinct lateral bead and few setae; neck and blade of prosternal process evenly jointed. Abdominal ventrite 6 broadly rounded apically.

*Male*: Antenna simple (Fig. 19A). Protarsomere 4 with large, thick, strongly curved anterolateral hook. Protarsomere 5 ventrally with anterior row of 15 and posterior row of 3 short setae (Fig. 19B). Abdominal ventrite 6 with 6–8 lateral striae on each side. Median lobe with strong submedian constriction and apex bifid: with small dorsal extension (Fig. 19C, D). Paramere with notch on dorsal side and subdistal part elongate, with dense, long, thin setae (Fig. 19E).

*Holotype*: TL-H 3.7 mm, TL 4.15 mm, MW 2.0 mm.

*Female*: Without evident differences in external morphology from male, except for abdominal ventrite 6 without striae.

#### Distribution.

Papua New Guinea: Sandaun Province, Mekil. This species is known only from the type locality (Fig. 53).

#### Etymology.

This species was mistaken for *Exocelina bifida* due to their similarity. The name is a noun in the nominative singular standing in apposition.

### 15. Exocelina pseudoedeltraudae

Taxon classificationAnimaliaColeopteraDytiscidae

Shaverdo & Balke sp. n.

http://zoobank.org/C7536736-ABCE-42EC-89D0-678762B728AE

Figs 9 , 38 ; 4A–F, 30 in Shaverdo et al. (2012)

Exocelina undescribed sp. MB1288: Toussaint et al. 2014: Supplementary figs 1–4, Tab. 2.

#### Type locality.

Papua New Guinea: Hela Province, Koroba, 05°41.85'S; 142°43.836'E.

#### Type material.

*Holotype*: male “Papua New Guinea: Southern Highlands, Koroba, 1600m, 15.v.1994, 05.41.854S 142.43.836E, Balke (PNG 66)”, “Paratypus *Exocelina edeltraudae* sp. n. des. H.Shaverdo, L.Hendrich & M.Balke, 2012” (ZSM). *Paratypes*: 5 males, 1 female with the same labels as the holotype (NHMW, ZSM). 3 males “PAPUA N.G.: 6.–9.5.1998 Southern Highl. Prov. Tari-Koroba, Hedemari [Hedamali] 1700-1900 m, leg. Riedel”, “Paratypus *Exocelina edeltraudae* sp. n. des. H.Shaverdo, L.Hendrich & M.Balke, 2012” (NHMW). 1 male, 3 females “Papua New Guinea: Southern Highlands, Tari Komo road, 10km N Hides Gas, 1700m, 13.v.1994, Balke (PNG 61)”, “Paratypus *Exocelina edeltraudae* sp. n. des. H.Shaverdo, L.Hendrich & M.Balke, 2012”, the male additionally with a green label “DNA M.Balke 1288” (ZSM). 5 males, 8 females “Papua New Guinea: Southern Highlands, Tari to Koroba, 1600m, 15.v.1994, 05.46.500S 142.50.000E, Balke (PNG 65)”, “Paratypus *Exocelina edeltraudae* sp. n. des. H.Shaverdo, L.Hendrich & M.Balke, 2012” (NARI, NHMW, ZSM). 1 female “Papua New Guinea: Southern Highlands, Tari to Koroba, 1600m, 15.v.1994, 05.46.500S 142.50.000E, Balke (PNG 65)” (ZSM).

#### Diagnosis.

Beetle medium-sized, piceous, submatt; pronotum with distinct lateral bead; male ventrite 6 slightly to distinctly concave apically; male antennomeres 3–5 distinctly enlarged, almost equal in size and shape, antennomeres 6–8 enlarged; male protarsomere 4 with large, slender, evidently curved anterolateral hook-like seta; median lobe with very strong submedian constriction and proximal part very broad in ventral view, apex of median lobe pointed and strongly curved downwards in lateral; paramere with distinct notch on dorsal side and subdistal part elongate, with numerous, dense, long, thin setae. The species is very similar to *Exocelina edeltraudae* Shaverdo, Hendrich & Balke, 2012, from which differs with less shinier dorsal surface, due to stronger punctation and microreticulation, with larger and more rounded male antennomeres 3–5 (for male antennomeres 3 and 4, ratio width/length: > 1.0) and apex of median lobe narrower in lateral view.

#### Description.

*Size and shape*: Beetle medium-sized (TL-H 3.45–4.0 mm, TL 3.85–4.45 mm, MW 1.8–2.2 mm), with oblong-oval habitus, broadest at elytral middle. *Coloration*: Dorsally piceous, with dark brown anterior margin of head and narrowly pronotal sides; head appendages and legs reddish to reddish brown, legs distally darker (Fig. 38). Teneral specimens paler.

*Surface sculpture*: Head with dense, coarse punctation (spaces between punctures 1–3 times size of punctures), especially on vertex. Pronotum with punctation finer, sparser, and more evenly distributed than on head. Elytra with punctation finer, sparser than on pronotum. Pronotum and elytra with strongly impressed microreticulation, dorsal surface submatt. Head with microreticulation stronger. Metaventrite and metacoxa distinctly microreticulate, metacoxal figs with longitudinal strioles and transverse wrinkles. Abdominal ventrites with distinct microreticulation, strioles, and fine sparse punctation, coarser and denser on two last abdominal ventrites.

*Structures*: Pronotum with distinct lateral bead. Base of prosternum and neck of prosternal process with distinct ridge, rounded and smooth anteriorly, with small anterolateral extensions. Blade of prosternal process lanceolate, relatively narrow, convex, with distinct lateral bead and few setae; neck and blade of prosternal process evenly jointed. Abdominal ventrite 6 broadly rounded, or slightly truncate, or concave apically.

*Male*: Antennomeres 3–5 distinctly enlarged, almost equal in size, antennomeres 6–8 enlarged (Fig. 9A), antennomeres 3–7 rugose ventrally. Protarsomere 4 with large, slender, evidently curved anterolateral hook. Protarsomere 5 ventrally with anterior row of 15 and posterior row of 5 short setae (Fig. 9B). Abdominal ventrite 6 with 8–13 lateral striae on each side, slightly to distinctly concave apically (Fig. 9C). Median lobe with very strong submedian constriction and proximal part very broad in ventral view, apex of median lobe pointed and strongly curved downwards in lateral view (Fig. 9D, E). Paramere with distinct notch on dorsal side and subdistal part elongate, with numerous, dense, long, thin setae (Fig. 9F).

*Holotype*: TL-H 4.0 mm, TL 4.45 mm, MW 2.2 mm.

*Female*: Antennae simple, abdominal ventrite 6 broadly rounded or slightly truncate apically, without striae.

#### Distribution.

Papua New Guinea. The species is known only from Hela Province (Fig. 53).

#### Etymology.

In an earlier work (Shaverdo et al. 2012), this species was mistaken for *Exocelina edeltraudae*. The name is a noun in the nominative singular standing in apposition.

### 16. Exocelina pseudoeme

Taxon classificationAnimaliaColeopteraDytiscidae

Shaverdo & Balke sp. n.

http://zoobank.org/CEAA3D42-6D93-43B8-BAAC-1B4A8CB7C742

Figs 27 , 52

Exocelina undescribed sp. MB3759: Toussaint et al. 2014: Supplementary figs 1–4, Tab. 2.

#### Type locality.

Papua New Guinea: Sandaun Province, Mianmin, 04°53.42'S; 141°37.03'E.

#### Type material.

*Holotype*: male “Papua New Guinea: Sandaun, Mianminold [!], 898m, 20.x.2003, 4 53.419S 141 37.028E, K. Sagata (WB66)” (ZSM). *Paratypes*: 1 male, 1 female “Papua New Guinea: Sandaun, Mianmin (pool), 700m, 21.x.2008, 04.52.858S 141.31.706E, Ibalim (PNG 198)” and with two green labels “DNA M.Balke 3747”, “DNA M.Balke 3759” respectively (ZSM).

#### Diagnosis.

Beetle small, dark brown to piceous, with paler anterior part of head and pronotal sides, shiny; pronotum without lateral bead; male antennomeres 5–10 slightly stout; male protarsomere 4 with large, slender, strongly curved anterolateral hook-like seta; median lobe with submedian constriction in ventral view and elongate apex in lateral view; paramere with notch on dorsal side and subdistal part elongate, with large brush of two kinds of setae: upper setae thin and less numerous and lower setae long, thick, somewhat flattened, and curved at apex; setae of proximal part shorter, thinner, less evident. The species is very similar to *Exocelina eme* Shaverdo, Hendrich & Balke, 2012 except for more weakly impressed dorsal microreticulation, especially on pronotum, beetle dorsally slightly shinier, as well as for structure and setation of genitalia: median lobe with stronger submedian constriction and symmetrical apex in ventral view; subdistal part of paramere with upper thin setae less numerous making brush smaller.

#### Description.

*Size and shape*: Beetle small (TL-H 3.15–3.55 mm, TL 3.5–4.0 mm, MW 1.65–1.85 mm), with oblong-oval habitus, broadest at elytral middle. *Coloration*: Head dark brown to piceous, paler anteriorly; pronotum dark brown to piceous, with red to reddish brown sides; elytra uniformly dark brown to piceous; head appendages red to reddish brown, legs darker, especially metathoracic legs (Fig. 52).

*Surface sculpture*: Head with dense punctation (spaces between punctures 1–3 times size of punctures), finer and sparser anteriorly; diameter of punctures smaller than diameter of cells of microreticulation. Pronotum with much finer and sparser punctation than on head. Elytra with very sparse and fine punctation, almost invisible. Head, pronotum, and elytra with weakly impressed microreticulation, dorsal surface shiny. Head with microreticulation stronger. Metaventrite and metacoxa distinctly microreticulate, metacoxal figs with longitudinal strioles and transverse wrinkles. Abdominal ventrites with distinct microreticulation, strioles, and fine sparse punctation, coarser and denser on two last abdominal ventrites.

*Structures*: Pronotum without lateral bead. Base of prosternum and neck of prosternal process with distinct ridge, smooth and less rounded anteriorly, with small anterolateral extensions. Blade of prosternal process lanceolate, relatively narrow, slightly convex, with distinct lateral bead and few setae; neck and blade of prosternal process evenly jointed. Abdominal ventrite 6 broadly rounded apically.

*Male*: Antennomeres 5–10 slightly stout (Fig. 27A). Protarsomere 4 with large, slender, strongly curved anterolateral hook-like seta. Protarsomere 5 ventrally with anterior row of 14 and posterior row of 5 short setae (Fig. 27B). Abdominal ventrite 6 with 5–6 lateral striae on each side. Median lobe with submedian constriction and symmetrical apex in ventral view and elongate apex in lateral view (Figs 27C, D); paramere with notch on dorsal side and subdistal part elongate, with large brush of two kinds of setae: upper setae thin and less numerous and lower setae long, thick, somewhat flattened, and curved at apex; setae of proximal part shorter, thinner, less evident (Fig. 27E).

*Holotype*: TL-H 3.55 mm, TL 4 mm, MW 1.85 mm.

*Female*: Without evident differences in external morphology from males, except for abdominal ventrite 6 without striae.

#### Distribution.

Papua New Guinea: Sandaun Province. This species is known only from Mianmin region (Fig. 53).

#### Etymology.

This species was mistaken for *Exocelina eme* due to their external similarity. The name is a noun in the nominative singular standing in apposition.

### 17. Exocelina sandaunensis

Taxon classificationAnimaliaColeopteraDytiscidae

Shaverdo & Balke sp. n.

http://zoobank.org/0133AA56-7AA3-413B-B58D-89FE64CCF29E

Figs 13 , 41

#### Type locality.

Papua New Guinea: Sandaun Province, Sokamin, 04°50.85'S; 141°37.87'E.

#### Type material.

*Holotype*: male “Papua New Guinea: Sandaun, Sokamin4, 1200m, 19.x.2003, 4 50.845S 141 37.865E, K. Sagata (WB 102)” (ZSM). *Paratypes*: 4 males with the same label as the holotype, one male additionally with a label “DNA M. Balke 666” (NHMW, ZSM). 3 males, 4 females “Papua New Guinea: Sandaun, Sokamin4, 1200m, 19.x.2003, 4 50.845S 141 37.865E, K. Sagata (WB 100)”, one male additionally with a label “DNA M. Balke 682” (NHMW, ZSM). 1 male “Papua New Guinea: Sandaun, MekilW100, 1718m, 14.xi.2003, 4 48.637S 141 38.994E, K. Sagata (WB 19)” (ZSM). 4 males “Papua New Guinea: Sandaun, MekilK [sic!], 1718m, 14.x.2003, 4 48.742S 141 39.075E, K. Sagata (WB 106)”, two males additionally with labels “DNA M. Balke 672” and “DNA M. Balke 681” (NHMW, ZSM). 3 males “Papua New Guinea: Sandaun, Ofektaman, 820m, 17.x.2008, 5.04.113S 141.35.841E, Ibalim (PNG 190)”, two males additionally with green labels “DNA M.Balke 3720”, “DNA M.Balke 3721” (ZSM).

#### Additional material.

2 females with the same label as the holotype (ZSM), these females might belong to two species: *Exocelina sandaunensis* sp. n. and a species of *Exocelina rivulus* group. 2 females “Papua New Guinea: Sandaun, MekilK [sic!], 1718m, 14.x.2003, 4 48.742S 141 39.075E, K. Sagata (WB 106)” (ZSM), these females might belong to two species: *Exocelina sandaunensis* sp. n. and *Exocelina ketembang* Balke, 1998. 7 females “Papua New Guinea: Sandaun, Ofektaman, 820m, 17.x.2008, 5.04.113S 141.35.841E, Ibalim (PNG 190)” (ZSM), these females might belong to three species: *Exocelina sandaunensis* sp. n., *Exocelina aipomek* Balke, 1998, and *Exocelina ketembang* Balke, 1998.

#### Diagnosis.

Beetle medium-sized, dark brown to piceous, slightly submatt; pronotum with lateral bead; male antennomeres 3–5 evidently enlarged, slightly rounded, antennomeres 3, 4 almost equal in size, antennomere 5 slightly smaller, with external margin almost straight, antennomere 6 somewhat enlarged; male protarsomere 4 with large, slender, evidently curved anterolateral hook-like seta; median lobe broad, with very weak submedian constriction in ventral view and thin apex in lateral view, apex with small lateral setae; paramere with notch on dorsal side and subdistal part elongate, with numerous, long, thick, curved at apex setae. This species is similar to *Exocelina simbaiarea* sp. n., *Exocelina tariensis* sp. n., and *Exocelina jimiensis* sp. n., see differences under their diagnoses.

#### Description.

*Size and shape*: Beetle medium-sized (TL-H 3.5–4.0 mm, TL 3.9–4.5 mm, MW 1.85–2.15 mm), with oblong-oval habitus, broadest at elytral middle. *Coloration*: Dorsal surface more or less uniform dark brown to piceous, slightly paler on clypeus, vertex, pronotal sides, and along elytral suture; head appendages and legs yellowish red, legs reddish brown distally (Fig. 41). Teneral specimens paler.

*Surface sculpture*: Head with dense punctation (spaces between punctures 1–3 times size of punctures), evidently finer and sparser anteriorly; diameter of punctures equal or smaller than diameter of cells of microreticulation. Pronotum with finer, sparser, and more evenly distributed punctation than on head. Elytra with very sparse and fine punctation. Pronotum and elytra with relatively weakly impressed microreticulation, dorsal surface slightly submatt. Head with microreticulation stronger. Metaventrite and metacoxa distinctly microreticulate, metacoxal figs with longitudinal strioles and transverse wrinkles. Abdominal ventrites with distinct microreticulation, strioles, and fine sparse punctation, coarser and denser on two last abdominal ventrites.

*Structures*: Pronotum with distinct lateral bead. Base of prosternum and neck of prosternal process and neck of prosternal process with distinct ridge, smooth and not rounded anteriorly, with small anterolateral extensions. Blade of prosternal process lanceolate, relatively narrow, convex, with distinct lateral bead and few setae; neck and blade of prosternal process evenly jointed. Abdominal ventrite 6 slightly truncate apically.

*Male*: Antennomeres 3–5 evidently enlarged, slightly rounded, antennomeres 3, 4 almost equal in size, antennomere 5 slightly smaller, with external margin almost straight, antennomere 6 somewhat enlarged; (Fig. 13A). Protarsomere 4 with large, slender, evidently curved anterolateral hook-like seta. Protarsomere 5 ventrally with anterior row of 15 and posterior row of 3 short setae (Fig. 13B). Abdominal ventrite 6 with 7–10 lateral striae on each side. Median lobe broad, with very weak submedian constriction in ventral view and thin apex in lateral view, apex with small lateral setae (Figs 13C, D). Paramere with notch on dorsal side and subdistal part elongate, with numerous, long, thick, curved at apex setae (Fig. 13E).

*Holotype*: TL-H 3.75 mm, TL 4.25 mm, MW 2 mm.

*Female*: Antennae simple, abdominal ventrite 6 without striae.

#### Distribution.

Papua New Guinea. This species is known from Sandaun Province (Fig. 53).

#### Etymology.

The species is named after Sandaun Province where it was collected. The name is an adjective in the nominative singular.

### 18. Exocelina simbaiarea

Taxon classificationAnimaliaColeopteraDytiscidae

Shaverdo & Balke sp. n.

http://zoobank.org/2C2BD156-AE8E-433D-A685-BFCABA97D27E

Figs 12 , 40

#### Type locality.

Papua New Guinea: Madang Province, Simbai area, 05°13.33'S; 144°37.61'E.

#### Type material.

*Holotype*: male “Papua New Guinea: Madang, Simbai area, 1200m, 11.iii.2007, 05.13.333S 144.37.611E, Kinibel (PNG 153)” (ZSM).

#### Additional material:

10 females with the same label as the holotype (ZSM), these females might belong to three species: *Exocelina simbaiarea* sp. n. and two species from the *Exocelina broschii*- and *Exocelina rivulus*-groups.

#### Diagnosis.

Beetle medium-sized, blackish brown, with brown head and pronotal sides, slightly submatt; pronotum with lateral bead; male antennomeres 3–5 evidently enlarged, slightly rounded, antennomeres 3, 4 almost equal in size, antennomere 5 slightly smaller, with external margin rounded, antennomere 6 somewhat enlarged; male protarsomere 4 with large, slender, evidently curved anterolateral hook-like seta; median lobe with very weak submedian constriction in ventral view and apex relatively short and slightly broadened in lateral view; paramere with notch on dorsal side and side and subdistal part elongate, with numerous, long, thick, curved at apex setae. The species is similar to *Exocelina sandaunensis* sp. n., except for slightly matter pronotum, more striated abdominal ventrite 6, male antennomere 5 with external margin rounded, and apex of median lobe shorter and broader. See also under diagnosis of *Exocelina jimiensis* sp. n.

#### Description.

*Size and shape*: Beetle medium-sized (TL-H 3.65 mm, TL 4.1 mm, MW 1.95 mm), with oblong-oval, broadest at elytral middle. *Coloration*: Head brown, darker posterior eyes and at middle; pronotum with dark brown disc and brown sides; elytra blackish brown, with reddish sutural lines; head appendages and legs reddish, legs darker distally (Fig. 40).

*Surface sculpture*: Punctation as in *Exocelina sandaunensis* sp. n.; microreticulation slightly stronger, especially on pronotum, than in *Exocelina sandaunensis* sp. n.

*Structures*: Pronotum with distinct lateral bead. Base of prosternum and neck of prosternal process and neck of prosternal process with distinct ridge, smooth and not rounded anteriorly, with small anterolateral extensions. Blade of prosternal process lanceolate, relatively narrow, convex, with distinct lateral bead and few setae; neck and blade of prosternal process evenly jointed. Abdominal ventrite 6 slightly truncate apically.

*Male*: Antennomeres 3–5 evidently enlarged, slightly rounded, antennomeres 3, 4 almost equal in size, antennomere 5 slightly smaller, with external margin rounded, antennomere 6 somewhat enlarged; (Fig. 12A). Protarsomere 4 with large, slender, evidently curved anterolateral hook-like seta. Protarsomere 5 ventrally with anterior row of 15 and posterior row of 4 short setae (Fig. 12B). Abdominal ventrite 6 with 13–14 lateral striae on each side. Median lobe with very weak submedian constriction in ventral view and apex relatively short and slightly broadened in lateral view (Fig. 12C, D). Paramere with notch on dorsal side and side and subdistal part elongate, with numerous, long, thick, curved at apex setae (Fig. 12E).

*Female*: Antennae simple, abdominal ventrite 6 without striae.

#### Distribution.

Papua New Guinea: Madang Province. This species is known from the type locality (Fig. 53).

#### Etymology.

The species is named after the Simbai area where it was collected. The name is a noun, combination of two words: “Simbai” and “area”, in the nominative singular standing in apposition.

### 19. Exocelina skalei

Taxon classificationAnimaliaColeopteraDytiscidae

Shaverdo & Balke sp. n.

http://zoobank.org/C2A429B4-D6B8-4727-BC1E-AD3D8678329C

Figs 1A–D , 28

Exocelina undescribed sp. MB4427: Toussaint et al. 2014: Supplementary figs 1–4, Tab. 2.

#### Type locality.

Indonesia: West Papua Province: Kaimana Regency, Kamaka, 03°48.37'S; 134°14.03'E.

#### Type material.

*Holotype*: male “Indonesia w-papua 50km SE Kaimana, Triton bay, vic. Kamaka vill. trail to Kamakawalar lake, S3°48’22” E134°14’02”, 50-100m, 03.II.1994 leg. A. Skale (006a) small pool” (ZSM). *Paratypes*: 4 males, 3 females with the same label as the holotype, 2 males additionally with green labels “DNA M. Balke 4426”, “DNA M. Balke 4427” (CASk, MZB, NHMW, ZSM).

#### Diagnosis.

Beetle small, broadly oval, piceous, with paler head and pronotum or only with pale anterior part of head and pronotal sides, submatt; pronotum with lateral bead; male antennomeres simple; male protarsomere 4 with medium-sized, slender, slightly curved anterolateral hook-like seta; median lobe with apical discontinuity and deeply concave, bilobed apex in ventral view; paramere without notch on dorsal side, with triangular basal part and thin subdistal part, setae inconspicuous, sparse, thin, and relatively short. This species is similar only to *Exocelina vladimiri* and probably related to it. In the group, only these two species have outline of the median lobe with apical, not submedial, discontinuity in ventral view and broadly oval habitus. *Exocelina vladimiri* can be distinguished from *Exocelina skalei* sp. n. with larger size, absence of the pronotal bead, less concave apex of the median lobe, and paramere setation.

#### Description.

*Size and shape*: Beetle small (TL-H 2.9–3.25 mm, TL 3.0–3.6 mm, MW 1.7–1.9 mm), with broadly oval habitus, broadest at elytral middle. *Coloration*: Head dark brown, sometimes to piceous between eyes and paler anteriorly; pronotum dark brown, sometimes to piceous on disc, with red to reddish brown sides; elytra uniformly dark brown to piceous; head appendages yellowish, legs darker, reddish to reddish-brown, especially metathoracic legs (Fig. 28).

*Surface sculpture*: Head with dense, coarse punctation (spaces between punctures 1–2 times size of punctures); diameter of some punctures equal diameter of cells of microreticulation. Pronotum and elytra with punctation finer and more evenly distributed than on head but very evident. Pronotum and elytra with evident microreticulation, dorsal surface submatt. Head with microreticulation stronger. Metaventrite and metacoxa distinctly microreticulate, metacoxal figs with longitudinal strioles and transverse wrinkles. Abdominal ventrites with distinct microreticulation, strioles, and fine sparse punctation, coarser and denser on two last abdominal ventrites.

*Structures*: Pronotum with distinct lateral bead. Base of prosternum and neck of prosternal process with distinct ridge, smooth and rounded anteriorly, without small anterolateral extensions. Blade of prosternal process lanceolate, broad, slightly convex, with rounded apex, distinct lateral bead and few setae; neck and blade of prosternal process evenly jointed. Abdominal ventrite 6 broadly rounded apically.

**Figure 1.**
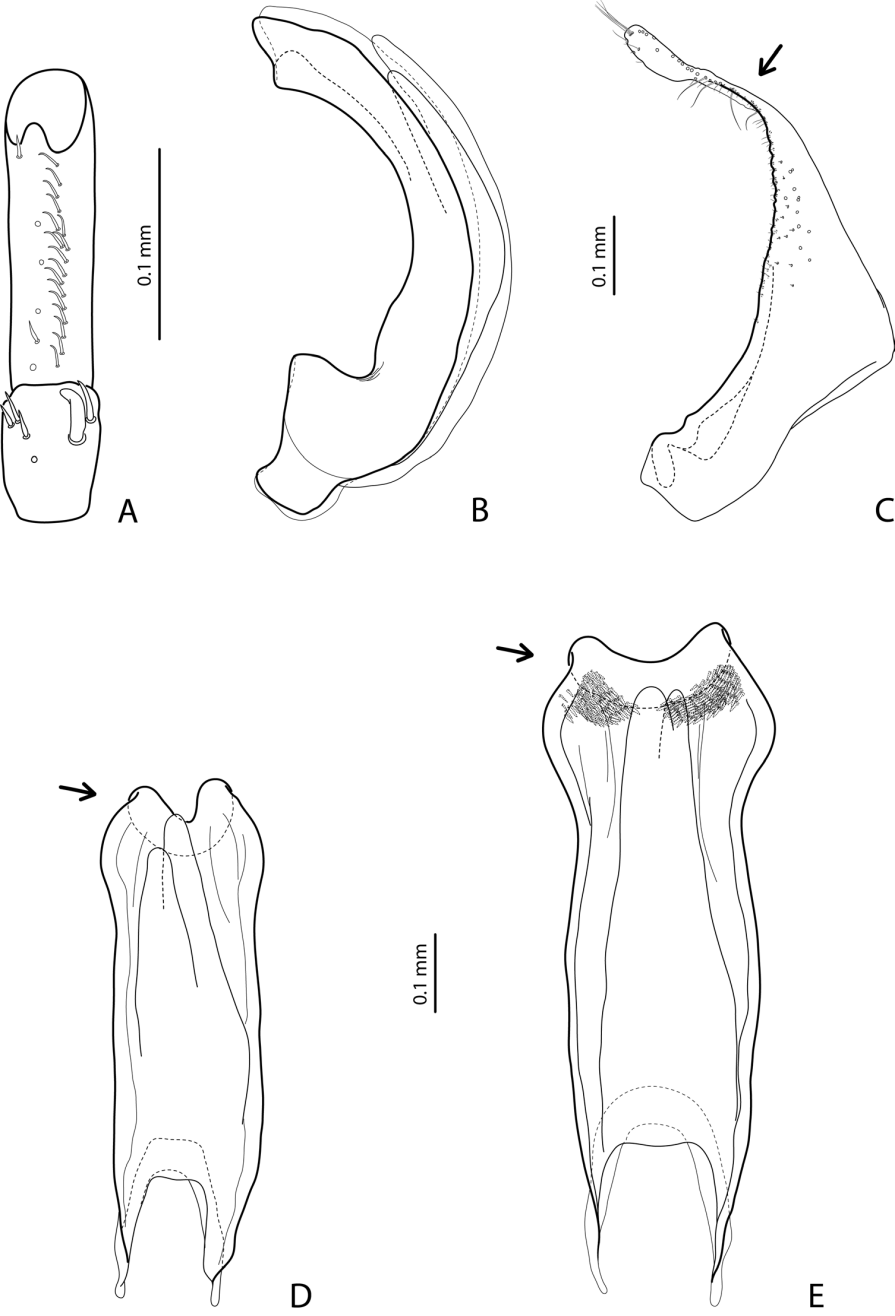
**A–D** *Exocelina skalei* sp. n. **E** *Exocelina vladimiri* (Shaverdo, Sagata & Balke, 2005) **A** male protarsomeres 4–5 in ventral view **B** median lobe in lateral view **C** paramere in external view **D**, **E** median lobe in ventral view.

*Male*: Antenna simple (Fig. 28). Protarsomere 4 with medium-sized, slender, slightly curved anterolateral hook-like seta. Protarsomere 5 ventrally with anterior row of 17 and posterior row of 6 relatively long setae (Fig. 1A). Abdominal ventrite 6 with 3–4 very short lateral striae on each side. Median lobe with apical discontinuity and deeply concave, bilobed apex in ventral view (Figs 1B, D). Paramere without notch on dorsal side, with triangular basal part and thin subdistal part, setae inconspicuous, sparse, thin, and relatively short (Fig. 1C).

*Holotype*: TL-H 3.25 mm, TL 3.6 mm, MW 1.9 mm.

*Female*: Without evident differences in external morphology from male, except for abdominal ventrite 6 without striae.

#### Distribution and habitat.

Indonesia: West Papua Province: Kaimana Regency. This species is known only from the type locality (Fig. 53). The species was collected from a small rock pool, without any vegetation (Fig. 54).

#### Etymology.

The species is named for Andre Skale who collected this species, with our sincere thanks for presenting this interesting species for study. The species name is a noun in the genitive case.

### 20. Exocelina tabubilensis

Taxon classificationAnimaliaColeopteraDytiscidae

Shaverdo & Balke sp. n.

http://zoobank.org/765B91F0-AA9F-4BE6-9497-E5D5707776DA

Figs 6 , 34

#### Type locality.

Papua New Guinea: Western Province, Tabubil, 05°15.67'S; 141°13.74'E.

#### Type material.

*Holotype*: male “Papua New Guinea: Western Province, Tabubil, 600m, 22.vi.2008, 05.15.673S 141.13.738E, Posman (PNG 181)” (ZSM). *Paratype*: 1 male “Papua New Guinea: Sandaun, Mianmin (river) 700m, 21.x.2008, 04.52.858S 141.31.706E Ibalim (PNG 197)” (ZSM).

#### Diagnosis.

Beetle medium-sized, piceous with paler head and pronotum, submatt; pronotum with distinct lateral bead; male protarsomere 4 with large, slender, evidently curved anterolateral hook-like seta. The species is similar to *Exocelina munaso* (Shaverdo, Sagata & Balke, 2005) because of shape of the median lobe (with large lateral folds in ventral view) and paramere (without notch on dorsal side). However, it differs from *Exocelina munaso* with smaller size, evidently narrower blade of prosternal process, male antennomeres 5–7 evidently enlarged, antennomeres 4, 8, 9 slightly enlarged, medial lobe much narrower, submedian constriction evident in ventral view, apex of median lobe almost rounded and not curved downwards in lateral view, and setae of paramere more numerous.

#### Description.

*Size and shape*: Beetle medium-sized (TL-H 4.15–4.2 mm, TL 4.55–4.65 mm, MW 2.3–2.35 mm), with oblong-oval habitus, broadest at elytral middle. *Coloration*: Head dark brown, reddish brown anteriorly and with two reddish brown spots on vertex; pronotum dark brown on disc and gradually paler to yellowish red lateral sides; elytra uniformly piceous; head appendages yellowish-red, legs reddish (Fig. 34).

*Surface sculpture*: Head with dense punctation (some punctures conjoint or spaces between most of them 1–3 times size of punctures), evidently finer and sparser anteriorly and posteriorly; diameter of punctures equal to diameter of cells of microreticulation. Pronotum with finer, slightly sparser, and more evenly distributed punctation than on head. Elytra with punctuation slightly coarser and denser than on pronotum. Head, pronotum and elytra with strong microreticulation and punctation, dorsal surface submatt. Metaventrite and metacoxa distinctly microreticulate, metacoxal figs with longitudinal strioles and transverse wrinkles. Abdominal ventrites with distinct microreticulation, strioles, and fine sparse punctation, coarser and denser on two last abdominal ventrites.

*Structures*: Pronotum with distinct lateral bead. Base of prosternum and neck of prosternal process and neck of prosternal process with distinct ridge, smooth and rounded anteriorly, without anterolateral extensions. Blade of prosternal process lanceolate, very narrow, convex, with distinct lateral bead and few setae, apex of blade slightly but distinctly bent upwards; neck and blade of prosternal process evenly jointed. Abdominal ventrite 6 slightly truncate apically.

*Male*: Antennomeres 5–7 evidently enlarged, antennomeres 4, 8, 9 slightly enlarged (Fig. 6A). Protarsomere 4 with large, slender, evidently curved anterolateral hook-like seta. Protarsomere 5 ventrally with anterior row of more than 40 and posterior row of 16 relatively long setae (Fig. 6B). Abdominal ventrite 6 with 15–17 lateral striae on each side. Median lobe narrow, with very strong submedian constriction and large lateral folds in ventral view and its apex almost rounded and not curved downwards in lateral view (Fig. 6C, D). Paramere without notch on dorsal side, with subdistal setae numerous (Fig. 6E).

*Holotype*: TL-H 4.15 mm, TL 4.65 mm, MW 2.35 mm.

*Female*: Unknown.

#### Distribution.

Papua New Guinea: Western and Sandaun Province. This species is known only from two localities (Fig. 53).

#### Etymology.

The species is named after the type locality: Tabubil. The name is an adjective in the nominative singular.

### 21. Exocelina tariensis

Taxon classificationAnimaliaColeopteraDytiscidae

Shaverdo & Balke sp. n.

http://zoobank.org/27224603-8DC9-43F7-BBFC-E05E1B397AFC

Figs 11 , 39

Exocelina undescribed sp. MB1289: Toussaint et al. 2014: Supplementary figs 1–4, Tab. 2.

#### Type locality.

Papua New Guinea: Hela Province, Tari, Mt. Ambua, 05°57.55'S; 143°04.99'E.

#### Type material.

*Holotype*: male “Papua New Guinea: Southern Highlands, Tari, Mt Ambua, 2100m, 14.v.2006, 05.57.550S 143.04.993E, Balke (PNG 64)”, “DNA M.Balke 1289” [green] (ZSM).

#### Diagnosis.

Beetle medium-sized, blackish brown, with brown clypeus and pronotal sides, submatt; pronotum with lateral bead; male antennomeres 3–5 evidently enlarged, with external margin more expanded, antennomeres 3, 4 almost equal in size, antennomere 5 slightly smaller, antennomere 6 somewhat enlarged; male protarsomere 4 with large, slender, slightly curved upwards anterolateral hook-like seta, with pointed apex; median lobe broad, with very weak submedian constriction in ventral view and thin apex in lateral view, apex with small lateral setae; paramere with notch on dorsal side and subdistal part small, elongate, with not numerous, long, thick, almost straight setae. The species is similar to *Exocelina sandaunensis* sp. n. and *Exocelina simbaiarea* sp. n., except for more robust habitus, slightly matter pronotum, larger male antennomeres 3–5, with external margin more expanded, pointed and slightly curved upwards anterolateral hook-like seta of male protarsomere 4, subdistal part with setae less numerous and almost straight.

#### Description.

*Size and shape*: Beetle medium-sized (TL-H 4 mm, TL 4.4 mm, MW 2.15 mm), with oblong-oval, broadest at elytral middle. *Coloration*: Dorsal surface more or less uniform blackish brown, paler on clypeus, vertex, and pronotal sides; head appendages and legs yellowish brown, legs darker distally (Fig. 39).

*Surface sculpture*: Punctation as in *Exocelina sandaunensis* sp. n. and *Exocelina simbaiarea* sp. n.; microreticulation slightly stronger, especially on pronotum, than in *Exocelina sandaunensis* sp. n. and *Exocelina simbaiarea* sp. n.

*Structures*: Pronotum with distinct lateral bead. Base of prosternum and neck of prosternal process and neck of prosternal process with distinct ridge, smooth and very slightly rounded anteriorly, with small anterolateral extensions. Blade of prosternal process lanceolate, relatively narrow, convex, with distinct lateral bead and few setae; neck and blade of prosternal process evenly jointed. Abdominal ventrite 6 slightly truncate apically.

*Male*: Antennomeres 3–5 evidently enlarged, with external margin more expanded, antennomeres 3, 4 almost equal in size, antennomere 5 slightly smaller, antennomere 6 somewhat enlarged (Fig. 11A). Protarsomere 4 with large, slender, slightly curved upwards anterolateral hook-like seta, with pointed apex. Protarsomere 5 ventrally with anterior row of 15 and posterior row of 5 short setae (Fig. 11B). Abdominal ventrite 6 with 11–13 lateral striae on each side. Median lobe broad, with very weak submedian constriction in ventral view and thin apex in lateral view, apex with small lateral setae (Fig. 11C, D). Paramere with notch on dorsal side and subdistal part small, elongate, with not numerous, long, thick, almost straight setae (Fig. 11E).

*Female*: Unknown.

#### Distribution.

Papua New Guinea: Hela Province. This species is known only from the type locality (Fig. 53).

#### Etymology.

The species is named after the village of Tari where it was collected. The name is an adjective in the nominative singular.

### 22. Exocelina vovai

Taxon classificationAnimaliaColeopteraDytiscidae

Shaverdo & Balke sp. n.

http://zoobank.org/8C7240ED-D3C7-4EAE-9FFC-79F2321D1AC8

Figs 16 , 44

Exocelina undescribed sp. MB1372: Toussaint et al. 2014: Supplementary figs 1–4, Tab. 2.

#### Type locality.

Papua New Guinea: Morobe Province, Menyamya, Mt. Inji, approximately 07°14.81S; 146°01.33E.

#### Type material.

*Holotype*: male “Papua New Guinea: Morobe, Menyamya, Mt Inji, 1900m, 14.xi.2006, nr 07.14.813S 146.01.330E, Balke & Kinibel (PNG 97)” (ZSM). *Paratypes*: 10 males, 9 females with the same label as the holotype, one male additionally with a green label “DNA M.Balke 1378” (NHMW, ZSM).

#### Additional material:

2 females “Papua New Guinea: Morobe, Menyamya, 4–5h towds Aseki, 1500-2000m, 15.xi.2006, nr 07.14.956S 146.05.687E, Balke & Kinibel (PNG 100)”, one of them additionally with a green label “DNA M.Balke 1372” (ZSM).

#### Diagnosis.

Beetle medium-sized, dark brown to piceous, with paler clypeus, vertex, and pronotal sides, matt; pronotum with distinct lateral bead; male antennomeres 3–5 evidently enlarged, almost equal in size, antennomeres 5 slightly rectangular, antennomeres 6 and 7 somewhat enlarged; male protarsomere 4 with large, slender, evidently curved anterolateral hook-like seta; median lobe with weak submedian constriction and apex evidently concave in ventral view and with apex distinctly pointed in lateral view; paramere with shallow notch on dorsal side and subdistal part elongate, with numerous, dense, more or less long, thin setae. The species is similar to *Exocelina gorokaensis* sp. n., from which differs with duller dorsal surface due to denser punctation and stronger microreticulation, as well as larger and sometimes less rounded male antennomeres 3–5, paramere with shallow notch on dorsal side, and smaller median lobe, with apex evidently concave in ventral view and distinctly pointed in lateral view. Also it is similar to *Exocelina bismarckensis* sp. n. from which differs with broader and more oval habitus, less rounded male antennomeres 3–5, narrower median lobe, with apex less rounded and evidently concave in ventral view and stronger pointed in lateral view.

#### Description.

*Size and shape*: Beetle medium-sized (TL-H 3.85–4.2 mm, TL 4.4–4.65 mm, MW 2.1–2.3 mm), with oblong-oval habitus, broadest at elytral middle. *Coloration*: Dorsal surface more or less uniform dark brown to piceous, paler on clypeus, vertex, pronotal sides, and along elytral suture; head appendages and legs yellowish red to dark reddish, legs darker distally (Fig. 44). Teneral specimens paler.

*Surface sculpture*: Head with very dense, coarse punctation (spaces between punctures 1–2 times size of punctures). Pronotum with punctation finer than on head. Elytra with punctation sparser than on pronotum. Pronotum and elytra with rather strongly impressed microreticulation, dorsal surface matt. Head with microreticulation stronger. Metaventrite and metacoxa distinctly microreticulate, metacoxal figs with longitudinal strioles and transverse wrinkles. Abdominal ventrites with distinct microreticulation, strioles, and fine sparse punctation, coarser and denser on two last abdominal ventrites.

*Structures*: Pronotum with distinct lateral bead. Base of prosternum and neck of prosternal process and neck of prosternal process with distinct ridge, smooth and not rounded anteriorly, with small anterolateral extensions. Blade of prosternal process lanceolate, relatively narrow, convex, with distinct lateral bead and few setae; neck and blade of prosternal process evenly jointed. Abdominal ventrite 6 broadly rounded or slightly truncate apically.

*Male*: Antennomeres 3–5 evidently enlarged, almost equal in size, antennomeres 5 slightly rectangular, antennomeres 6 and 7 somewhat enlarged (Fig. 16A), antennomeres 3–7 rugose ventrally. Protarsomere 4 with large, slender, evidently curved anterolateral hook. Protarsomere 5 ventrally with anterior row of 20 and posterior row of 4 short setae (Fig. 16B). Abdominal ventrite 6 with 5–10 lateral striae on each side, slightly truncate apically. Median lobe with weak submedian constriction and apex evidently concave in ventral view and with apex distinctly pointed in lateral view (Fig. 16C, D). Paramere with shallow notch on dorsal side and subdistal part elongate, with numerous, dense, more or less long, thin setae (Fig. 16E).

*Holotype*: TL-H 4.2 mm, TL 4.65 mm, MW 2.3 mm.

*Female*: Antennae simple, abdominal ventrite 6 broadly rounded apically, without striae.

#### Distribution.

Papua New Guinea: Morobe Province. This species is known only from Menyamya area (Fig. 53).

#### Etymology.

The species is named for brother of the senior author, Vladimir (Vova) Shaverdo, with her sincere thanks for his help and interest in my life.

### 23. Exocelina wannangensis

Taxon classificationAnimaliaColeopteraDytiscidae

Shaverdo & Balke sp. n.

http://zoobank.org/D68A7A92-A410-4DE6-BCD7-60BCD6528E22

Figs 7 , 35

Exocelina undescribed sp. MB3761: Toussaint et al. 2014: Supplementary figs 1–4, Tab. 2.

#### Type locality.

Papua New Guinea: Madang Province, Usino, 05°31.13'S; 145°25.32'E.

#### Type material.

*Holotype*: male “Papua New Guinea: Madang, Usino, 260m, 15.iii.2007, 05.31.125S 145.25.316E, Kinibel (PNG 158)” (ZSM). *Paratypes*: 11 males, 14 females with the same label as the holotype (NHMW, ZSM). 3 males, 2 females “Papua New Guinea: Madang, Wannang, 270m 31.x.2008, 05.15.458S 145.02.389E, Posman, (PNG187)” (NHMW, ZSM). 6 males, 7 females “Papua New Guinea: Madang, Wannang, 230m 3.x.2008, 05.17.235S 145.06.160E, Posman (PNG188)”, two males additionally with green labels “DNA M.Balke 3761”, “DNA M.Balke 3762” (NHMW, ZSM).

#### Diagnosis.

Beetle small, with head and pronotum red to reddish brown and elytra dark brown, shiny; pronotum with narrow, in some specimens indistinct lateral bead; male antennomeres modified: antennomeres 3–5 larger and more rounded than other, antennomeres 6, 7 somehow enlarged; male protarsomere 4 with large, thick, strongly curved anterolateral hook-like seta; median lobe slender, with strong submedian constriction in ventral view and elongate apex in lateral view; paramere with strong notch on dorsal side and subdistal part slightly elongate, broad, with long, dense, relatively thick setae. The species is similar to the complex of the following species: *Exocelina edeltraudae*, *Exocelina pseudoedeltraudae* sp. n., *Exocelina jimiensis* sp. n., *Exocelina tariensis* sp. n., *Exocelina simbaiarea* sp. n., and *Exocelina sandaunensis* sp. n. But it differs from all of them with its smaller size, narrow pronotal lateral bead, less modified male antennomeres, and structure and setation of the male genitalia.

#### Description.

*Size and shape*: Beetle small (TL-H 2.95–3.50 mm, TL 3.25–3.90 mm, MW 1.55–1.85 mm), with oblong-oval habitus, broadest at elytral middle. *Coloration*: Head red to reddish brown, darker posterior eyes; pronotum red to reddish brown, darker on disc; elytra uniformly dark brown; head appendages red to reddish brown, legs darker, especially metathoracic legs (Fig. 35).

*Surface sculpture*: Head with dense punctation (spaces between punctures 1–3 times size of punctures), evidently finer and sparser anteriorly; diameter of punctures smaller than diameter of cells of microreticulation. Pronotum with much sparser and finer punctation than on head. Elytra with extremely sparse and fine punctation, almost invisible. Pronotum and elytra with weakly impressed microreticulation, dorsal surface shiny. Head with microreticulation stronger. Metaventrite and metacoxa distinctly microreticulate, metacoxal figs with longitudinal strioles and transverse wrinkles. Abdominal ventrites with distinct microreticulation, strioles, and fine sparse punctation, coarser and denser on two last abdominal ventrites.

*Structures*: Pronotum with narrow lateral bead. Some specimens with pronotal lateral bead indistinct and/or reduced at posterior angles. Base of prosternum and neck of prosternal process with distinct ridge, smooth and slightly rounded anteriorly, without anterolateral extensions. Blade of prosternal process lanceolate, relatively broad, convex, with distinct lateral bead and few setae; neck and blade of prosternal process evenly jointed. Abdominal ventrite 6 broadly rounded apically.

*Male*: Antenna modified: antennomeres 3–5 larger and more rounded than other, antennomeres 6, 7 somehow enlarged (Fig. 7A). Protarsomere 4 with large, thick, strongly curved anterolateral hook-like seta. Protarsomere 5 ventrally with anterior row of 10 short setae and posterior row of 6 short setae (Fig. 7B). Abdominal ventrite 6 with 6–8 lateral striae on each side. Median lobe slender, with strong submedian constriction in ventral view and elongate apex in lateral view (Fig. 7C, D). Paramere with strong notch on dorsal side and subdistal part slightly elongate, broad, with long, dense, relatively thick setae (Fig. 7E).

*Holotype*: TL-H 3.35 mm, TL 3.65 mm, MW 1.8 mm.

*Female*: Without evident differences in external morphology from males, except for simple antennae and abdominal ventrite 6 without striae.

#### Distribution.

Papua New Guinea. This species is known only from Madang Province (Fig. 53).

#### Etymology.

The name refers to the village of Wannang where this species was first discovered. The name is an adjective in the nominative singular.

### Faunistic and morphological notes

#### Exocelina arfakensis

Taxon classificationAnimaliaColeopteraDytiscidae

Shaverdo, Hendrich & Balke, 2012

##### Records.

**Indonesia: West Papua** (additional record): 11 males, 6 females “Indonesia: Papua Barat, Arfak Mts., near Minyambouw, stream in forest, 1668m, 9.xi.2013, -1,10489175 133,88603192, UNIPA (BH037)”, two males additionally with labels “M.Balke 6199”, “M.Balke 6200” (MZB, NHMW, ZSM).

##### Distribution.

Indonesia: West Papua Province: Manokwari Regency. So far, the species is known only from the Arfak Mountains, in the eastern part of the Bird’s Head.

#### Exocelina bifida

Taxon classificationAnimaliaColeopteraDytiscidae

Shaverdo, Hendrich & Balke, 2012

Fig. 20

##### Records.

**Papua New Guinea: Sandaun Province** (first record): 6 males, 5 females “Papua New Guinea: Sandaun, Mianmin area, >600m, 13.i.2010, Ibalim & Pius (PNG236)”, two males additionally with labels “DNA M. Balke 4926” and “DNA M. Balke 4927” (NHMW, ZSM). 8 males, 6 females “Papua New Guinea: Sandaun, Mianmin area, >600m, 13.i.2010, Ibalim & Pius (PNG235)” (NHMW, ZSM). 4 males, 3 females “Papua New Guinea: Sandaun, Mianmin area, >700m, 14.i.2010, 04 54.540S 141 36.953E, Ibalim & Pius (PNG238)” (NHMW, ZSM). 3 males, 3 females “Papua New Guinea: Sandaun, Mianmin (river), 700m, 21.x.2008, 04.52.858S 141.31.706E, Ibalim (PNG 197)” (ZSM). 1 female “Papua New Guinea: Sandaun, Mianmin, 1080m, 24.x.2008, 04.55.780S 141.38.185E, S. Ibalim PNG195” (ZSM). 1 male, 1 female “Papua New Guinea: Sandaun, Ofektaman, 820m, 17.x.2008, 5.04.113S 141.35.841E, Ibalim (PNG 190)”, male additionally with a label “DNA M. Balke 3722” (ZSM).

##### Morphological notes.

Beetles are smaller (TL-H 3.1–3.55 mm, TL 3.45–3.85 mm, MW 1.7–1.9 mm) than ones from the type locality: IN: Papua, Jayawijaya, Borme, Tarmlu. Also they have a slightly different shape of the median lobe of the aedeagus: the apex is less concave and narrower in ventral view and the right lateral side broader than in *Exocelina bifida* (Fig. 20C). Differences in the setation of the protarsi and parameres are most likely within in the limits of intraspecific variability (Figs 20B, E). In order to establish the status of these specimens, additional material from the border area, IN: Papua, Jayawijaya / PNG: Sandaun, is needed.

##### Distribution.

Central part of the New Guinea Island, i.e., Indonesia: Papua Province, Jayawijaya Regency and Papua New Guinea: Sandaun Province.

#### Exocelina brahminensis

Taxon classificationAnimaliaColeopteraDytiscidae

Shaverdo, Hendrich & Balke, 2012

##### Records.

**Papua New Guinea: Eastern Highlands Province** (first record): 17 males “Papua New Guinea: Eastern Highlands, Bena Bridge, 1400m, 8.xii.2007, 06.10.781S 145.26.034E, Balke & Sagata (PNG 164)”.

##### Distribution.

Papua New Guinea. This is one of the most widely distributed species in Papua New Guinea. It is known from the Momase Region: Sandaun, East Sepik, Madang, and Morobe Provinces (Shaverdo et al. 2012) and, now, also from Highlands Region: Eastern Highlands Province.

#### Exocelina knoepfchen

Taxon classificationAnimaliaColeopteraDytiscidae

Shaverdo, Hendrich & Balke, 2012

##### Records.

**Papua New Guinea: Simbu** (first record): 1 male, 2 females “Papua New Guinea Simbu prov L. Cizek lgt.”, “Kundiawa, Mu vill. 145°02'E, 4°42'S [6°05'S; 145°02'E] III.2001, 1900m” (ZSM).

##### Distribution.

Papua New Guinea: Eastern Highlands and Simbu Provinces. The present record is an extension of the known distribution of the species to the northwest.

#### Exocelina polita

Taxon classificationAnimaliaColeopteraDytiscidae

(Sharp, 1882)

##### Records.

**Indonesia: West Papua** (additional record): 13 males, 13 females “Indonesia: Papua Barat, Manokwari to Kebar, forest stream, 302m, 3.xi.2013, -0,80058566 133,33216397, UNIPA (BH023)”, one male additionally with a label “M.Balke 6186” (MZB, NHMW, ZSM). 36 males 78 females “Indonesia: Papua Barat, Kebar to Aibogar, slow forest stream, 503m, 4.xi.2013, -0,86241595 132,82993928, UNIPA (BH025)”, one male additionally with a label “M.Balke 6191”, the females are a mixture of *Exocelina polita* and one undescribed species (NHMW, ZSM). 6 males, 9 females “Indonesia: Papua Barat, Kebar to Manokwari, 1 h from Kebar, limesone creek and roadside pools, 331m, 8.xi.2013, -0,80138488 133,32238254, UNIPA (BH035)”, one male and one female additionally with labels “M.Balke 6197” and “M.Balke 6198” respectively (MZB, NHMW, ZSM).

##### Morphological notes.

Most specimens are darker than the holotype, piceous, with dark brown head and pronotal sides. It is obvious that the holotype, with its dark brown dorsal coloration, is a slightly teneral specimen. Some variability (narrower apex in lateral view) in the shape of the apical part of the median lobe is observed.

##### Distribution.

Indonesia: West Papua Province: Manokwari Regency. So far, this species has been known only as its holotype from Arfak Mountains. The present records are an extension of the known distribution of the species to the northwest.

#### Exocelina pseudosoppi

Taxon classificationAnimaliaColeopteraDytiscidae

Shaverdo, Hendrich & Balke, 2012

##### Records.

**Indonesia: Papua** (additional record), **Jayapura Regency** (first record): 2 males “Indonesia, Papua, Sentani-Lereh road, 415m, 27.ix.2014, -2.6524433 140.0164157, Menufandu (Pap034)” (MZB).

##### Distribution.

Indonesia: Papua Province: Nabire, Paniai, and Jayapura Regencies. So far, this species has been known only from the Nabire-Enarotali region. The present record is an extension of the known distribution of the species to the northeast.

### Key to all described species of the *Exocelina ekari*-group

This key is a modified version of the key to species of the *Exocelina ekari*-group from Shaverdo et al. (2012). It is based mostly on male characters. In many cases females cannot be assigned to species due to the similarity of their external and internal structures (for female genitalia see figs 17a and 17b in Shaverdo et al. (2005) and fig. 7C in Shaverdo et al. (2013)). Some species are rather similar in external morphology, therefore, in most cases the male genitalia need to be studied for reliable species identifications. Numbers in brackets refer to the order of the new species descriptions above.

**Table:** 

1	Outline of median lobe with weak apical discontinuity in ventral view (Fig. 1D, E; fig. 6 in Shaverdo et al. (2005)), beetles broadly oval, with evident punctation and microreticulation dorsally, paramere without notch on dorsal side, with triangular basal part and thin subdistal part (Fig. 1C; fig. 16b in Shaverdo et al. (2005))	**2**
–	Outline of median lobe more strongly discontinuous in ventral view, usually in submedial part (e.g., Figs 3D, 3E, 4C), beetles oblong-oval, with different punctation and microreticulation dorsally, paramere with or without notch on dorsal side, with basal and subdistal parts of different shape	**3**
2	Pronotum without lateral bead, beetle larger, TL-H: 3.6–3.7 mm, reddish-brown to dark brown, apex of median lobe slightly concave in ventral view (Fig. 1E; fig. 6 in Shaverdo et al. (2005)), paramere with distinct setae (fig. 16b in Shaverdo et al. (2005))	***vladimiri* (Shaverdo, Sagata & Balke, 2005)**
–	Pronotum with distinct lateral bead, beetle smaller, TL-H: 2.9–3.2 mm, dark brown to piceous (Fig. 32), apex of median lobe deeply concave in ventral view (Fig. 1D), paramere with inconspicuous setae (Fig. 1C)	(19) ***skalei* sp. n.**
3	Pronotum with distinct lateral bead, broad or narrow	**4**
–	Pronotum without lateral bead or with weak traces of lateral bead	**33**
4	Male antennomeres simple or slightly modified: antennomeres 3–7 very slightly enlarged (almost indistinctly), antennomere 3 slightly more triangular than other antennomeres or antennomeres 3–9 stout, with 4–5 slightly larger than other	**5**
–	Male antennomeres 3–5 or 5–7 evidently enlarged	**14**
5	Beetle larger, TL-H: 3.9–5.0 mm, piceous	**6**
–	Beetle smaller, TL-H: 3.05–4.1 mm, reddish-brown to piceous	**7**
6	Beetle larger, TL-H: 4.8–5.0 mm (fig. 24 in Shaverdo et al. (2012)), male protarsomere 4 with large, thick, strongly curved anterolateral hook-like seta, apex of median lobe pointed and curved downwards in lateral view (figs 10, 15a in Shaverdo et al. (2005))	***munaso* (Shaverdo, Sagata & Balke, 2005)**
–	Beetle smaller, TL-H: 3.9–4.1 mm (fig. 25 in Shaverdo et al. (2012)), male protarsomere 4 with medium-sized, slender, evidently curved anterolateral hook-like seta, apex of median lobe almost rounded in lateral view (figs 9, 14a in Shaverdo et al. (2005))	***atowaso* (Shaverdo, Sagata & Balke, 2005)**
7	Male antenna simple, with antennomeres thin	**8**
–	Male antennomeres slightly modified, stout, with antennomere 3 slightly more triangular or 4–5 slightly larger than other antennomeres (Fig. 5A; figs 2A, 3A in Shaverdo et al. 2012)	**12**
8	Beetle larger, TL-H: 3.8–3.9 mm, MW: 2.0–2.15 mm, dorsally with distinct punctation, submatt	**9**
–	Beetle smaller, TL-H: 3.05–3.8 mm, MW: 1.6–2.05 mm, dorsally with very fine punctation, almost invisible, shiny	**11**
9	Median lobe short and with extremely strongly discontinuous (curved, plicate) outline, paramere with shallow notch on dorsal side and subdistal part elongate, with dense, long, thin setae (Figs 3B–E; figs 37, 46, 64 in Balke (1998))	**10**
–	Median lobe longer and without such a strong modification, paramere with strong notch on dorsal side (Fig. 2C–E)	(11) ***michaelensis* sp. n.**
10	Median lobe without notch on left side in ventral view (Fig. 3E; fig. 64 in Balke (1998)), protarsomere 4 with medium-sized, slender, slightly curved anterolateral hook-like seta	***astrophallus*** (Balke, 1998)
–	Median lobe without notch on left side in ventral view (Fig. 3D), protarsomere 4 with large, thick, strongly curved anterolateral hook-like seta	(13) ***pseudoastrophallus* sp. n.**
11	Beetle reddish brown to brown dorsally, median lobe more slender in ventral view, paramere with strong notch on dorsal side and subdistal part short and large (figs 26, 1C–F in Shaverdo et al. 2012)	***oceai* Shaverdo, Hendrich & Balke, 2012**
–	Beetle piceous dorsally (Fig. 36), median lobe broader in ventral view, paramere with weaker notch on dorsal side and subdistal part elongate (Fig. 4C–F)	(3) ***craterensis* sp. n.**
12	Male antennomeres antennomeres 3–9 stout, with 4–5 slightly larger than other, median lobe with very strong median constriction and proximal part very broad in ventral view, apex of median lobe broad, slightly concave in middle and twisted at both sides in ventral view and shortly pointed in lateral view, subdistal part of paramere elongate, with a large brush of long, dense, thin setae, proximal setae almost invisible (Fig. 5)	(5) ***herowana* sp. n.**
–	Male antennomeres 3–7 very slightly enlarged, antennomere 3 slightly more triangular than other antennomeres, median lobe with median constriction weaker and proximal part narrower in ventral view, apex of median lobe of different shape, subdistal part of paramere short and small, with less numerous, short, thick, and flattened setae, proximal setae distinct (figs 2, 3 in Shaverdo et al. 2012)	**13**
13	Beetle smaller, TL-H: 3.45–3.7 mm, MW: 1.8–2.0 mm, apex of median lobe elongate in lateral view (figs 28, 2D in Shaverdo et al. 2012)	***waigeoensis* Shaverdo, Hendrich & Balke, 2012**
–	Beetle larger, TL-H: 3.75–4.1 mm, MW: 1.9–2.2 mm, apex of median lobe truncate in lateral view (figs 29, 3D in Shaverdo et al. 2012)	***evelyncheesmanae* Shaverdo, Hendrich & Balke, 2012**
14	Male antennomeres 5–7 evidently enlarged, antennomeres 4, 8, 9 slightly enlarged, male protarsomere 4 with large, slender, evidently curved anterolateral hook-like seta (Figs 6A, B), median lobe and paramere as in Figs 6C–F	(20) ***tabubilensis* sp. n.**
–	Male antennomeres 3–5 evidently enlarged	**15**
15	Male antennomeres 3–5 enlarged, more or less rounded, almost equal in size and shape	**16**
–	Male antennomeres 3 or 3–4 distinctly more modified in shape (triangular) and larger than other antennomeres	**27**
16	Punctation of dorsal surface, especially on elytra, very fine and sparse, sometimes almost invisible, beetle dorsally shiny	**17**
–	Punctation of dorsal surface very distinct, coarser and denser, beetle submatt or matt	**23**
17	Beetle smaller, TL-H: 2.95–3.50 mm, MW: 1.55–1.85 mm, pronotal lateral bead narrow (Fig. 39), protarsomere 4 with large, thick, strongly curved anterolateral hook-like seta (Fig. 7B), paramere with strong notch on dorsal side and subdistal part slightly elongate and broad (Fig. 7E), median lobe as in Fig. 7C–F	(23) ***wannangensis* sp. n.**
–	Beetle larger, TL-H: 3.3–4.45 mm, MW: 1.9–2.55 mm, pronotal lateral bead distinct, broader, paramere with shallow or distinct notch on dorsal side and subdistal part elongate and narrower	**18**
18	Median lobe with very strong median constriction and proximal part very broad in ventral view, apex of median lobe pointed and strongly curved downwards in lateral view (Figs 8E, 9D), male abdominal ventrite 6 slightly to distinctly concave apically (Fig. 8C)	**19**
–	Median lobe evenly broad, with distinctly weaker median constriction in ventral view, apex of median lobe not strongly curved downwards, male abdominal ventrite 6 slightly truncate or broadly rounded apically	**20**
19	Male antennomeres 3–5 smaller and less rounded, for antennomeres 3 and 4, ratio width/length: < 0.92 (Fig. 8A), dorsal surface slightly shinier due to weaker punctation and microreticulation (Fig. 41), apex of median lobe broader in lateral view (Fig. 8D)	***edeltraudae* Shaverdo, Hendrich & Balke, 2012**
–	Male antennomeres 3–5 more strongly enlarged and rounded, for antennomeres 3 and 4, ratio width/length: > 1.0 (Fig. 9A; fig. 4A in Shaverdo et al. 2012), dorsal surface less shinier due to stronger punctation and microreticulation (Fig. 42; fig. 30 Shaverdo et al. 2012), apex of median lobe narrower in lateral view (Fig. 9E; fig. 4C Shaverdo et al. 2012)	(15) ***pseudoedeltraudae* sp. n.**
20	Male antennomeres 3–5 more strongly enlarged, antennomere 5 smaller than antennomeres 4–5, median lobe with very weak submedian constriction in ventral view and thin apex in lateral view, subdistal part of paramere with setae longer and thicker, less numerous	**21**
–	Male antennomeres 3–5 smaller, almost equal in size, median lobe with stronger submedian constriction in ventral view and more broadened apex in lateral view, subdistal part of paramere with setae shorter and thinner, more numerous (Fig. 10)	(6) ***jimiensis* sp. n.**
21	Beetle dorsally matter, especially pronotum, male antennomeres 3–5 more strongly enlarged, with external margin more expanded, male protarsomere 4 with large, slender, slightly curved upwards anterolateral hook-like seta, with pointed apex, subdistal part of paramere with setae less numerous (Fig. 11)	(21) ***tariensis* sp. n.**
–	Beetle dorsally shinier, especially pronotum, male antennomeres 3–5 smaller, male protarsomere 4 with large, slender, curved but not upwards anterolateral hook-like seta, with more or less rounded apex, subdistal part of paramere with setae more numerous	**22**
22	Male antennomere 5 with external margin rounded (Fig. 12A), abdominal ventrite 6 with 13–14 striae, apex of median lobe shorter (Fig. 12D)	(18) ***simbaiarea* sp. n.**
–	Male antennomere 5 with external margin almost straight (Fig. 13A), abdominal ventrite 6 with 7–10 striae, apex of median lobe longer (Fig. 13D)	(17) ***sandaunensis* sp. n.**
23	Beetle smaller, TL-H: 3.3–3.75 mm, MW: 1.9–2.1 mm	**24**
–	Beetle larger, TL-H: 3.6–4.45 mm, MW: 1.95–2.4 mm	**25**
24	Beetle dorsally brightly ferrugineous to castaneous, submatt, with punctation coarse and dense (fig. 31 in Shaverdo et al. 2012), apex of median lobe broader in ventral view, paramere with shallow notch on dorsal side (figs 5C, E in Shaverdo et al. 2012)	***hansferyi* Shaverdo, Hendrich & Balke, 2012**
–	Beetle dorsally dark brown, almost shiny, with punctation less coarse and dense (fig. 32 in Shaverdo et al. 2012), apex of median lobe narrower in ventral view, paramere with distinct notch on dorsal side (figs 6C, E in Shaverdo et al. 2012)	***bundiensis* Shaverdo, Hendrich & Balke, 2012**
25	Paramere with distinct notch on dorsal side (Fig. 14E). Dorsal punctation finer (Fig. 46)	(4) ***gorokaensis* sp. n.**
–	Paramere with shallow notch on dorsal side (Figs 15E, 16E). Dorsal punctation coarser (Figs 47, 48)	**26**
26	Habitus more elongate, often with subparallel sides (Fig. 47), apex of median lobe almost rounded in ventral view, with curved part gradually pointed in lateral view (Fig. 15D)	(2) ***bismarckensis* sp. n.**
–	Habitus more oval (Fig. 48), apex of median lobe not rounded, distinctly concave in ventral view, with curved part more sharply pointed in lateral view (Fig. 16D)	(22) ***vovai* sp. n.**
27	Male antennomere 3 much larger than other antennomeres, triangular, beetle larger, TL-H: 3.8–4.8 mm, MW: 2.0–2.55 mm, male protarsomere 4 with anterolateral hook very small (smaller than more laterally situated large seta), thin, and slightly curved, paramere distinctly longer than median lobe, without notch on dorsal side, with relatively short, sparse, thin setae	**28**
–	Male antennomeres 3 and 4 much larger than other antennomeres, triangular, beetle smaller, TL-H: 3.7–4.3 mm, MW: 2.05–2.3 mm, male protarsomere 4 with anterolateral hook thin or thick, slightly curved but larger than more laterally situated large seta, paramere equal or shorter than median lobe, with notch on dorsal side, setae of subdistal part not numerous, relatively short, thick, and flattened	**30**
28	Beetle larger, TL-H: 4.5–4.8 mm, MW: 2.35–2.55 mm, dorsally shiny, with fine, indistinct punctuation, male antennomeres 3 and 4 smaller (figs 33, 7A in Shaverdo et al. (2012))	***knoepfchen* Shaverdo, Hendrich & Balke, 2012**
–	Beetle smaller, TL-H: 3.8–4.5 mm, MW: 2.0–2.5 mm, dorsally submatt or matt, with coarse, distinct punctuation, male antennomeres 3 and 4 larger	**29**
29	Beetle larger, TL-H: 4.3–4.5 mm, MW: 2.35–2.5 mm, dorsally matt, with microreticulation stronger (Fig. 49), antennomere 3 smaller, more triangular, median lobe with apex more rounded in lateral view, male protarsomere 4 with anterolateral hook-like seta smaller than more laterally situated large seta, paramere with less numerous subdistal long setae and, especially, internal spines (Fig. 17), abdominal ventrite 6 with 7–9 lateral striae on each side	(7) ***kisli* sp. n.**
–	Beetle smaller, TL-H: 3.8–4.2 mm, MW: 2.0–2.3 mm, dorsally submatt, with microreticulation weaker (Fig. 50), male antennomere 3 larger, with external margin more rounded, median lobe with apex pointed in lateral view, male protarsomere 4 with anterolateral hook-like seta larger than more laterally situated large seta, paramere with more numerous subdistal long setae and internal spines (Fig. 18), abdominal ventrite 6 with 14–17 lateral striae on each side	(8) ***ksionseki* sp. n.**
30	Male antennomeres 3 and 4 more strongly elongated, more equal in size and shape, elytral punctation fine, coloration dark brown to piceous, apex of median lobe almost truncate in lateral view, paramere narrower (figs 8A, 34, 8D, E in Shaverdo et al. (2012))	***alexanderi* Shaverdo, Hendrich & Balke, 2012**
–	Male antennomeres 3 and 4 less elongated, antennomere 3 larger than 4, coloration and elytral punctation different, median lobe with apex elongate in lateral view, paramere broader	**31**
31	Beetle dorsally ferrugineous, submatt, with coarse punctation, male protarsomere 4 with anterolateral hook thin (figs 35, 9B in Shaverdo et al. (2012)), median lobe and paramere as in figs 9C–E in Shaverdo et al. (2012)	***anggiensis* Shaverdo, Hendrich & Balke, 2012**
–	Beetle dorsally brown to piceous, shiny, with distinctly finer punctation, male protarsomere 4 with anterolateral hook thin or thick, median lobe and paramere different	**32**
32	Beetle dorsally piceous, with elytral punctation fine but distinct, male protarsomere 4 with thick anterolateral hook (figs 36, 10B in Shaverdo et al. (2012)), median lobe and paramere as in figs 10C–E in Shaverdo et al. (2012)	***arfakensis* Shaverdo, Hendrich & Balke, 2012**
–	Beetle dorsally brown, with elytral punctation almost invisible, male protarsomere 4 with thin anterolateral hook (figs 37, 11B in Shaverdo et al. (2012)), median lobe and paramere as in figs 11C–E in Shaverdo et al. (2012)	***polita* (Sharp, 1882)**
33	Male antennomeres 3 and 4 strongly enlarged, 5 less enlarged, and 2, 6–9 slightly enlarged	**34**
–	Male antennomeres simple or antennomeres 3–10 slightly enlarged (stout)	**35**
34	Beetle reddish-brown to brown, apex of median lobe symmetrical in ventral view (figs 38, 12C in Shaverdo et al. (2012))	***irianensis* Shaverdo, Hendrich & Balke, 2012**
–	Beetle dark brown to piceous, apex of median lobe asymmetrical in ventral view (figs 39, 13C in Shaverdo et al. (2012))	***wondiwoiensis* Shaverdo, Hendrich & Balke, 2012**
35	Sternite 7 slightly or strongly concave apically, median lobe long, with very weak submedian constriction and narrow apex in ventral view, paramere large, with strong notch on dorsal side and subdistal part very broad, subquadrate (fig. 14C–F in Shaverdo et al. (2012))	***utowaensis* Shaverdo, Hendrich & Balke, 2012**
–	Sternite 7 broadly rounded or truncate apically, median lobe distinctly shorter, paramere smaller, with weaker notch on dorsal side and subdistal part small and short or elongate	**36**
36	Apex of median lobe bifid: with small dorsal extension in lateral view	**37**
–	Apex of median lobe not bifid in lateral view	**38**
37	Apex of median lobe with small dorsal extension weaker in lateral view (Fig. 19D)	(14) ***pseudobifida* sp. n.**
–	Apex of median lobe with small dorsal extension stronger in lateral view (Fig. 20D; fig. 15D in Shaverdo et al. (2012))	***bifida* Shaverdo, Hendrich & Balke, 2012**
38	Apex of median lobe very strongly protruding, forming long, thin prolongation in lateral view, ventral sclerite apically divided in three parts (Fig. 24C, D)	(12) ***pinocchio* sp. n.**
–	Apex of median lobe broadly or narrowly elongate or almost truncate but never with long, thin prolongation in lateral view, ventral sclerite apically divided in two parts	**39**
39	Beetle larger, TL-H: 3.4–3.7 mm (fig. 42 in Shaverdo et al. (2012)), paramere with subdistal part small and short, with not numerous, relatively short, thick, and flattened setae, apical part of median lobe very broad in ventral view and slightly flattened in lateral view, (fig. 16C–E in Shaverdo et al. (2012))	***ekari* Shaverdo, Hendrich & Balke, 2012**
–	Beetle smaller, TL-H: 3.0–3.6 mm, paramere with subdistal part short or elongate, setation different, apical part of median lobe different, usually narrower in ventral view	**40**
40	Paramere with subdistal part short and more rounded (e.g., Fig. 25E)	**41**
–	Paramere with subdistal part elongate (e.g., Fig. 29E)	**43**
41	Median lobe slender, especially its apical part, subdistal part of paramere with not numerous, relatively short, thick, flattened, slightly curved at apex setae (fig. 17C–E in Shaverdo et al. (2012))	***weylandensis* Shaverdo, Hendrich & Balke, 2012**
–	Median lobe more robust, subdistal part of paramere with more numerous setae	**42**
42	Male protarsomere 4 with medium-sized, slender anterolateral hook-like seta (fig. 18B in Shaverdo et al. (2012)), prosternal ridge evidently rounded and smooth, median lobe with apex broader in lateral view, subdistal part of paramere with thinner setae (fig. 18C–E in Shaverdo et al. (2012))	***soppi* Shaverdo, Hendrich & Balke, 2012**
–	Male protarsomere 4 with large, thick, strongly curved anterolateral hook-like seta (Figs 21B, 22B, 23B), prosternal ridge anteriorly evidently less rounded and smooth, median lobe with apex narrower in lateral view, subdistal part of paramere with thicker, somewhat flattened setae (Figs 21C–E, 22C–E, 23C–E)	(1) ***bewaniensis* sp. n.**
43	Subdistal part of paramere with numerous long, dense, thin or thick but never flattened setae (e.g., fig. 22E in Shaverdo et al. (2012))	**44**
–	Subdistal part of paramere with at least some flattened setae (e.g., Fig. 29E)	**45**
44	Male antennomeres simple, median lobe with apex almost truncate in lateral view and submedian constriction stronger in ventral view, paramere with setae of proximal part longer, thicker, distinctly visible (fig. 22A, C–E in Shaverdo et al. (2012))	***kakapupu* Shaverdo, Hendrich & Balke, 2012**
–	Male antennomeres 3–10 stout, median lobe with apex elongate in lateral view and submedian constriction weaker in ventral view, paramere with setae of proximal part shorter, thiner, often hardly visible (figs 23A, C–E in Shaverdo et al. (2012))	***unipo*** Shaverdo, Hendrich & Balke, 2012
45	Subdistal part of paramere with a strong tuft of thicker, somewhat flattened, and strongly curved at apex setae, median lobe with apex truncate in lateral view (figs 19 C–E in Shaverdo et al. (2012))	***pseudosoppi* Shaverdo, Hendrich & Balke, 2012**
–	Subdistal part of paramere with more numerous setae, not forming a tuft, median lobe with apex distinctly more elongate in lateral view	**46**
46	Subdistal part of paramere elongate but broad, only with thick, flattened setae, except for a very few short fine distal setae	**47**
–	Subdistal part of paramere evidently narrower, with two kinds of setae: thin upper setae and thick and flattened lower setae	**48**
47	Apex of median lobe broadly elongate in lateral view and almost broadly rounded in ventral view, paramere with shallow notch on dorsal side, subdistal part of paramere with distal flattened setae longer, proximal part of paramere with setae short, almost invisible (Fig. 25C–E)	(10) ***mantembu* sp. n.**
–	Apex of median lobe almost truncate in lateral view and deeply concave in ventral view, paramere with strong notch on dorsal side, subdistal part of paramere with proximal flattened setae longer, proximal part of paramere with setae long, evident (Fig. 26C–E)	(9) ***lembena* sp. n.**
48	Median lobe longer, its apex almost truncate in lateral view, paramere on dorsal side with notch tip sharply pointed, subdistal part of paramere with upper thin setae more numerous and lower flattened setae shorter and thicker (figs 21A, C–E in Shaverdo et al. (2012))	***brahminensis* Shaverdo, Hendrich & Balke, 2012**
–	Median lobe shorter, its apex slightly elongate in lateral view, paramere on dorsal side with notch tip broadly rounded, subdistal part of paramere with upper thin setae less numerous and lower flattened setae longer, thinner, and curved at apex	**49**
49	Apex of median lobe broader and asymmetrical in ventral view, subdistal part of paramere with upper thin setae more, male protarsomere 4 with thick anterolateral hook-like seta (fig. 20B–E in Shaverdo et al. (2012))	***eme* Shaverdo, Hendrich & Balke, 2012**
–	Apex of median lobe narrower and symmetrical in ventral view, subdistal part of paramere with upper thin setae less numerous, male protarsomere 4 with slender anterolateral hook-like seta (Fig. 27B–E)	(16) ***pseudoeme* sp. n.**

**Figure 2.**
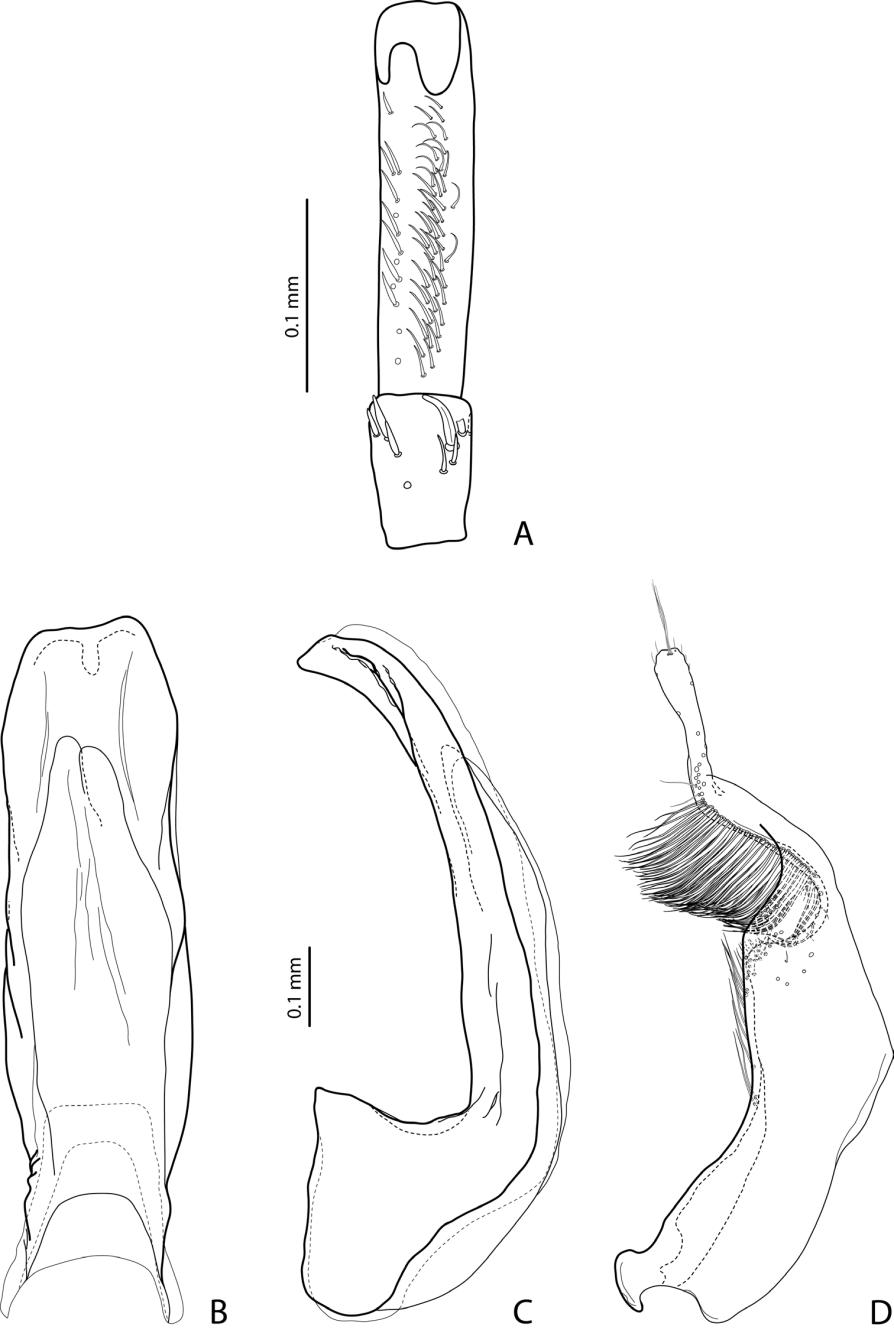
*Exocelina michaelensis* sp. n. **A** male protarsomeres 4–5 in ventral view **B** median lobe in ventral view **C** median lobe in lateral view **D** paramere in external view.

**Figure 3.**
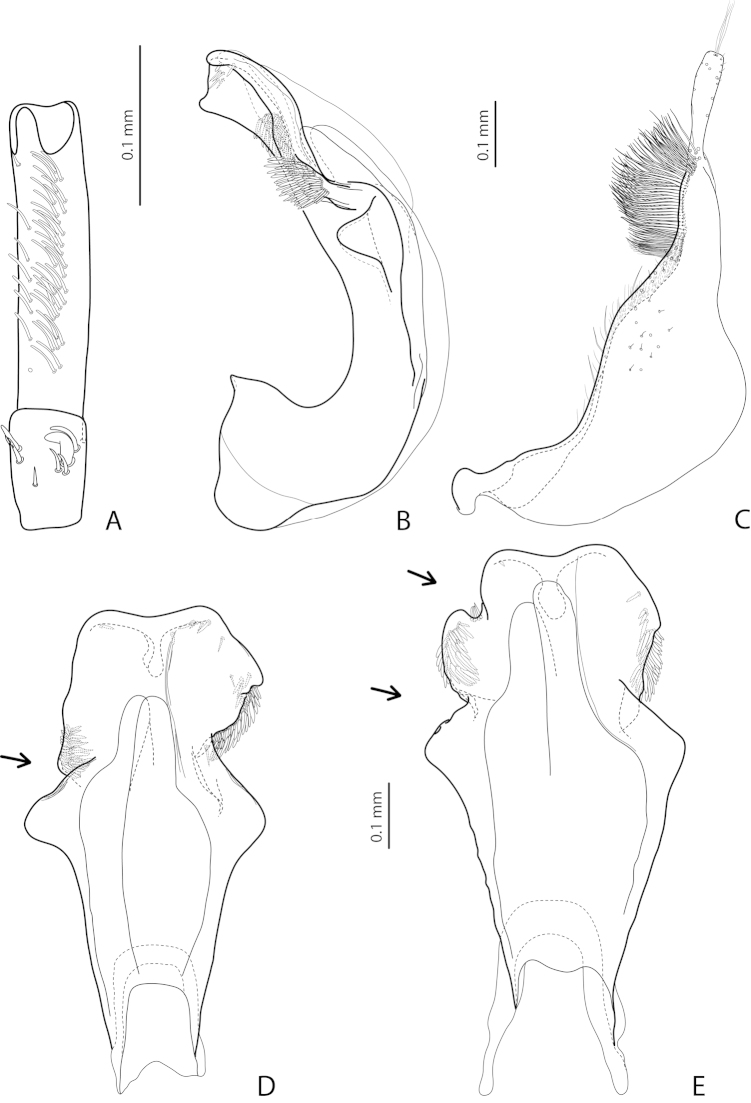
**A–D** *Exocelina pseudoastrophallus* sp. n. **E** *Exocelina astrophallus* (Balke, 1998), near Madang **A** male protarsomeres 4–5 in ventral view **B** median lobe in lateral view **C** paramere in external view **D**, **E** median lobe in ventral view.

**Figures 4, 5.**
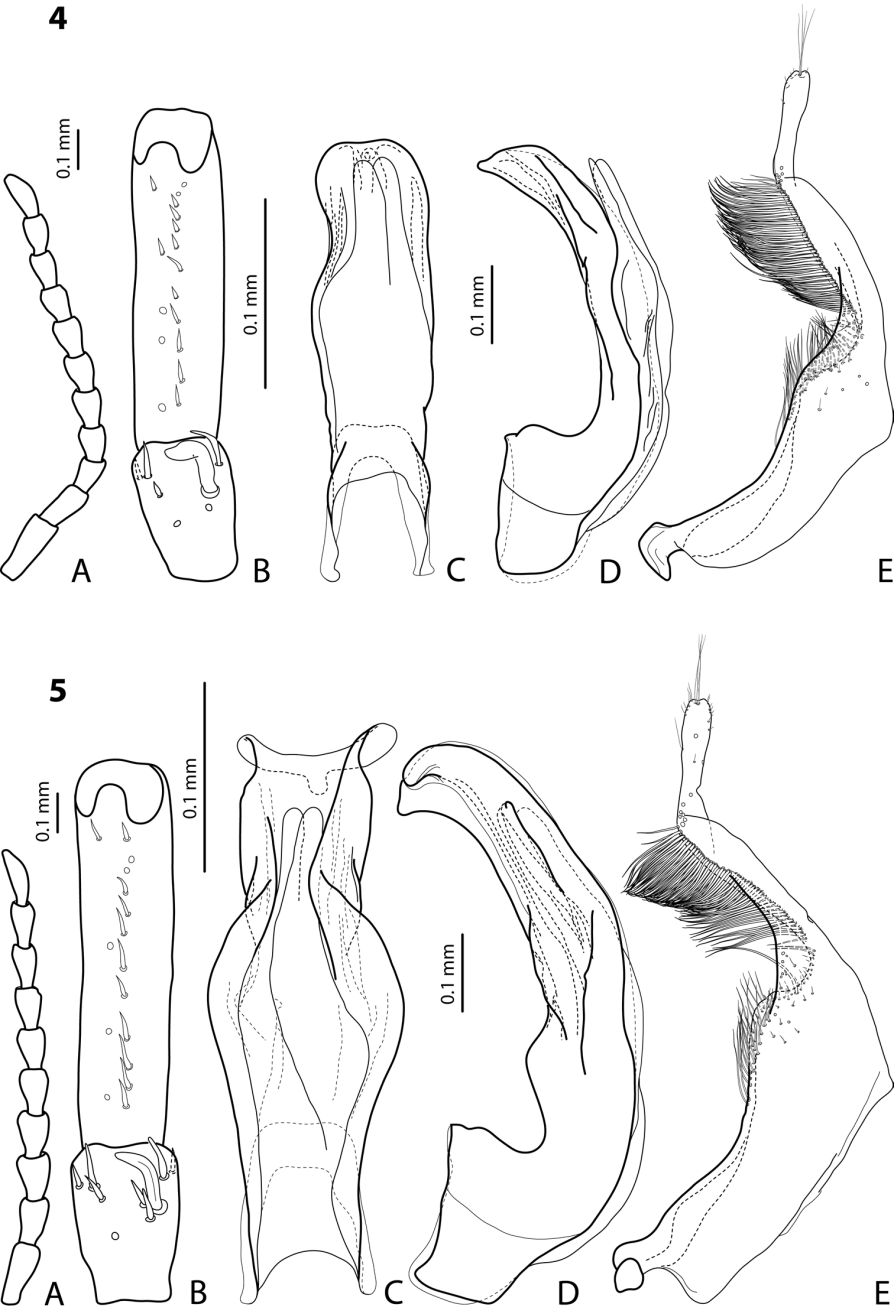
**4** *Exocelina craterensis* sp. n. **5** *Exocelina herowana* sp. n. **A** male antenna **B** male protarsomeres 4–5 in ventral view **C** median lobe in ventral view **D** median lobe in lateral view **E** paramere in external view.

**Figure 6.**
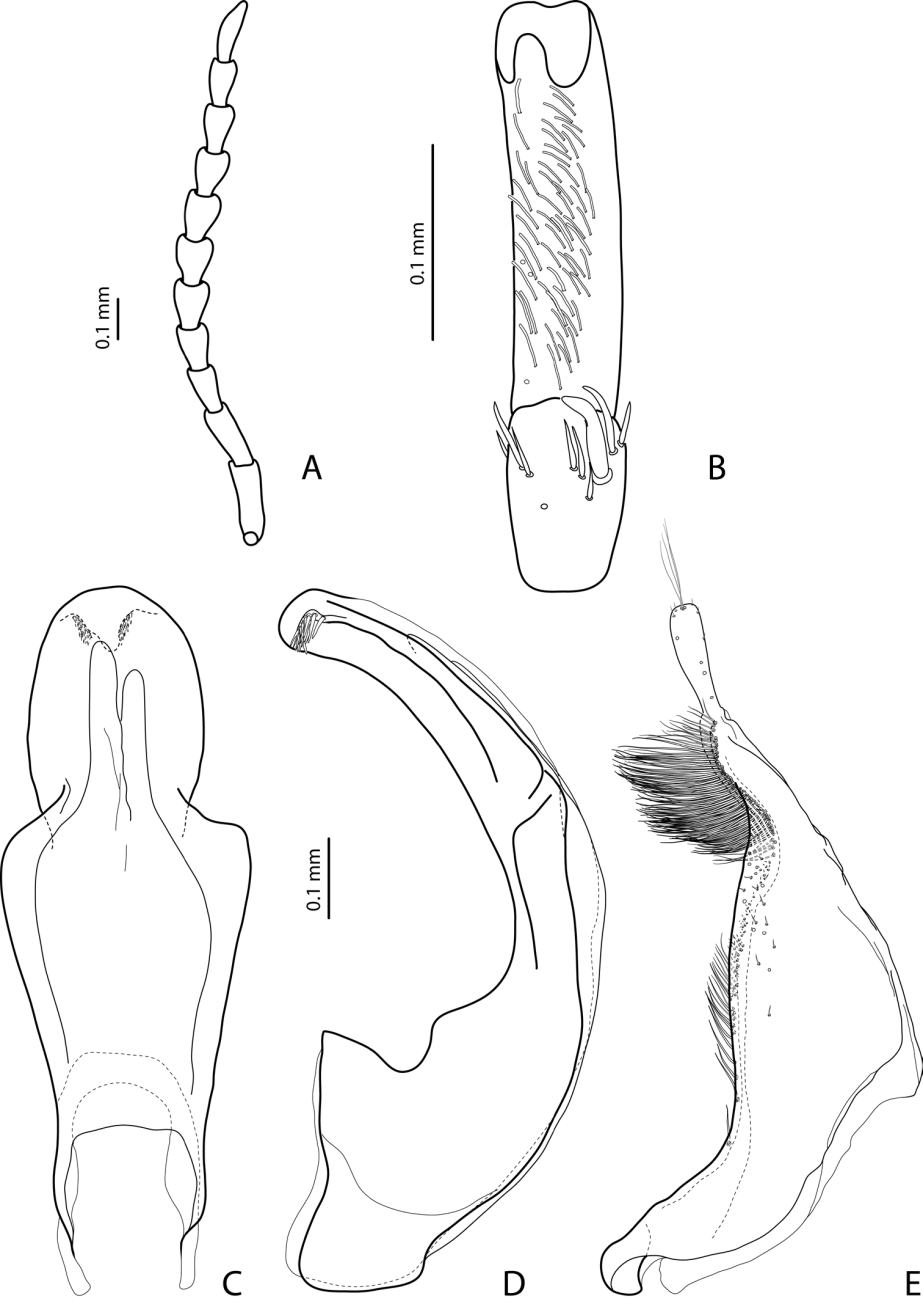
*Exocelina tabubilensis* sp. n. **A** male antenna **B** male protarsomeres 4–5 in ventral view **C** median lobe in ventral view **D** median lobe in lateral view **E** paramere in external view.

**Figure 7.**
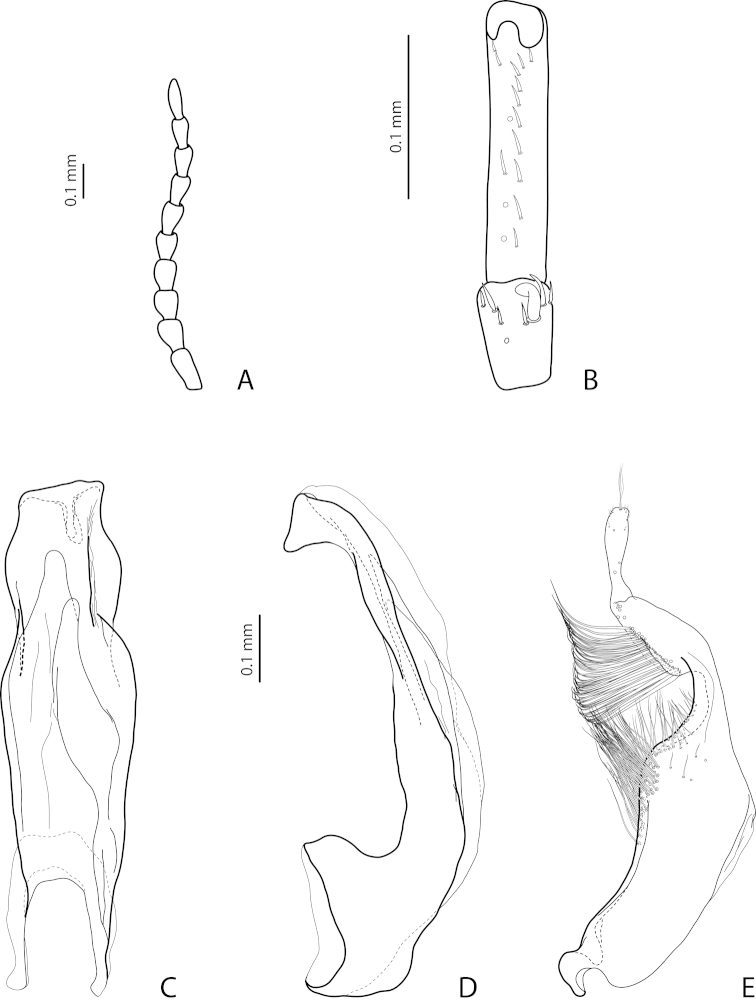
*Exocelina wannangensis* sp. n. **A** male antenna **B** male protarsomeres 4–5 in ventral view **C** median lobe in ventral view **D** median lobe in lateral view **E** paramere in external view.

**Figure 8.**
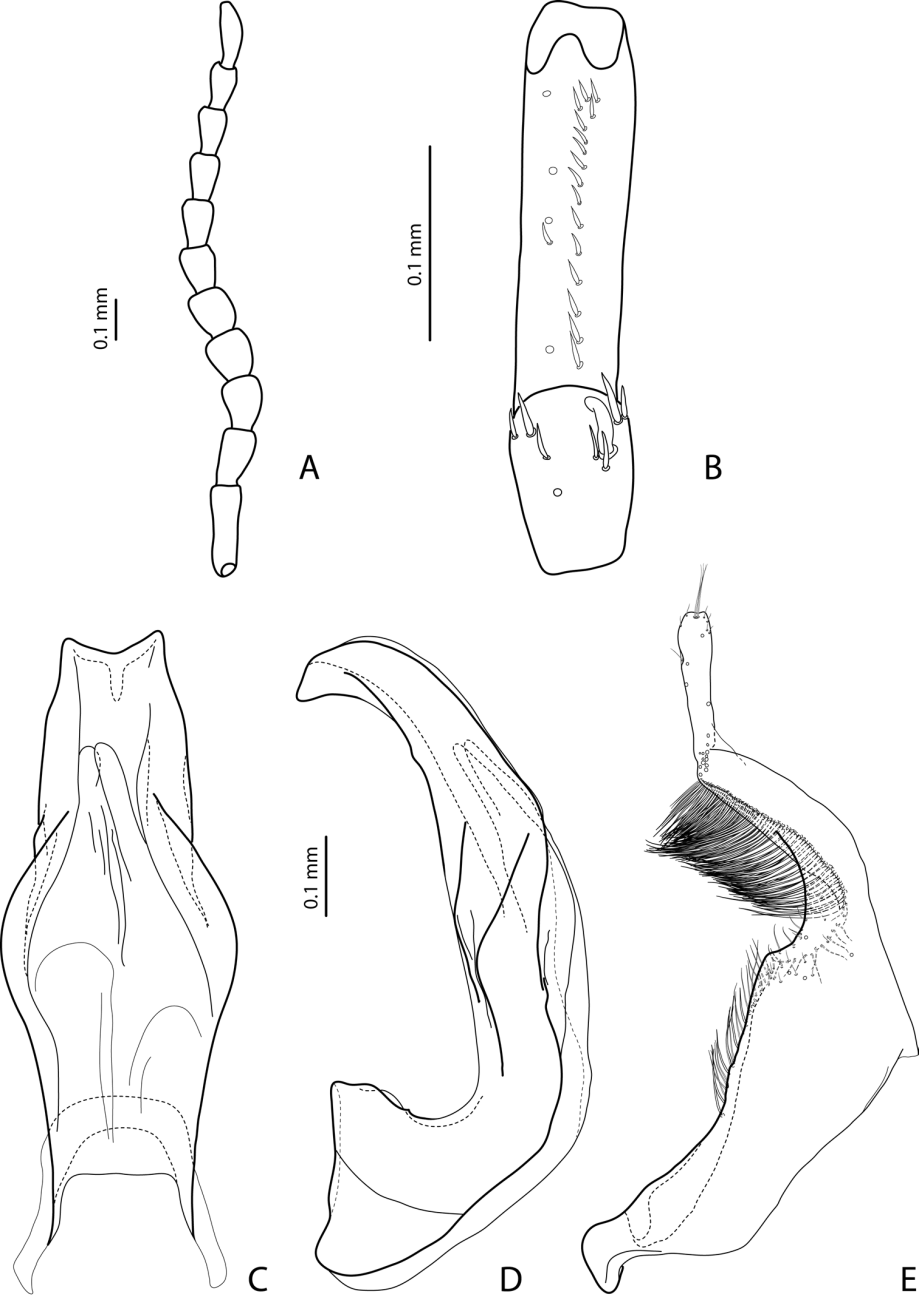
*Exocelina edeltraudae* Shaverdo, Hendrich & Balke, 2012 **A** male antenna **B** male protarsomeres 4–5 in ventral view **C** median lobe in ventral view **D** median lobe in lateral view **E** paramere in external view.

**Figure 9.**
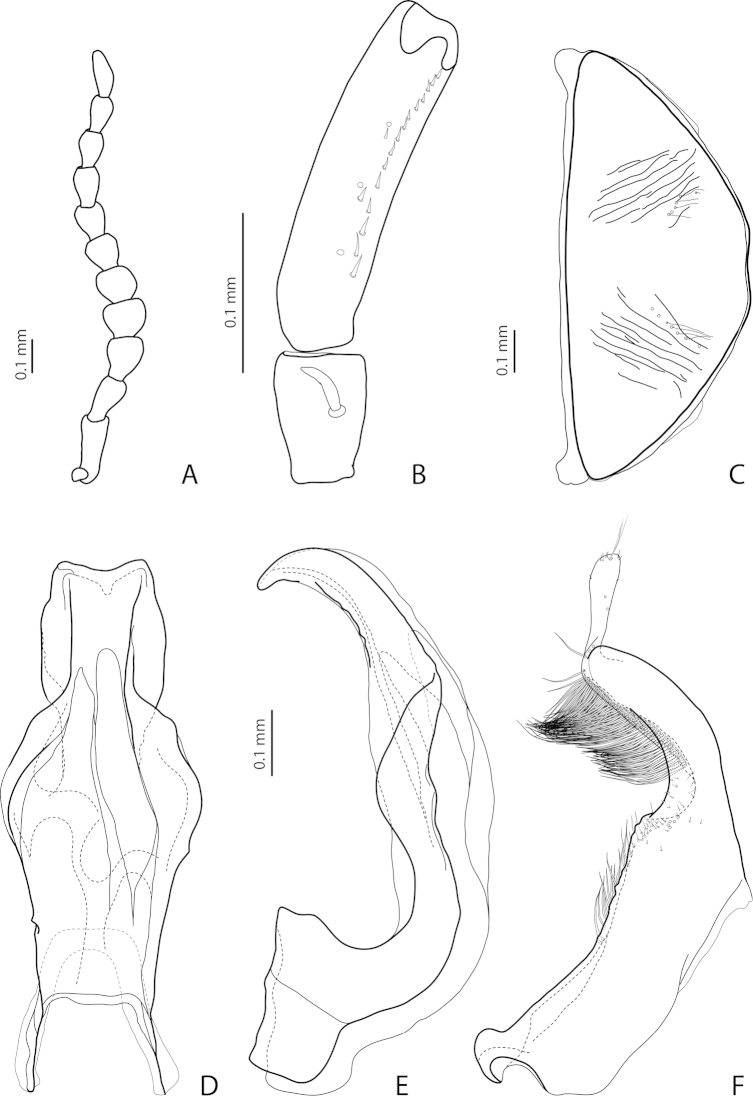
*Exocelina pseudoedeltraudae* sp. n. from Shaverdo et al. (2012, fig. 4) **A** male antenna **B** male protarsomeres 4–5 in ventral view **C** abdominal ventrite 6 **D** median lobe in ventral view **E** median lobe in lateral view **F** paramere in external view.

**Figure 10.**
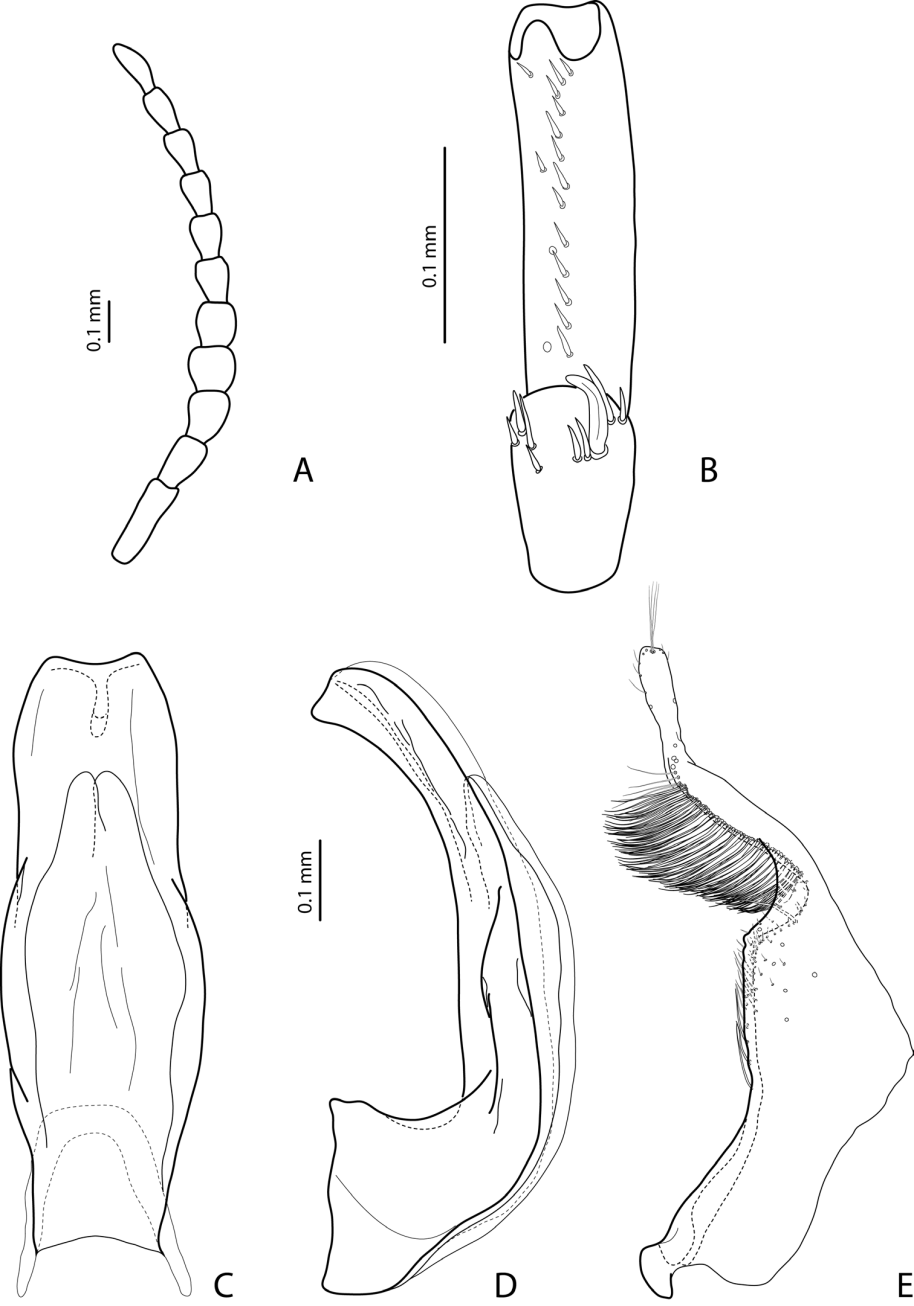
*Exocelina jimiensis* sp. n. **A** male antenna **B** male protarsomeres 4–5 in ventral view **C** median lobe in ventral view **D** median lobe in lateral view **E** paramere in external view.

**Figure 11.**
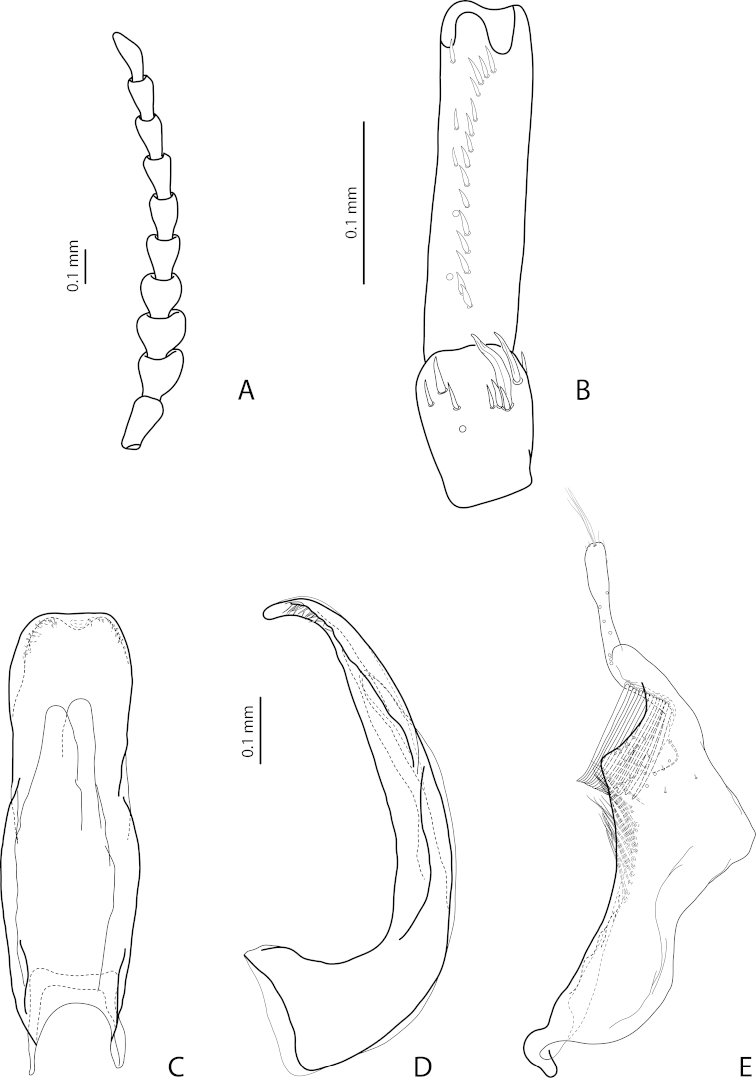
*Exocelina tariensis* sp. n. **A** male antenna **B** male protarsomeres 4–5 in ventral view **C** median lobe in ventral view **D** median lobe in lateral view **E** paramere in external view.

**Figure 12.**
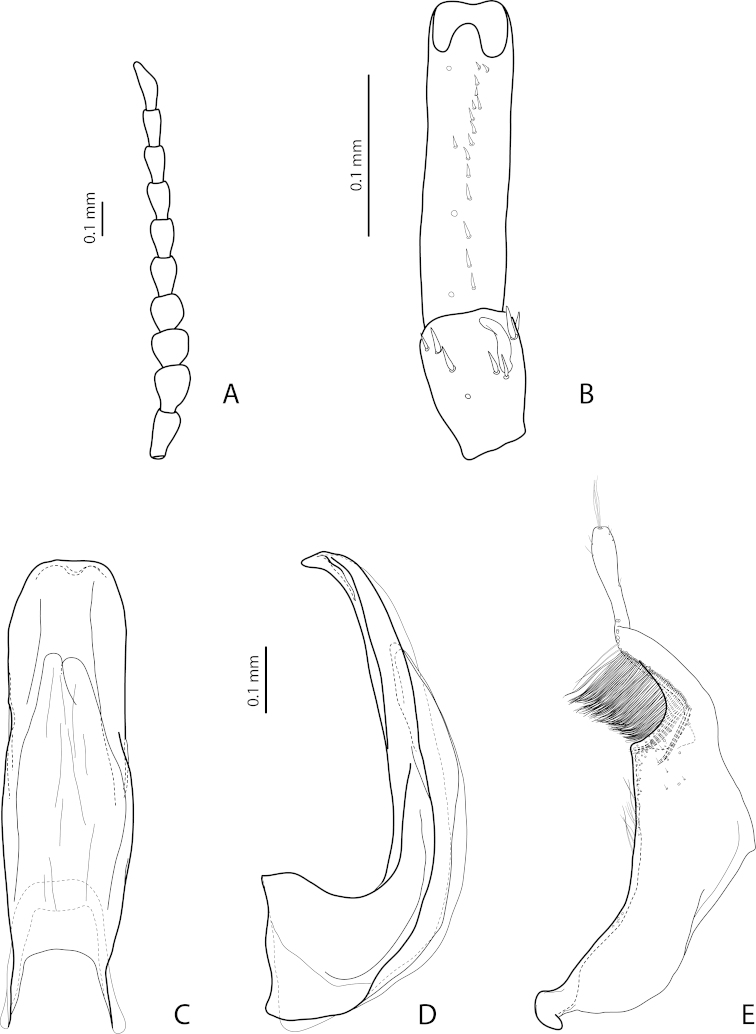
*Exocelina simbaiarea* sp. n. **A** male antenna **B** male protarsomeres 4–5 in ventral view **C** median lobe in ventral view **D** median lobe in lateral view **E** paramere in external view.

**Figure 13.**
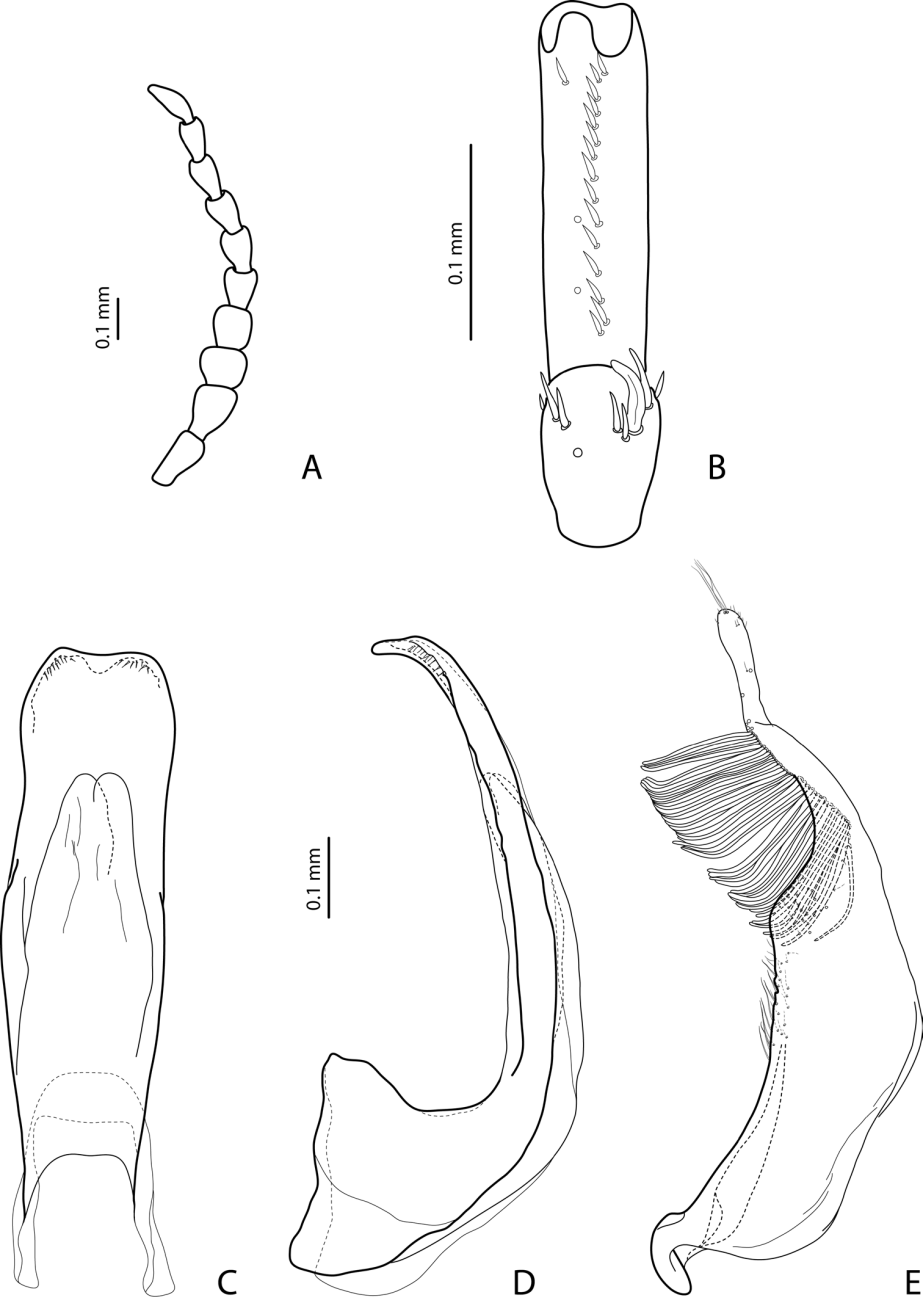
*Exocelina sandaunensis* sp. n. **A** male antenna **B** male protarsomeres 4–5 in ventral view **C** median lobe in ventral view **D** median lobe in lateral view **E** paramere in external view.

**Figure 14.**
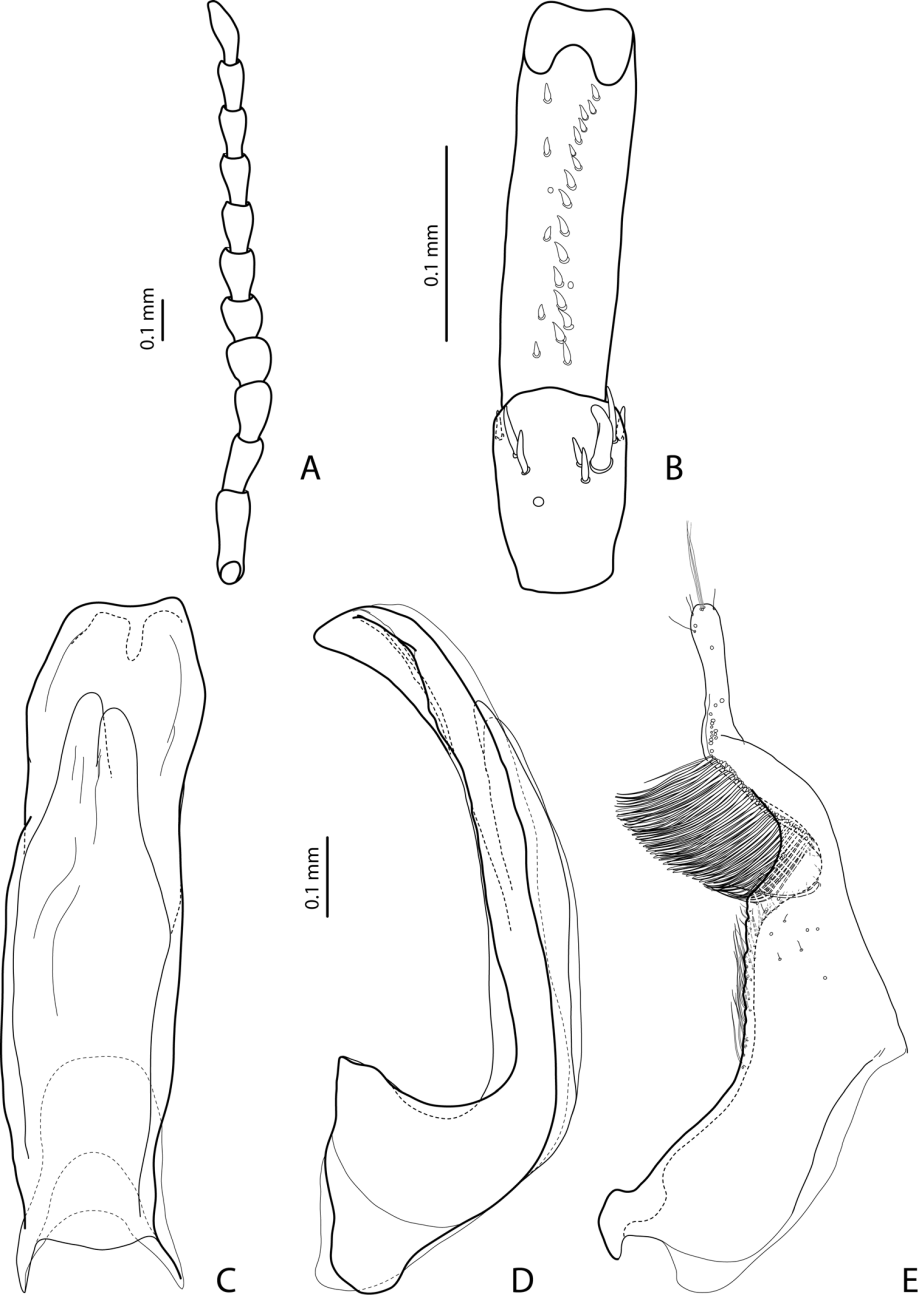
*Exocelina gorokaensis* sp. n. **A** male antenna **B** male protarsomeres 4–5 in ventral view **C** median lobe in ventral view **D** median lobe in lateral view **E** paramere in external view.

**Figure 15.**
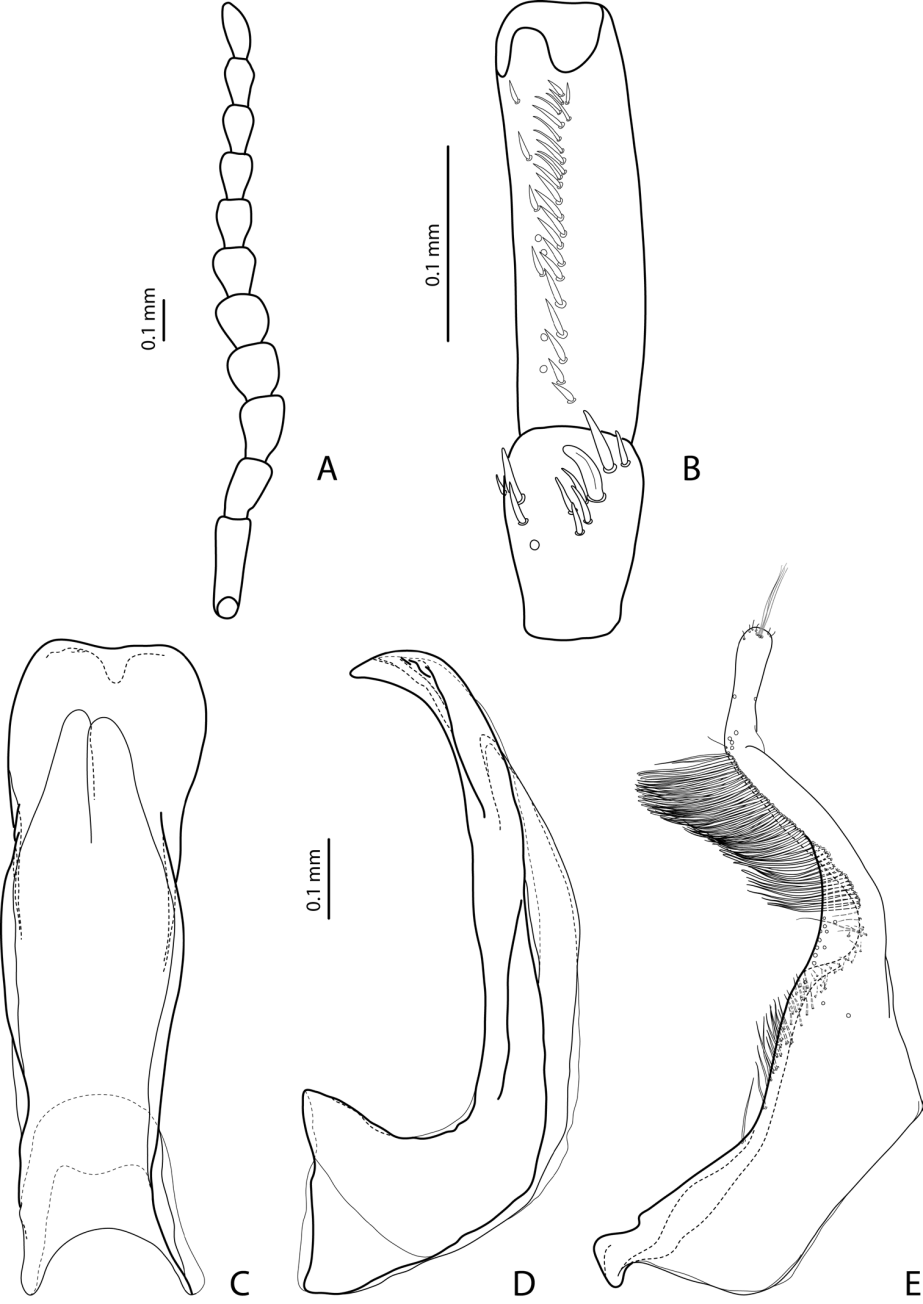
*Exocelina bismarckensis* sp. n. **A** male antenna **B** male protarsomeres 4–5 in ventral view **C** median lobe in ventral view **D** median lobe in lateral view **E** paramere in external view.

**Figure 16.**
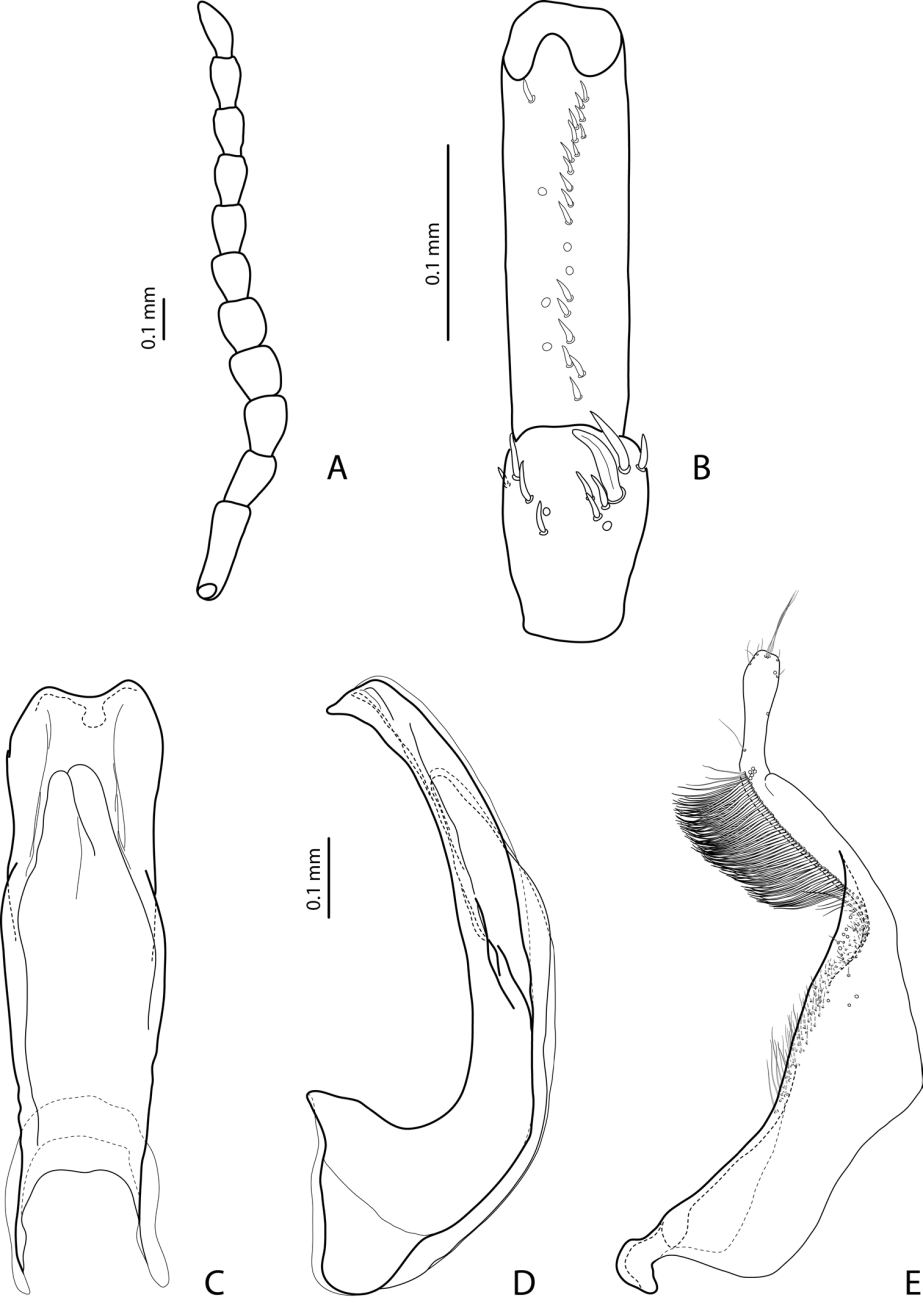
*Exocelina vovai* sp. n. **A** male antenna **B** male protarsomeres 4–5 in ventral view **C** median lobe in ventral view **D** median lobe in lateral view **E** paramere in external view.

**Figure 17.**
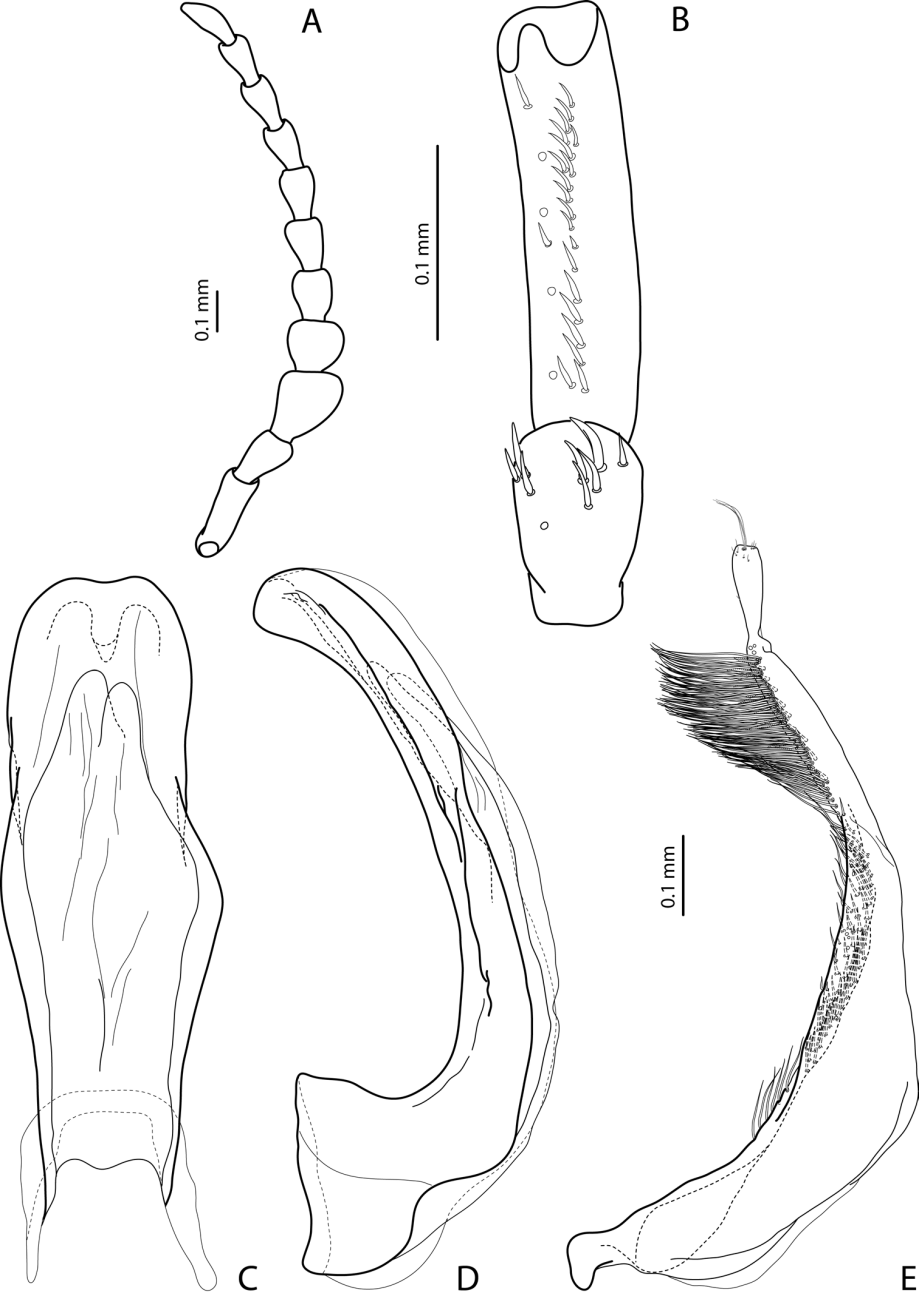
*Exocelina kisli* sp. n. **A** male antenna **B** male protarsomeres 4–5 in ventral view **C** median lobe in ventral view **D** median lobe in lateral view **E** paramere in external view.

**Figure 18.**
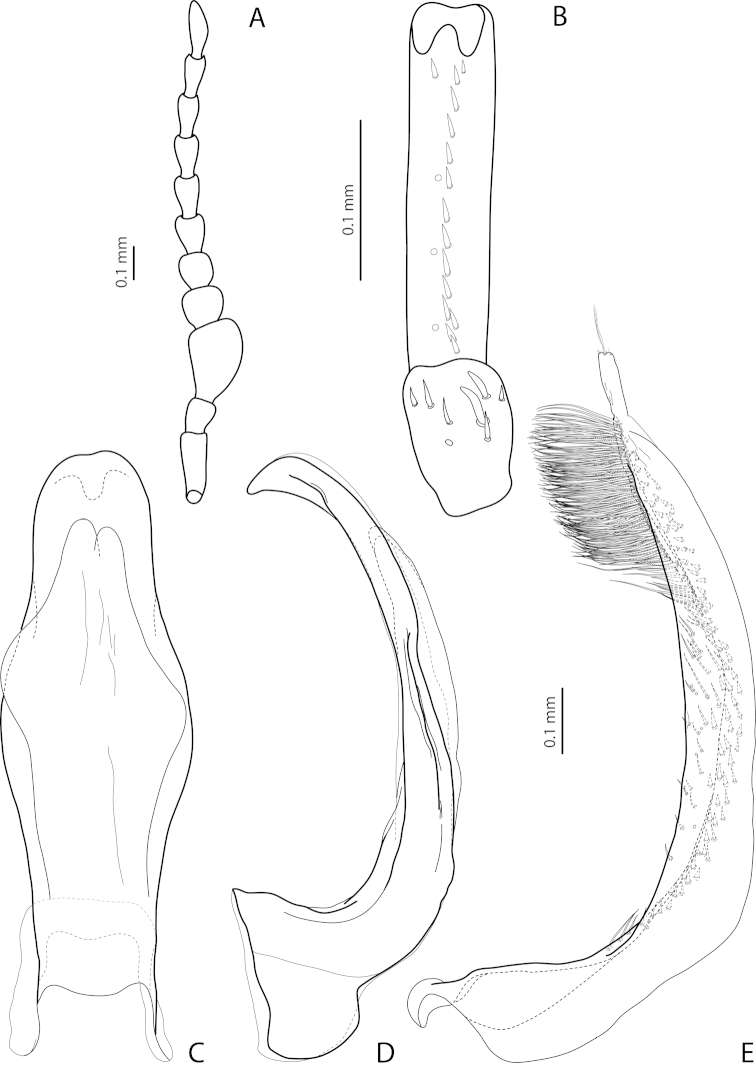
*Exocelina ksionseki* sp. n. **A** male antenna **B** male protarsomeres 4–5 in ventral view **C** median lobe in ventral view **D** median lobe in lateral view **E** paramere in external view.

**Figures 19, 20. 19:**
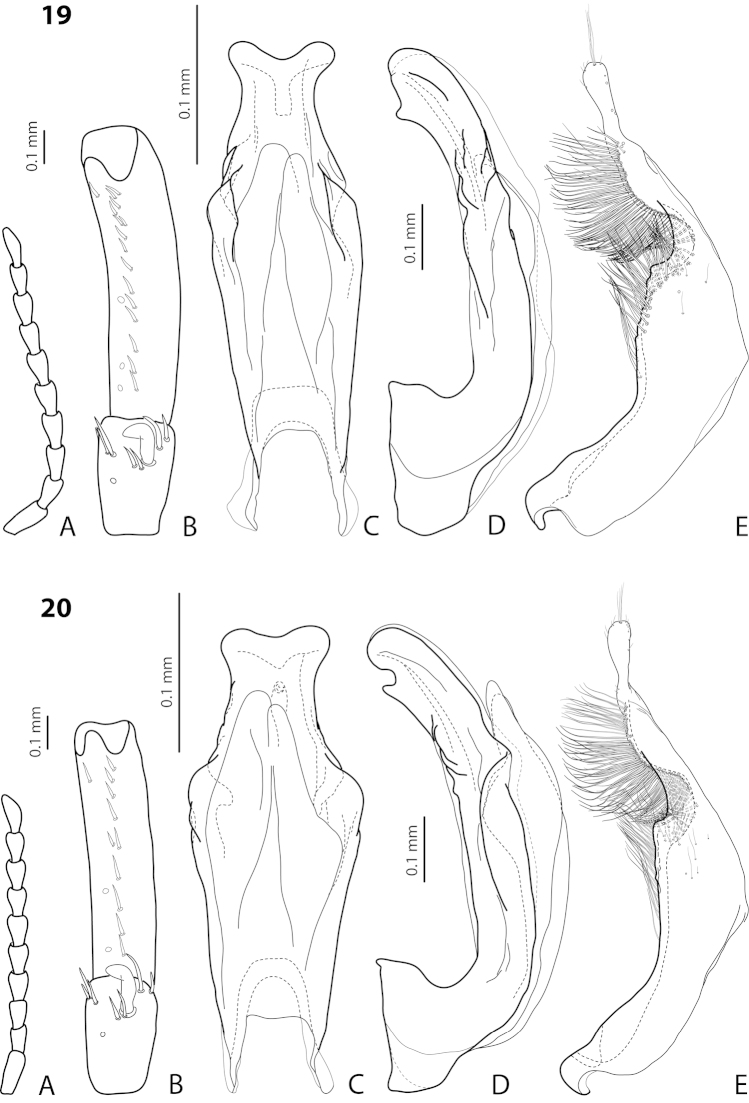
*Exocelina pseudobifida* sp. n. **20** *Exocelina bifida* Shaverdo, Hendrich & Balke, 2012 from Sandaun Province, Papua New Guinea **A** male antenna **B** male protarsomeres 4–5 in ventral view **C** median lobe in ventral view **D** median lobe in lateral view **E** paramere in external view.

**Figure 21.**
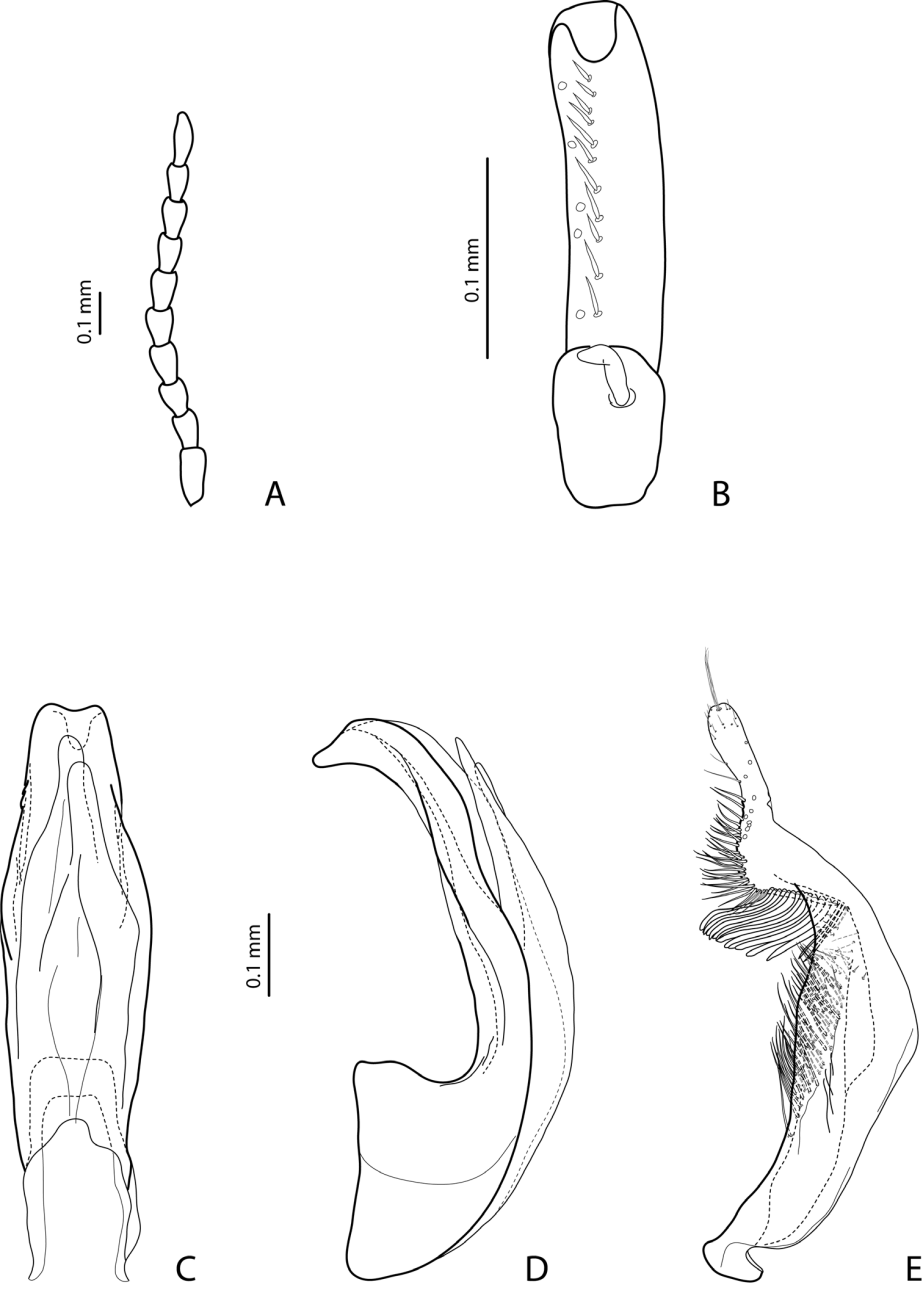
*Exocelina bewaniensis* sp. n. from Bewani, Sandaun Province, Papua New Guinea **A** male antenna **B** male protarsomeres 4–5 in ventral view **C** median lobe in ventral view **D** median lobe in lateral view **E** paramere in external view.

**Figures 22, 23.**
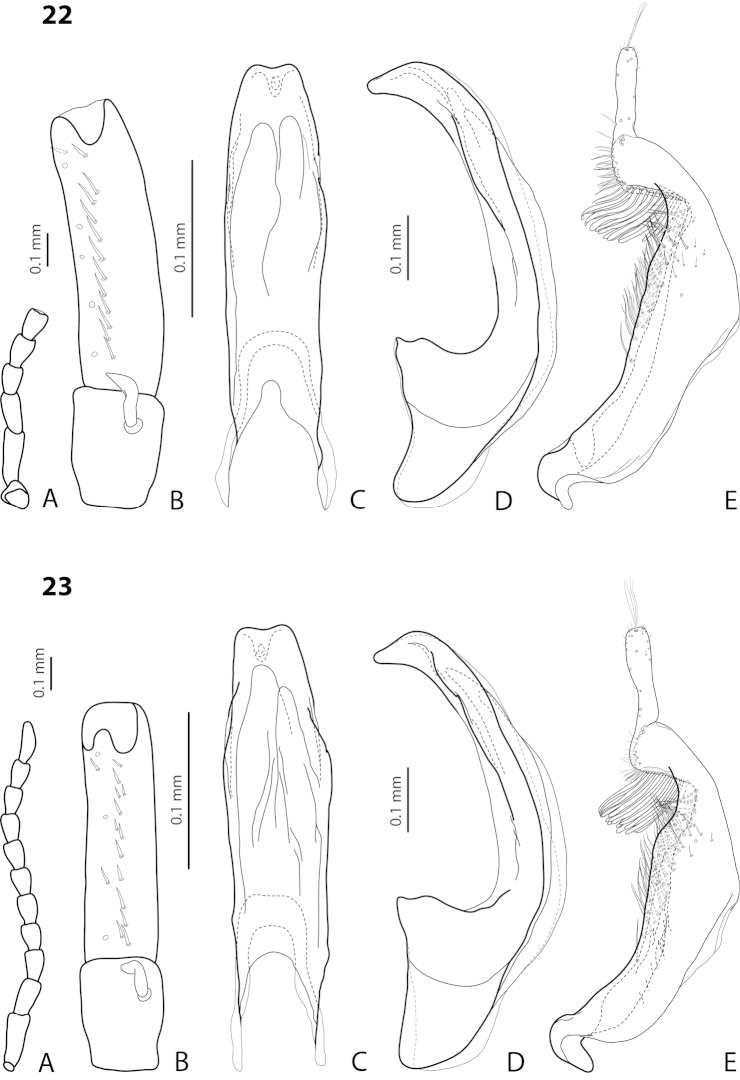
**22** *Exocelina bewaniensis* sp. n. from Noiadi, Mamberamo Raya Regency, Papua Province, Indonesia **23** *Exocelina bewaniensis* sp. n. from Nabire-Enarotali, Nabire/Paniai Regencies, Papua Province, Indonesia **A** male antenna **B** male protarsomeres 4–5 in ventral view **C** median lobe in ventral view **D** median lobe in lateral view **E** paramere in external view.

**Figures 24, 25.**
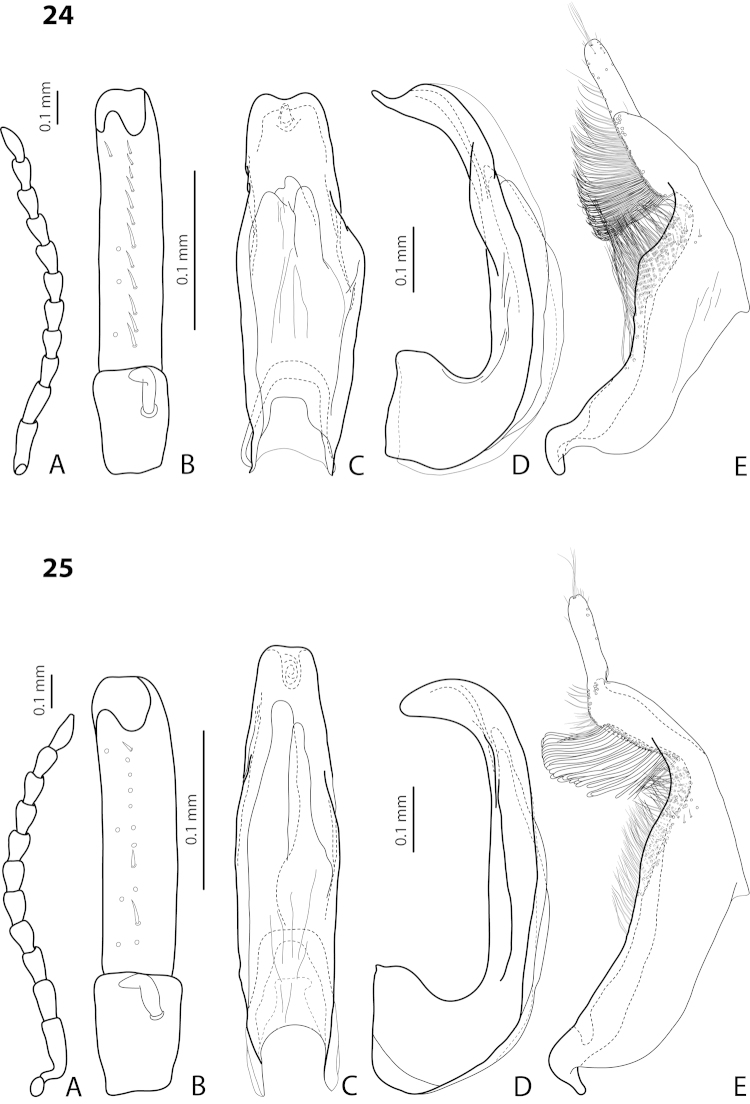
**24** *Exocelina pinocchio* sp. n. **25** *Exocelina mantembu* sp. n. **A** male antenna **B** male protarsomeres 4–5 in ventral view **C** median lobe in ventral view **D** median lobe in lateral view **E** paramere in external view.

**Figures 26, 27.**
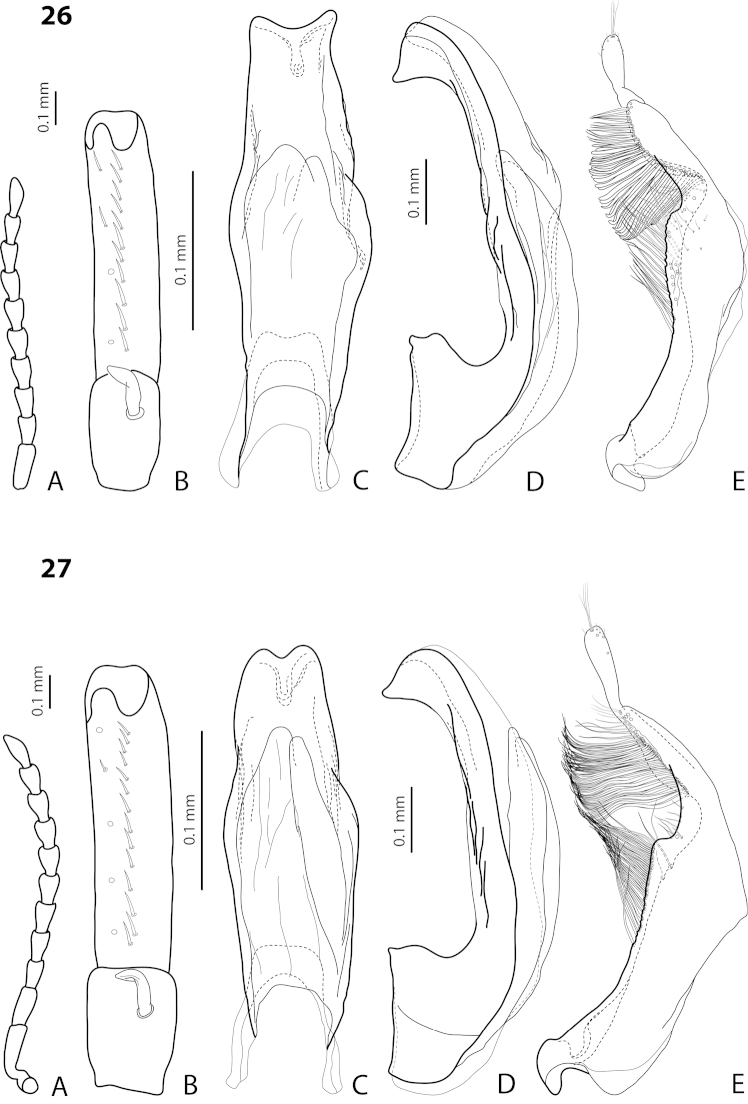
**26** *Exocelina lembena* sp. n. **27** *Exocelina pseudoeme* sp. n. **A** male antenna **B** male protarsomeres 4–5 in ventral view **C** median lobe in ventral view **D** median lobe in lateral view **E** paramere in external view.

**Figures 28–30.**
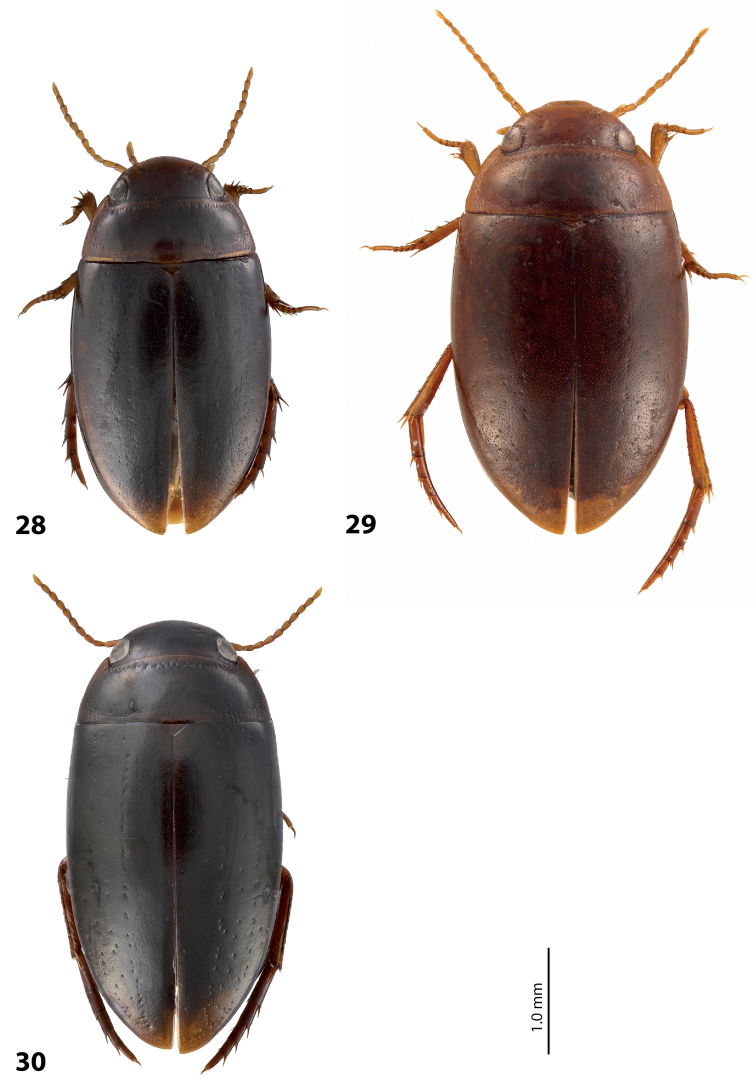
Habitus and coloration. **28** *Exocelina skalei* sp. n. **29** *Exocelina vladimiri* (Shaverdo, Sagata & Balke, 2005) **30** *Exocelina pseudoastrophallus* sp. n.

**Figures 31–34.**
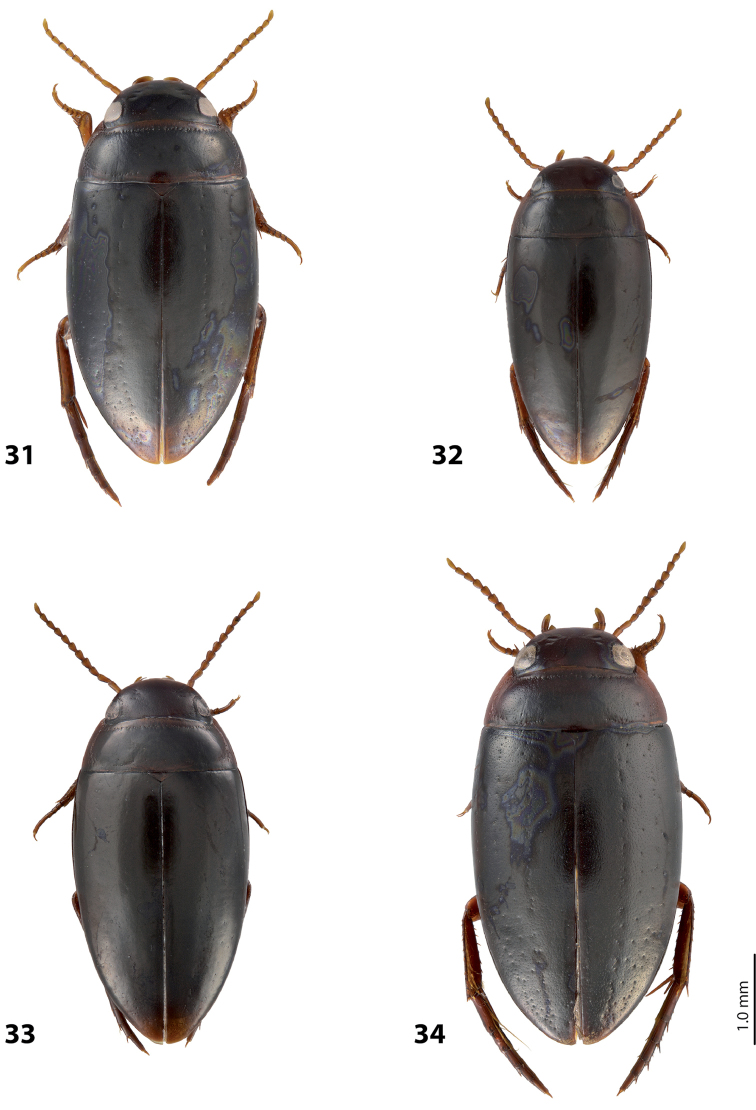
Habitus and coloration. **31** *Exocelina michaelensis* sp. n. **32** *Exocelina craterensis* sp. n. **33** *Exocelina herowana* sp. n. **34** *Exocelina tabubilensis* sp. n.

**Figures 35–38.**
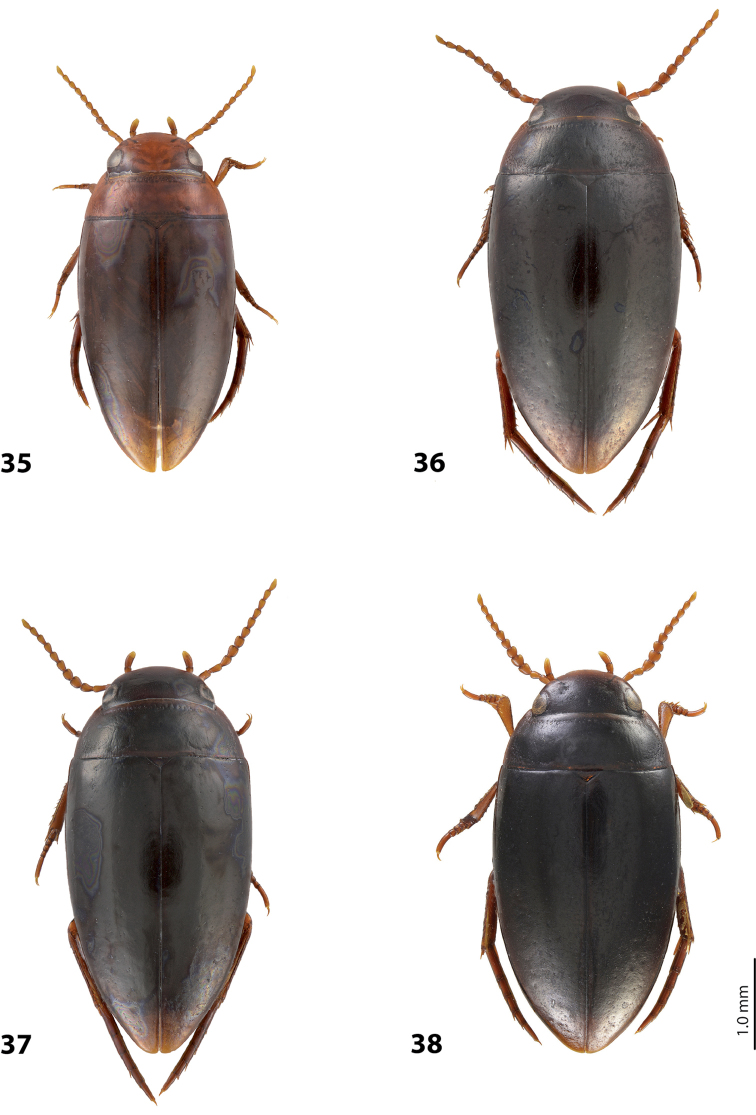
Habitus and coloration. **35** *Exocelina wannangensis* sp. n. **36** *Exocelina jimiensis* sp. n. **37** *Exocelina edeltraudae* Shaverdo, Hendrich & Balke, 2012 **38** *Exocelina pseudoedeltraudae* sp. n. from Shaverdo et al. (2012, fig. 30).

**Figures 39–42.**
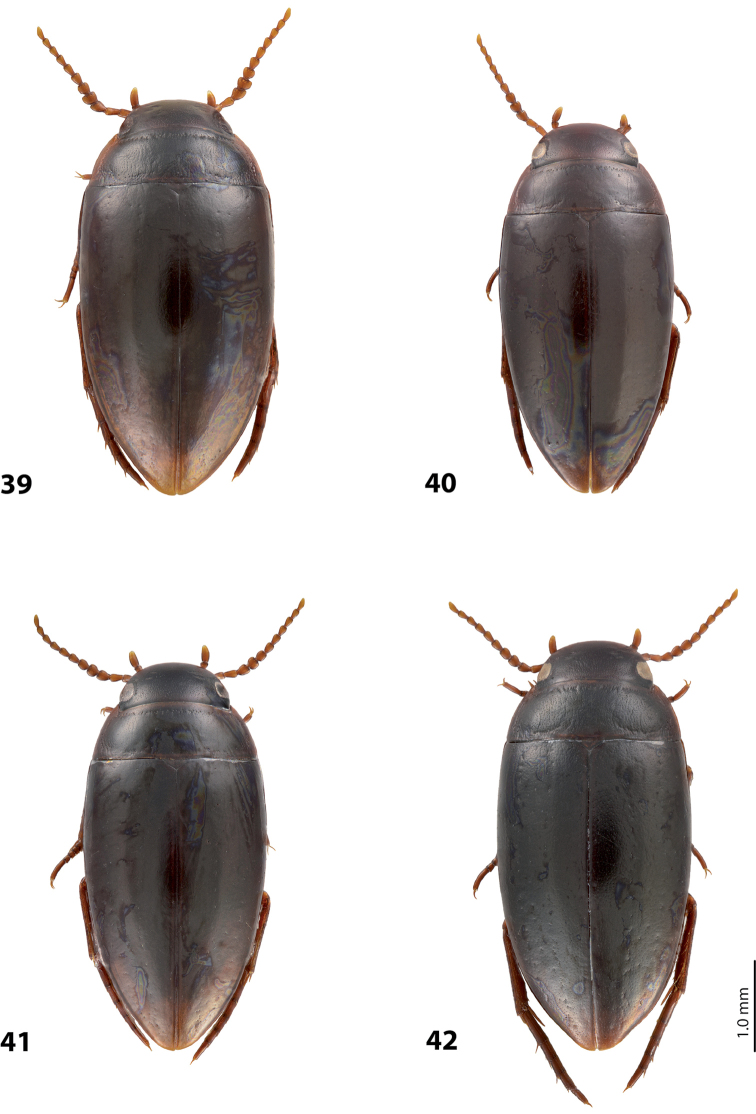
Habitus and coloration. **39** *Exocelina tariensis* sp. n. **40** *Exocelina simbaiarea* sp. n. **41** *Exocelina sandaunensis* sp. n. **42** *Exocelina gorokaensis* sp. n.

**Figures 43–46.**
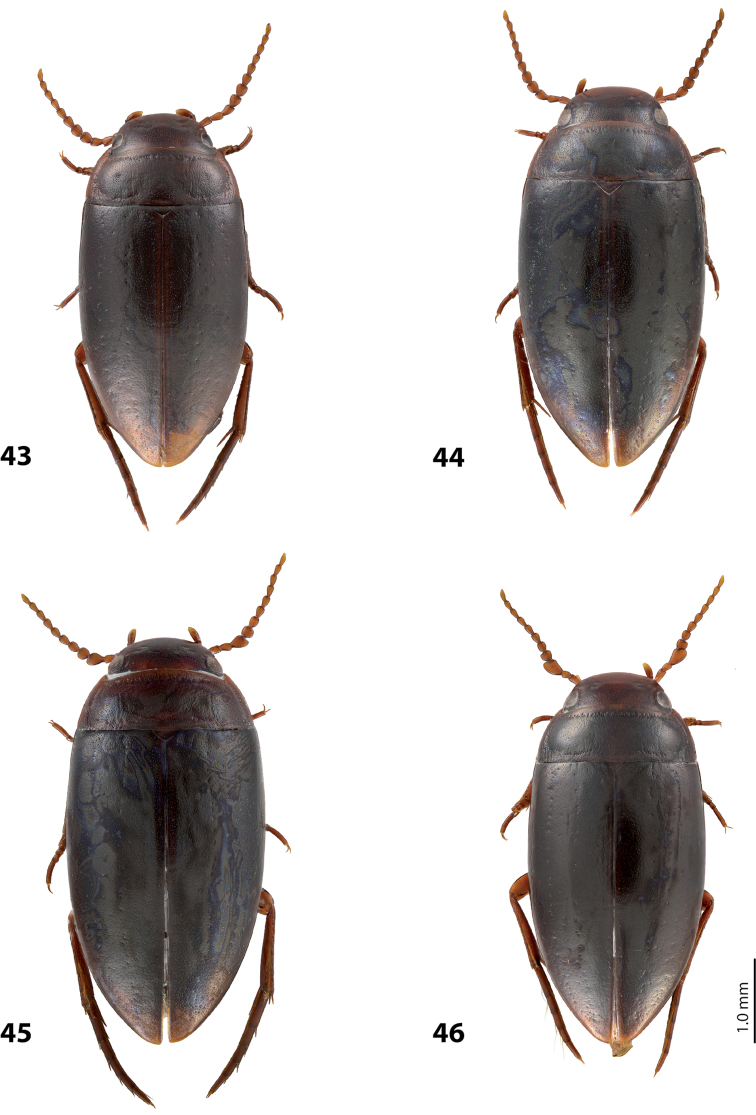
Habitus and coloration. **43** *Exocelina bismarckensis* sp. n. **44** *Exocelina vovai* sp. n. **45** *Exocelina kisli* sp. n. **46** *Exocelina ksionseki* sp. n.

**Figures 47–50.**
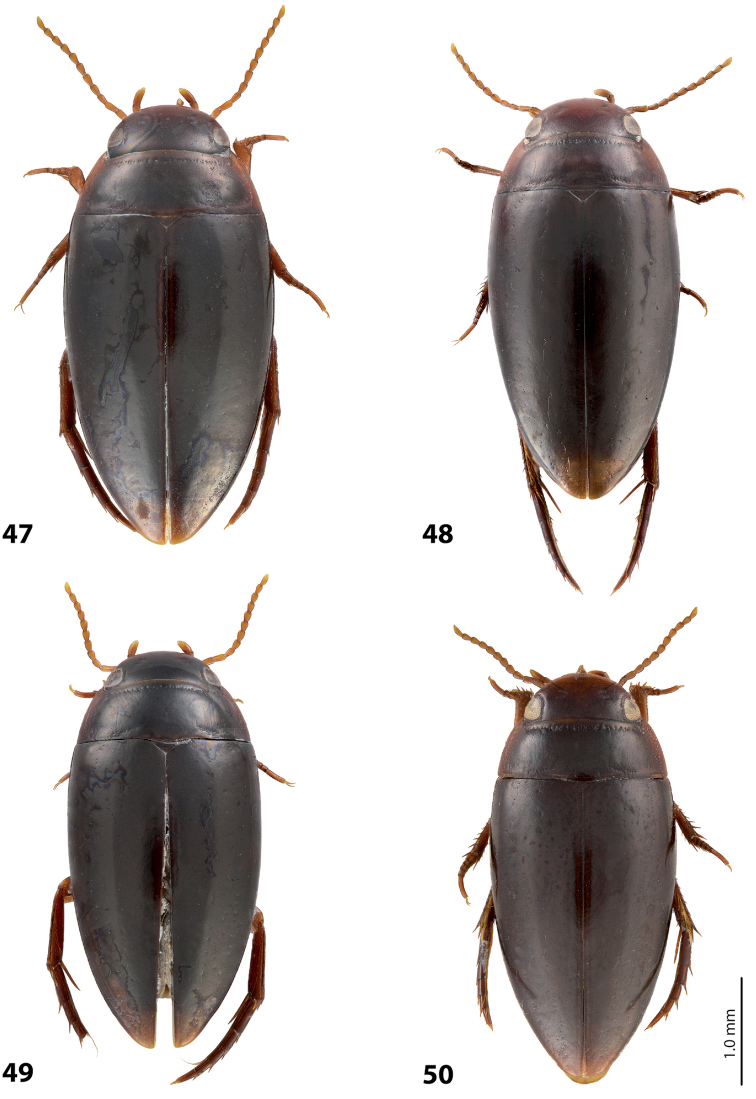
Habitus and coloration. **47** *Exocelina pseudobifida* sp. n. **48** *Exocelina pinocchio* sp. n. **49** *Exocelina bewaniensis* sp. n. **50** *Exocelina mantembu* sp. n.

**Figures 51, 52.**
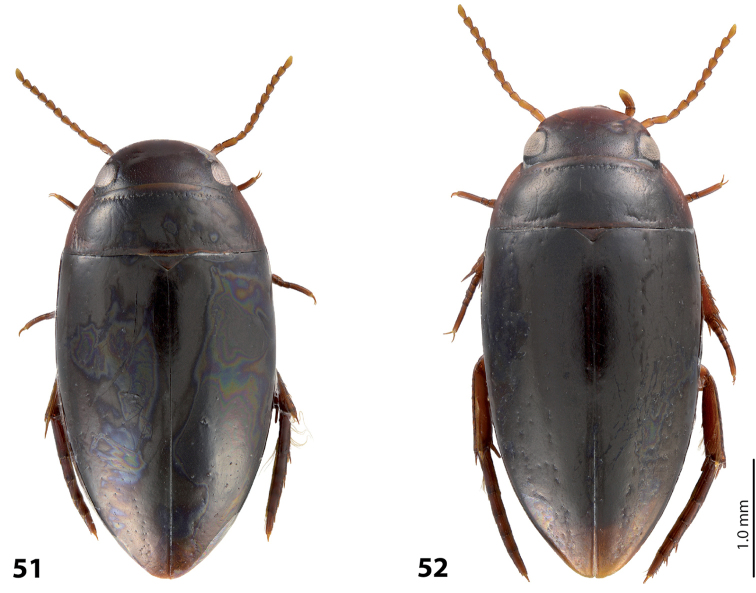
Habitus and coloration. **51** *Exocelina lembena* sp. n. **52** *Exocelina pseudoeme* sp. n.

**Figure 53.**
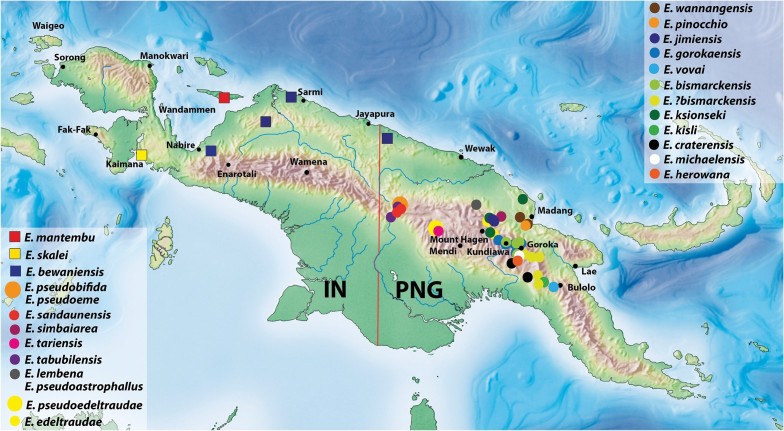
Map of New Guinea showing distribution of species of the *Exocelina ekari*-group treated herein.

**Figure 54.**
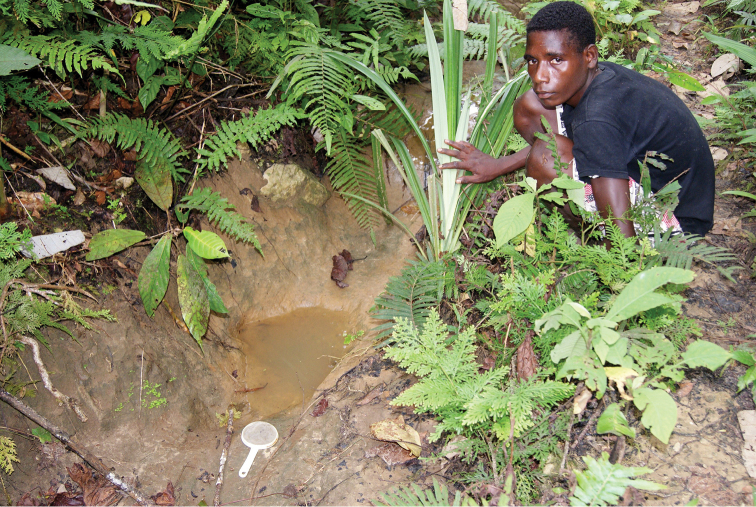
Type locality of the *Exocelina skalei* sp. n.: Indonesia, West Papua Province, Kaimana Regency, near Kamaka Village; photo by A. Skale.

### Habitats

Shaverdo et al. (2012) provided a summary of habitats for New Guinea *Exocelina* species. All the species with only one exception (Shaverdo et al. 2013) are running water associated, but avoid the current, i.e., preferred microhabitats are small, quiet backflows, tiny puddles at the edge of streams and creeks, rock holes filled with water (Fig. 54), and other similar situations. Further information was provided in this wiki site: http://zsm-entomology.de/w/index.php?title=Coleoptera_Fieldwork&oldid=862

## Supplementary Material

XML Treatment for Exocelina bewaniensis

XML Treatment for Exocelina bismarckensis

XML Treatment for Exocelina craterensis

XML Treatment for Exocelina edeltraudae

XML Treatment for Exocelina gorokaensis

XML Treatment for Exocelina herowana

XML Treatment for Exocelina jimiensis

XML Treatment for Exocelina kisli

XML Treatment for Exocelina ksionseki

XML Treatment for Exocelina lembena

XML Treatment for Exocelina mantembu

XML Treatment for Exocelina michaelensis

XML Treatment for Exocelina pinocchio

XML Treatment for Exocelina pseudoastrophallus

XML Treatment for Exocelina pseudobifida

XML Treatment for Exocelina pseudoedeltraudae

XML Treatment for Exocelina pseudoeme

XML Treatment for Exocelina sandaunensis

XML Treatment for Exocelina simbaiarea

XML Treatment for Exocelina skalei

XML Treatment for Exocelina tabubilensis

XML Treatment for Exocelina tariensis

XML Treatment for Exocelina vovai

XML Treatment for Exocelina wannangensis

XML Treatment for Exocelina arfakensis

XML Treatment for Exocelina bifida

XML Treatment for Exocelina brahminensis

XML Treatment for Exocelina knoepfchen

XML Treatment for Exocelina polita

XML Treatment for Exocelina pseudosoppi

## References

[B1] Balke M (1998) Revision of New Guinea *Copelatus* Erichson, 1832 (Insecta: Coleoptera: Dytiscidae): The running water species, Part I. Annalen des Naturhistorischen Museum Wien 100B: 301–341.

[B2] Balke M (1999) Two new species of the genus *Copelatus* Erichson, 1832, subgenus *Papuadytes* Balke, 1998, from Papua New Guinea (Insecta: Coleoptera: Dytiscidae). Annalen des Naturhistorischen Museum Wien 101B: 273–276.

[B3] Broun T (1886) Manual of the New Zealand Coleoptera. Parts III and IV. Government Printer, Wellington, 817–973.

[B4] Larson DJ Alarie Y Roughley RE (2000) Predaceous Diving Beetles (Coleoptera: Dytiscidae) of the Nearctic Region, with emphasis on the fauna of Canada and Alaska. NRC Research Press, Ottawa, Ontario, Canada, 982 pp.

[B5] Miller KB Nilsson AN (2003) Homology and terminology: communicating information about rotated structures in water beetles. Latissimus 17: 1–4.

[B6] Nilsson AN (2013) A world catalogue of the family Dytiscidae, or the diving beetles (Coleoptera, Adephaga). Version 1.I.2013. http://www2.emg.umu.se/projects/biginst/andersn/WCD_20130101.pdf

[B7] Riedel A Sagata K Suhardjono YR Tänzler R Balke M (2013) Integrative taxonomy on the fast track – towards more sustainability in biodiversity research. Frontiers in Zoology 10: . doi: 10.1186/1742-9994-10-15 10.1186/1742-9994-10-15 PMC3626550 23537182

[B8] Shaverdo HV Balke M (2014) *Exocelina kinibeli* sp.n. from Papua New Guinea, a new species of the *E. ullrichi*-group (Coleoptera: Dytiscidae). Koleopterologische Rundschau 84 (accepted).

[B9] Shaverdo HV Sagata K Balke M (2005) Five new species of the genus *Papuadytes* Balke, 1998 from New Guinea (Coleoptera: Dytiscidae). Aquatic Insects 27(4): 269–280. doi: 10.1080/01650420500290169

[B10] Shaverdo HV Surbakti S Hendrich L Balke M (2012) Introduction of the *Exocelina ekari*-group with descriptions of 22 new species from New Guinea (Coleoptera, Dytiscidae, Copelatinae). ZooKeys 250: 1–76. doi: 10.3897/zookeys.250.3715 23378803 10.3897/zookeys.250.3715 PMC3558971

[B11] Shaverdo HV Hendrich L Balke M (2013) *Exocelina baliem* sp. n., the only known pond species of New Guinea *Exocelina* Broun, 1886 (Coleoptera, Dytiscidae, Copelatinae). ZooKeys 304: 83–99. doi: 10.3897/zookeys.304.4852 23794909 10.3897/zookeys.304.4852 PMC3689123

[B12] Toussaint EFA Hall R Monaghan MT Sagata K Ibalim S Shaverdo HV Vogler AP Pons J Balke M (2014) The towering orogeny of New Guinea as a trigger for arthropod megadiversity. Nature Communications 1: 1–10 + 10 supplements, 5:4001. doi: 10.1038/ncomms5001 10.1038/ncomms5001 24874774

[B13] Wikipedia, the free encyclopedia (2014a) West Papua (province). http://en.wikipedia.org/wiki/West_Papua_(province)

[B14] Wikipedia, the free encyclopedia (2014b) Papua (province). http://en.wikipedia.org/wiki/Papua_(province)

[B15] Wikipedia, the free encyclopedia (2014c) Administrative divisions of Papua New Guinea. http://en.wikipedia.org/wiki/Administrative_divisions_of_Papua_New_Guinea

